# Development of Mild Palladium‐Catalyzed N‐Arylation of Ortho‐Acylanilines in Rhamnolipid Micellar System

**DOI:** 10.1002/cssc.70609

**Published:** 2026-04-12

**Authors:** Attila R. Herczegh, Péter R. Kollár, Johannes Lehmann, Zoltán Novák

**Affiliations:** ^1^ MTA‐ELTE “Lendület” Catalysis and Organic Synthesis Research Group, Institute of Chemistry Eötvös Loránd University Budapest Hungary; ^2^ Research Development & Innovation Evonik Operations GmbH Hanau‐Wolfgang Germany

**Keywords:** micellar catalysis, Pd‐catalyzed C—N bond formation, rhamnolipids, surfactant, sustainable

## Abstract

We developed a green and efficient method for Pd‐catalyzed N‐arylation of ortho‐acylanilines in water using rhamnolipid‐based micellar catalysis. The (cinnamylPdCl)_2_/^t^BuXPhos system enables high yields at 40°C, significantly lower than conventional methods (>100°C), with broad substrate scope. This industrially produced biodegradable surfactant system offers a sustainable alternative to organic solvents, combining enhanced reactivity with improved energy efficiency and environmental compatibility.

## Graphical abstract

Aqueous micellar catalysis using industrially sourced rhamnolipids enables efficient Pd‐catalyzed *N*‐arylation of *o*‐acylanilines at 40°C. This surfactant‐driven system offers a sustainable and more efficient alternative to organic solvents, combining broad substrate scope, low catalyst loading, and enhanced energy efficiency.

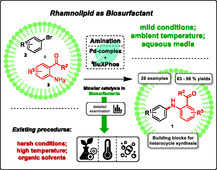

## 1 Introduction

**SCHEME 1:**
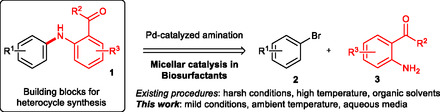
N‐arylation strategy based on palladium‐catalyzed Buchwald–Hartwig amination.

N‐aryl‐aminoacetophenones and N‐aryl‐aminobenzophenones are valuable building blocks in the synthesis of acridines and acridones [1, 2, 3, 4, 5], which are important in medicinal chemistry for their antimicrobial [6, 7], anticancer [8], and anti‐inflammatory [9, 10, 11] properties. A common approach for the synthesis of aminoacetophenones and aminobenzophenones involves palladium‐catalyzed Buchwald–Hartwig cross‐coupling reactions between aryl bromides and the corresponding aniline derivatives with diverse substitution patterns (Scheme 1). While these palladium‐catalyzed methods offer efficient syntheses with broad functional group tolerance [12, 13, 14, 15, 16], they often require relatively harsh conditions such as elevated temperatures, and the use of strong bases or high catalyst loadings, all of which are not ecologically friendly and can limit substrate scope and scalability. These limitations highlight the need for milder and more sustainable synthetic alternatives, especially for sensitive or multifunctional substrates.

The use of micellar catalytic systems could offer an alternative strategy enabling and reaction conditions that require lower energy investment and lower palladium catalyst loading, which is an important aspect of green chemistry. The challenges associated with the harsh conditions of traditional palladium‐catalyzed amination reactions performed in organic media can be effectively addressed through the application of aqueous micellar catalysis.

Moreover, water is one of the most accessible and environmentally benign solvents in organic chemistry, due to its nontoxic, nonflammable nature, low cost, and high abundance. Although the use of water as a reaction medium aligns closely with the principles of green chemistry, a major limitation of its use as solvent in organic synthesis lies in its incompatibility with many organic reagents and catalysts, which often exhibit poor solubility in aqueous media. To overcome this challenge, innovative strategies such as “on water” reactions [17, 18, 19, 20, 21, 22, 23, 24, 25] and the use of surfactant‐based micellar systems were developed in the last decades [26, 27, 28, 29, 30, 31, 32]. In micellar catalysis, amphiphilic surfactants self‐assemble into micelles that encapsulate hydrophobic reactants within their cores, creating a unique microenvironment that can dramatically enhance the rate and selectivity of organic transformations. Thanks to the beneficial effect of these systems, micellar catalysis has emerged as a powerful tool in aqueous‐phase organic synthesis, including transition metal‐catalyzed processes relevant to the pharmaceutical and agrochemical industries [33, 34, 35, 36, 37, 38, 39, 40, 41, 42, 43, 44, 45, 46, 47, 48, 49, 50, 51, 52, 53, 54, 55, 56, 57, 58]. Traditionally, synthetic surfactants are employed in these transformations. Besides their effective use, they raise concerns related to economy, sustainability, biodegradability, and environmental persistence. As an alternative, attention has turned toward biosurfactants, which are naturally derived surface‐active agents produced through microbial fermentation [59, 60, 61]. Biosurfactants offer several advantages as they are not only biodegradable [62, 63], but display an exceptionally low aquatic toxicity and can be synthesized from renewable feedstocks, including agricultural waste and other organic residues. In recent years, Vinayagam and co‐workers efficiently used natural saponin as surfactant for aqueous micellar catalysis and demonstrated its applicability in palladium‐catalyzed Suzuki [64] and amination [65] reactions.

**FIGURE 1:**
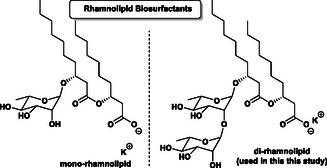
General structure of mono‐ and di‐rhamnolipids.

Among the various classes of biosurfactants, rhamnolipids have attracted significant interest [66, 67, 68]. In nature, these species are produced mainly by Pseudomonas aeruginosa [69]. The wild type rhamnolipids consist of one or two rhamnose sugar units linked to two β‐hydroxy fatty acid chains, forming amphiphilic molecules such as mono‐rhamnolipid and di rhamnolipid (Figure 1). Due to their combination of superior solubilization with excellent environmental compatibility and mildness toward skin application they have successfully been applied in the fields of environmental remediation [70, 71, 72, 73], cosmetics [74], food processing [75, 76], and pharmaceuticals [69, 77, 78, 79, 80, 81, 82]. Evonik has recently launched a di‐rhamnolipid bearing mainly two β‐hydroxy decanoic chains, produced on multi ton scale for household applications by fermentation based on sugar as solely carbon source [83].

Despite their proven performance in other industries, the application of rhamnolipids in micellar organic synthesis and catalysis remains largely unexplored. Very recently, we have demonstrated their applicability in micellar catalysis [84], and we aimed to exploit their potential in organic synthesis considering the growing demand for sustainable and environmentally responsible chemical processes. In our recent work, we aimed to expand the applicability of the efficient biosurfactant based micellar catalytic system for the palladium‐catalyzed N‐arylation of acylanilines with aryl bromides (Scheme 1) under mild reaction conditions with low Pd catalyst loading.

## 2 Results and Discussion

For the optimization of the palladium‐catalyzed amination reaction, we selected bromobenzene and 2‐aminoacetophenone as coupling partners. The model reactions were performed at 40°C in a 4 w/w% aqueous rhamnolipid solution using KOH as strong base required for the Buchwald–Hartwig amination and deprotonate the lipids carboxylic group to maintain micelle integrity [85]. First, several commonly used palladium sources were screened, including Pd_2_(dba)_3_, (allylPdCl)_2_, and (cinnamylPdCl)_2_, in the presence of ^t^BuXPhos ligand (Table 1, entries 1–3). Pd_2_(dba)_3_ afforded 81% conversion after 4 h, while (allylPdCl)_2_ showed poor reactivity with only 17% conversion. In contrast, (cinnamylPdCl)_2_ provided 92% conversion under identical conditions and reach complete conversion after 18 h (Entry 4), making it the most effective catalyst precursor. Using the same catalyst system, we repeated the reaction at rt and 60°C, and we observed 91% conversion after 18 h (Entry 5), and 99% conversion after 4 h (Entry 6). For further studies, we selected (cinnamylPdCl)_2_ as palladium source and performed the couplings at 40°C, considering as optimal reaction temperature and reaction time.

**TABLE 1:** Optimization of catalyst system for the Pd‐catalyzed amination in Rhamnolipid solution.^a^

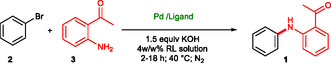					
Entry	Pd loading/ppm	Pd source	Ligand	Time, h	Conv., %^b^
1	500	Pd_2_dba_3_	^t^BuXPhos	4	81
2	500	(AllylPdCl)_2_	^t^BuXPhos	4	17
3	500	(cinnamylPdCl)_2_	^t^BuXPhos	4	92
4	500	(cinnamylPdCl)_2_	^t^BuXPhos	18	100
5	500	(cinnamylPdCl)_2_	^t^BuXPhos	18	91^c^
6	500	(cinnamylPdCl)_2_	^t^BuXPhos	4	99^d^
7	500	(cinnamylPdCl)_2_	BippyPhos	18	85
8	500	(cinnamylPdCl)_2_	XPhos, PCy_3_·HBF_4_, QPhos, dppf, P(^t^Bu)_3_, PPh_3_	4	0
9	1000	(cinnamylPdCl)_2_	^t^BuXPhos	4	100
10	250	(cinnamylPdCl)_2_	^t^BuXPhos	4	63
11	250	(cinnamylPdCl)_2_	^t^BuXPhos	18	92
12	50	(cinnamylPdCl)_2_	^t^BuXPhos	18	20
13	500	(cinnamylPdCl)_2_	^t^BuXPhos	2	100^e^

a Standard reaction conditions: Bromobenzene (0.5 mmol), 2‐aminoacetophenon (1.2 equiv.), Pd source (50–1000 ppm), ligand (200–2000 ppm), KOH (1.5 equiv.) in 1 mL degassed surfactant solution, 40°C, N_2_ atmosphere.

b Conversion was determined with GC–MS.

c Reaction was performed at rt.

d Reaction was performed on 60°C.

e Precomplexed (cinnamylPdCl)_2_/^t^BuXPhos stock solution was used.

Next, various ligands beyond ^t^BuXPhos were evaluated to identify the most suitable ligand for the amination reaction performed in the rhamnolipid‐based micellar system.

Under the previously used catalytic conditions, BippyPhos also showed good performance with 85% conversion in 18 h at 40°C (Entry 7). However, other tested ligands, including XPhos, PCy_3_·HBF_4_, QPhos, dppf, P(^t^Bu)_3_, and PPh_3,_ were formed completely inactive catalyst systems, providing no detectable product under the applied reaction conditions (Entry 8). This phenomenon could be explained with the lower catalytic power of the corresponding Pd‐phosphine system at relatively low reaction temperatures.

After finding the most efficient catalyst system, we investigated the effect of catalyst loading higher and lower than 500 ppm Pd complex, used in the previous experiments. Reactions were conducted using 1000 ppm, 250 ppm, and 50 ppm of the (cinnamylPdCl)_2_ complex together with 4 equivalents of ^t^BuXPhos ligand. Complete conversion of the amination reaction was achieved at 1000 ppm Pd‐complex loading after 4 h (Entry 9). However, lowering the amount of catalyst to 250 ppm or 50 ppm resulted in significantly reduced conversion, and the reaction did not reach complete conversion even after 18 h (Entries 11 and 12). We concluded that at least 500 ppm catalyst loading is required to maintain high efficiency under these mild reaction conditions.

To further improve catalytic performance and reproducibility, a preformed catalyst stock solution was prepared by combining (cinnamylPdCl)_2_ and ^t^BuXPhos in 4 w/w% rhamnolipid solution. Using this stock solution at 500 ppm Pd‐complex loading under optimized conditions resulted in complete conversion within 2 h (Entry 13). In comparison, the same reaction using separately added catalyst and ligand gave only 68% conversion after 2 h (not shown in table). It is of note, the stock solution remained catalytically active after 1 week of storage at room temperature, demonstrating its excellent stability and operational convenience for repetitive or scaled‐up applications.

The influence of rhamnolipid concentration on the reaction performance was also examined (Table 2). Coupling reactions were carried out in rhamnolipid solutions of 1, 2, 4, and 5 w/w%, as well as in pure water (Entries 1–5). While all rhamnolipid‐containing reactions reached 100% conversion after 18 h, reaction rates were considerably faster in 4 and 5 w/w% rhamnolipid solutions compared to 1 and 2 w/w% on the basis of conversion data after 6 h.

**TABLE 2:** Study of solvent effect on the Pd‐catalyzed amination.^a^

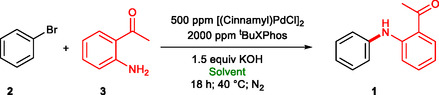				
Entry	Solvent	Surfactant concentration, w/w%	Conversion after 6 h, %^b^	Conversion after 18 h, %^c^
1	Rhamnolipids	5 w/w%	97	100
2	Rhamnolipids	4 w/w%	96	100
3	Rhamnolipids	2 w/w%	86	100
4	Rhamnolipids	1 w/w%	84	100
5	water‐	0 w/w% (pure water)	57	72
6	TPGS‐750‐M	4 w/w%	96	100
7	Tween 80	4 w/w%	97	100
8	Kolliphor	4 w/w%	85	93
9	^t^BuOH,	—	6	6
10	Dioxane	—	3	4
11	Toluene	—	0	0
12	neat‐	—	58	71 (36 h)

a Standard reaction conditions: Bromobenzene (0.5 mmol), 2‐aminoacetophenon (1.2 equiv.), (cinnamylPdCl)_2_ (500 ppm), ^t^BuXPhos (2000 ppm)**,** KOH (1.5 equiv.) in 1 mL degassed surfactant solution or organic solvent, 40°C, N_2_ atmosphere.

b Samples were taken at two different indicated times.

c Conversions measured at given reaction times, and they were determined with GC–MS.

In contrast, the reaction in pure water reached only 72% conversion after 18 h (Entry 5) and did not improve beyond 80% even after 48 h (data not shown in Table 2), indicating that the presence of rhamnolipid micelles is essential for achieving complete conversion within practical timeframes.

A comparative study with alternative surfactants such as TPGS‐750‐M, Tween 80, Kolliphor was also performed, using the optimized reactions conditions and the most efficient catalyst system (Table 2, Entries 6–8). With the exception of Kolliphor, all surfactants enabled the reaction to proceed full conversion after 18 h confirming that the rhamnolipid biosurfactant‐based medium offers sustainable alternative to known chemically produced surfactants.

To assess the practical advantage of the rhamnolipid micellar system with organic media, the efficiency of the palladium‐catalyzed amination was compared with conventional organic solvents such as *tert*‐butanol, dioxane, and toluene, as well as under neat conditions (Entries 9–12). Surprisingly, the reaction showed no significant progress in any of the organic solvents at 40°C, yielding less than 6% conversion in ^t^BuOH after 18 h (Entry 9). Although coupling under neat reaction conditions gave 58% conversion after 6 h, the reaction reached only 71% conversion after 36 h reaction time (Entry 12). These findings highlight a critical advantage of the aqueous rhamnolipid system over organic solvents. The micellar media allows efficient catalysis at significantly lower temperatures without the need for organic solvents, representing an important benefit from green chemistry and sustainability perspective.

**SCHEME 2:**
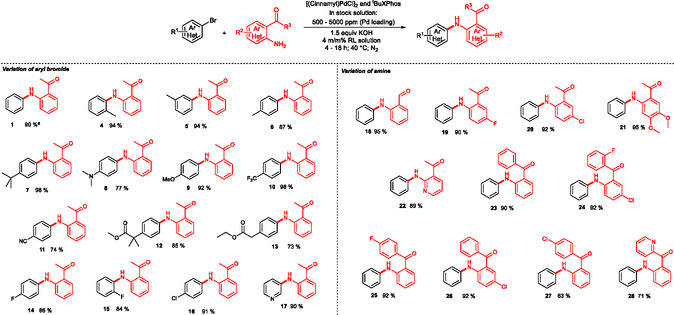
Scope of the Pd‐catalyzed amination reaction of aryl halides and amines. Standard reaction conditions: aryl bromide (2 mmol), aromatic amine (1.05 equiv.), stock solution of [(cinnamyl)PdCl)_2_] and ^t^BuXPhos, KOH (1.5 equiv.), 3.8 mL degassed rhamnolipids solution, N_2_ atmosphere. 40°C. a 5 mmol ArBr scale.

Under the optimized reaction conditions—using a stock solution of palladium complex made from (cinnamylPdCl)_2_ and ^t^BuXPhos in 4 w/w% aqueous rhamnolipid solution, 1.5 equivalents of KOH, at 40°C for 18 h—the scope and limitations of the micellar Pd‐catalyzed N‐arylation were systematically investigated (Scheme 2). The model reaction between bromobenzene and 2‐aminobenzophenone provided the diarylamine product (**1**) in 90% isolated yield (5 mmol scale), establishing a reliable benchmark for further substrate exploration. To evaluate the compatibility of various aryl bromides, 2‐aminoacetophenone was employed as a nucleophilic partner in the palladium‐catalyzed coupling reaction. Electron‐rich substrates such as 2‐, 3‐, and 4‐bromotoluene were smoothly converted to the corresponding products **4**, **5,** and **6** in 94%, 94%, and 87% yields, respectively. The presence of a bulky electron‐donating tert‐butyl group in the para‐position, in case of 4‐tert‐butylbromobenzene, had no detrimental effect on reactivity, yielding the coupled product (**7**) in 98% isolated yield. Moreover, highly electron‐rich substrates, such as a *para*‐dimethylamino‐, and *para*‐methoxybromobenzene, were transformed efficiently, affording products **8** and **9** in 77% and 92% yield. Electron‐deficient arylbromides such as *para*‐cyano‐ and *para*‐trifluoromethylbromobenzene also showed excellent compatibility, giving the desired amines (**10** and **11**) in 98% and 74% yield, respectively.

Importantly, functional groups sensitive to nucleophilic or basic aqueous environments, such as esters, remained intact under the micellar conditions. The coupling reaction with an aryl bromide bearing an ester moiety proceeded smoothly, affording products (**12** and **13**) in 85% and 73% yield without signs of hydrolysis. Halogen substituents, such as fluoro and chloro groups, were also tolerated, and the corresponding products **14**, **15,** and **16** were isolated in yields ranging from 84% to 91%. The method was further extended to heteroaryl bromide, 3‐bromopyridine as a representative heterocyclic electrophile and the coupling afforded the desired N‐arylated product (**17**) in 90% yield.

In the second part of the substrate scope, the reactivity of various aniline nucleophiles was evaluated using bromobenzene as the electrophilic partner.

2‐Aminobenzaldehyde afforded the corresponding diarylamine (**18**) in 95% isolated yield, indicating the stability of the formyl group under the reaction conditions. Substituted aminoacetophenones provided the desired N‐arylated products (**19**–**22**) in excellent yields ranging from 90% to 95%. Similarly, 2‐aminobenzophenone was efficiently arylated, yielding product **23** in 90% isolated yield. The method also tolerated the presence of chloro‐ and fluoro‐ functions on the aniline fragment and coupling reactions afforded the desired products (**24**–**27**) in 63–92% yields, demonstrating the functional group tolerance of the catalytic system.

Pyridyl‐containing amino substrates were also suitable for this transformation. Under the optimized aqueous micellar conditions, these heterocyclic amines gave the desired N‐arylated products (**22** and **28**) in 89% and 71% yields, respectively. The results of the scope and limitation study demonstrate the broad applicability and robustness of the developed micellar catalytic system, which enables the efficient green N‐arylation of a wide range of amines and aryl halides in water under mild reaction conditions.

## 3 Conclusion

In summary, we have developed a highly efficient and environmentally benign method for the Pd‐catalyzed N‐arylation of aminoacetophenones and aminobenzophenones utilizing micellar catalysis of aqueous di‐rhamnolipid solutions. The use of a preformed (cinnamylPdCl)_2_/^t^BuXPhos stock solution in 4 w/w% rhamnolipid media enabled efficient coupling reactions under mild conditions (40°C) with catalyst loading down to 500 ppm. Optimization studies highlighted the critical role of the ligand, the surfactant concentration, and the catalyst preformation in maximizing reactivity. The protocol showed broad substrate scope and excellent functional group tolerance, including heterocycles and sensitive moieties such as esters, aldehydes, and halogens. Notably, the reactions performed poorly in conventional organic solvents under ambient reaction conditions, emphasizing the unique advantage of the micellar system in facilitating green and energy‐efficient transformations. This methodology offers a sustainable alternative to traditional cross‐coupling reactions for the synthesis of the target building blocks and demonstrates the promising utility of biosurfactants in modern catalytic processes.

## Supporting Information

Additional supporting information can be found online in the Supporting Information section. The authors have cited additional references within the Supporting Information [87–92]. **Supporting Table S1**: Measurement table for solutions of different concentrations for 100 ml of solution.

## Funding

This study was supported by the Nemzeti Kutatási Fejlesztési és Innovációs Hivatal (K143439) and Magyar Tudományos Akadémia (Lendület LP2023‐12/2023).

## Conflicts of Interest

The authors declare no conflicts of interest.

## Supporting information

Supplementary Material

## Data Availability

The data that supports the findings of this study are available in the supplementary material of this article**.**
