# Supplementary material for: Development of Mild Palladium‐Catalyzed N‐Arylation of Ortho‐Acylanilines in Rhamnolipid Micellar System
Source: ChemSusChem. 2026 Apr 12;19(7):e70609. doi: 10.1002/cssc.70609 (PMC13071218; doi:10.1002/cssc.70609)
Supplement: Supplementary file 1 — Supplementary Material [file CSSC-19-e70609-s001.pdf]

Development of Mild Palladium-Catalyzed N-Arylation of ortho-Acylanilines in Rhamnolipid Micellar System

Electronic Supplementary Information

Attila R. Herczegh,[a] Péter R. Kollár,[a] Johannes Lehmann,[b] Zoltán Novák*[a]

[a]Catalysis and Organic Synthesis Research Group, Institute of Chemistry, Eötvös Loránd University, Pázmány Péter stny. 1/A, H-1117 Budapest, Hungary

[b]Research, Development & Innovation Evonik Operations GmbH
Rodenbacher Chaussee 4, 63457 Hanau-Wolfgang, Germany

1. Table of Contents

1. Table of Contents 2

2. General information 4

3. Optimization experiments 6

3.1 Optimization of Pd catalyst loading 6

3.2 Ligand screening 7

3.3 Optimization of Pd catalyst loading 8

3.4 Effect of Rhamnolipids solution concentration on the reaction 9

3.5 Comparison of reactions in various surfactant solutions 10

3.6 Comparison with organic solvents 11

3.7 Comparison of different bases 12

3.8 Temperature screening 12

3.9 Catalyst stock-solution in RL 13

3.10 Comparison of ^{t}BuXPhos-Pd-G3 and catalyst stock solution 14

3.11 Substrate scope 15

3.12 Green metric analysis 16

4. Synthesis 19

4.1 General preparations and syntheses 19

4.1.1 Preparation of rhamnolipid solutions 19

4.1.2 Preparation of other surfactant solutions 19

4.1.3 Preparation of catalyst stock solution 19

4.1.4 General synthesis of Buchwald-Hartwig amination for 2 mmol product 19

4.2 Recipes and characterization of Buchwald-Hartwig-products 20

5 mmol scale reaction of 1-(2-(phenylamino)phenyl)ethan-1-one (1) 20

1-(2-(*o*-tolylamino)phenyl)ethan-1-one (4)[87] 21

1-(2-(*m*-tolylamino)phenyl)ethan-1-one (5) 21

1-(2-(*p*-tolylamino)phenyl)ethan-1-one (6) 22

1-(2-((4-(*tert*-butyl)phenyl)amino)phenyl)ethan-1-one (7) 22
1-(2-((4-(dimethylamino)phenyl)amino)phenyl)ethan-1-one (8) 22
1-(2-((4-methoxyphenyl)amino)phenyl)ethan-1-one (9)[88] 23
1-(2-((4-(trifluoromethyl)phenyl)amino)phenyl)ethan-1-one (10)[88] 23
4-((2-acetylphenyl)amino)benzonitrile (11) 24
Methyl 2-(4-((2-acetylphenyl)amino)phenyl)-2-methylpropanoate (12) 24
Ethyl 2-(4-((2-acetylphenyl)amino)phenyl)acetate (13) 25
1-(2-((4-fluorophenyl)amino)phenyl)ethan-1-one (14)[88] 25
1-(2-((2-fluorophenyl)amino)phenyl)ethan-1-one (15)[88] 26
1-(2-((4-chlorophenyl)amino)phenyl)ethan-1-one (16)[88] 26
1-(2-(pyridin-3-ylamino)phenyl)ethan-1-one (17) 27
2-(phenylamino)benzaldehyde (18)[88] 27
1-(5-fluoro-2-(phenylamino)phenyl)ethan-1-one (19) 28
1-(5-chloro-2-(phenylamino)phenyl)ethan-1-one (20) 28
1-(4,5-dimethoxy-2-(phenylamino)phenyl)ethan-1-one (21)[88] 28
1-(2-(phenylamino)pyridin-3-yl)ethan-1-one (22) 29
Phenyl(2-(phenylamino)phenyl)methanone (23)[88] 29
(5-chloro-2-(phenylamino)phenyl)(2-fluorophenyl)methanone (24) 30
(4-fluorophenyl)(2-(phenylamino)phenyl)methanone (25) 30
(5-chloro-2-(phenylamino)phenyl)(phenyl)methanone (26)[88] 31
(4-chlorophenyl)(2-(phenylamino)phenyl)methanone (27)[92] 31
(2-(phenylamino)phenyl)(pyridine-2-yl)methanone (28) 31
5. NMR Spectra 33
6. References 65

2. General information

Unless otherwise indicated, starting materials were obtained from commercial suppliers, and were used without further purification. 50 % aqueous Rhamnolipids solution (REWOFERM RL 100) was kindly provided by Evonik. Di-Rhamnolipid has not been commercialized yet, Evonik is providing test samples of di-rhamnolipid to anyone interested in exploring novel applications for it upon request. The surfactant solutions were prepared using degassed distilled water and were stored under nitrogen. Purification of the obtained products was carried out with a Teledyne ISCO Combiflash Nextgen 300+ Flash Chromatography system using Teledyne ISCO Redisep Gold or Bronze normal phase columns and n-hexane and ethyl acetate as eluents.

The solid materials were measured on an OHAUS Pineer analytical balance, the liquids were measured airtight with Hamilton syringes.

Analytical thin-layer chromatography (TLC) was performed on Merck DC precoated TLC plates with 0.25 mm Kieselgel 60 F 254. Visualization was performed with a 254 nm UV lamp.

The ^{1}H, ^{19}F and ^{13}C NMR spectra were recorded on a Bruker Ascend 400 MHz spectrometer. Solvents' residual proton peaks were used as standards. Chemical shifts (δ) are reported in ppm, coupling constants (J) are reported in Hertz (Hz). Splitting patterns are designated as s (singlet), bs (broad singlet), d (doublet), t (triplet), q (quartet), h (hextet), hept (heptet) and m (multiplet).

All melting points were measured on a Bibby Scientific SMP10 apparatus and are uncorrected.

Conversions were determined on an Agilent 5890 Gas Chromatograph (30 m × 0.25 mm column with 0.25 μm HP-5MS coating, N_2 carrier gas) with FID detector.

The conversions (η) given in the publication were calculated from the ratio of the area under the peak of the limiting reagent (A_{limiting}) to the area under the peak of the product (A_{product}) using ***1. equation*** below.

$$\eta = \frac{A_{\text{product}}}{A_{\text{product}} + A_{\text{limiting}}} \cdot 100$$

1. equation: The equation for the conversion calculations.

Low resolution mass spectrometry was obtained on an Agilent 6890N Gas Chromatograph (30 m x 0.25 mm column with 0.25 μm HP-5MS coating, He carrier gas) and Agilent 5973 Mass Spectrometer (Ion source: EI+, 70eV, 230 ° C interface 300 °C).

High-resolution mass spectra were acquired with two methods:

Method 1: an Agilent 7890 Gas Chromatograph coupled with a JEOL JMS-T200GCx time-of-flight mass spectrometer equipped with EI ion source. GC parameters: standard injection (1µl), split ratio: 50, carrier gas: helium; 60 °C initial temperature, 1.5 min hold time, 30 °C/min, final temperature: 320 °C, 3 min hold time; Column: RESTEK RTX-5, 15 m x 0.25 mm x 0.25 µm. MS parameters: Interface temperature: 250 °C, Source temperature: 250 °C, Ionizing voltage: 70eV. The obtained spectra were processed by msAXEL 1.1.6 software.

Method 2: an Agilent 6230 time-of-flight mass spectrometer equipped with a Jet Stream electrospray ion source in positive ion mode. Injections of 0.5µl were directed to the mass spectrometer at a flow rate 1.5 ml/min (5mM ammonium-formate in water and acetonitrile gradient program), using an Agilent 1290 Infinity HPLC system. Jet Stream parameters: drying gas (N_2) flow and temperature: 8.0 l/min and 325 °C, respectively; nebulizer gas (N2) pressure: 30 psi; capillary voltage: 3000 V; sheath gas flow and temperature: 325 °C and 10.0 l/min; TOFMS parameters: fragmentor voltage: 100 V; skimmer potential: 60 V; OCT 1 RF Vpp:750 V. Full-scan mass spectra were acquired over the m/z range 105-1700 at an acquisition rate of 995.6 ms/spectrum and processed by Agilent MassHunter B.04.00 software.

3. Optimization experiments

3.1 Optimization of Pd catalyst loading

The reactions that took place with different catalysts are presented below. In the case of Pd_2dba_3, sampling was carried out after 1, 2, 3, 4, and 18 hours. For the comparison of catalysts, $[(Cinnamyl)PdCl]_2$ was compared with Pd_2dba_3, which also contains a π-system, as well as with $[(Allyl)PdCl]_2$.

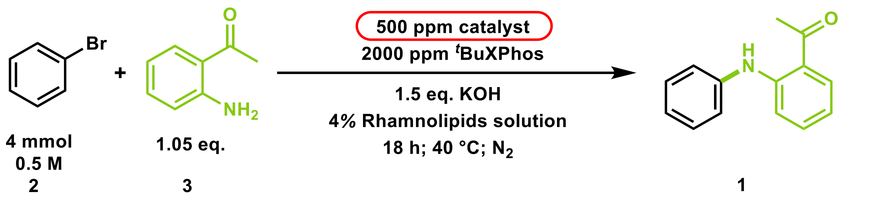

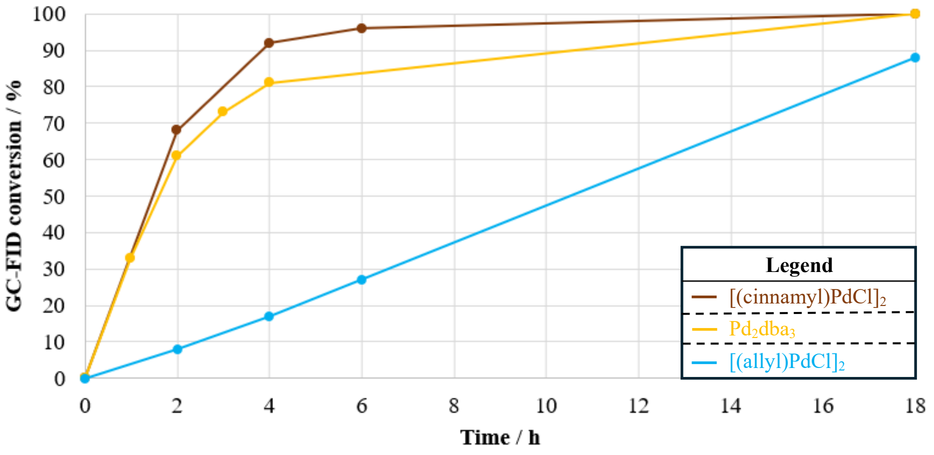

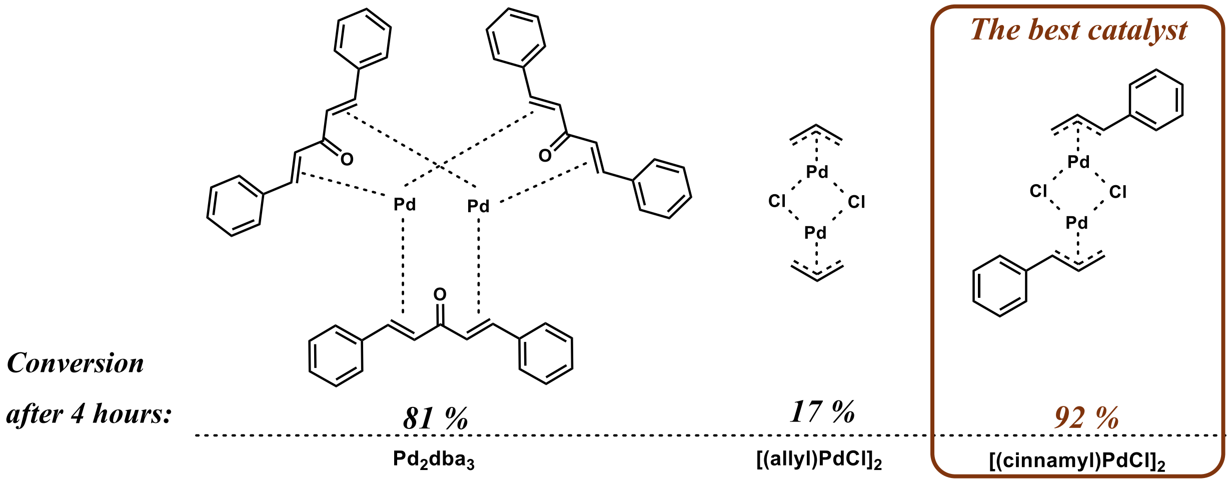

3.2 Ligand screening

Among the ligands listed below (except for ^{t}BuXPhos), only BippyPhos exhibited catalytic activity. What makes the surprising results even more interest is that no product formation was observed even with XPhos, even though structurally, it differs from ^{t}BuXPhos only in the nature of the cycloalkyl groups attached to the phosphorus atom.

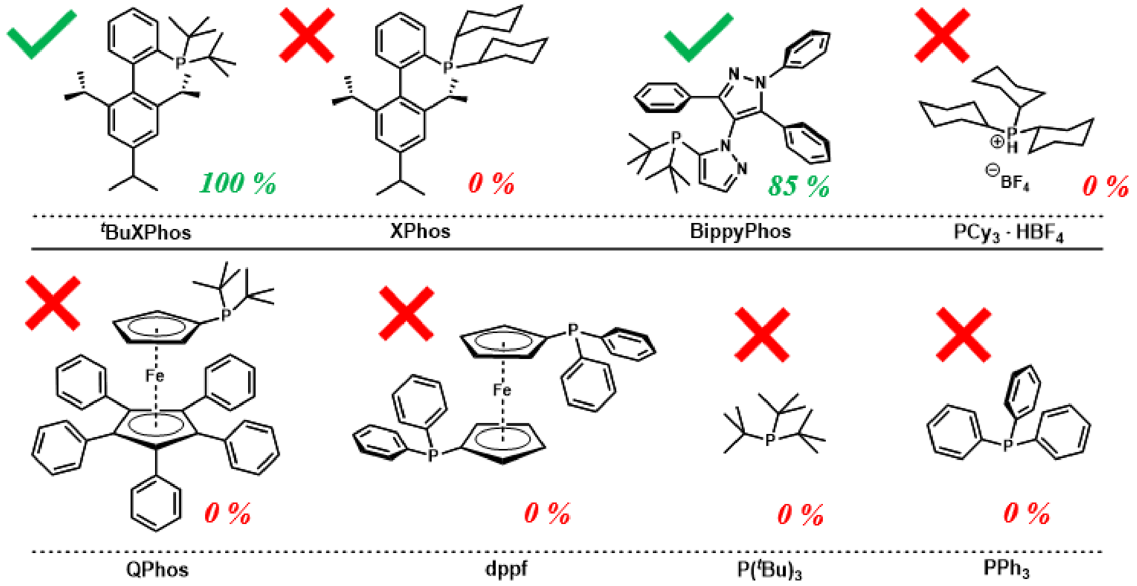

Based on the comparison shown in the figure following this paragraph, BippyPhos formed a less active complex with the catalyst, resulting in a slower reaction compared to when the ^{t}BuXPhos ligand was used.

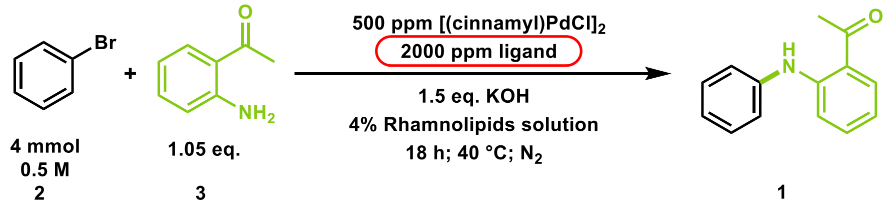

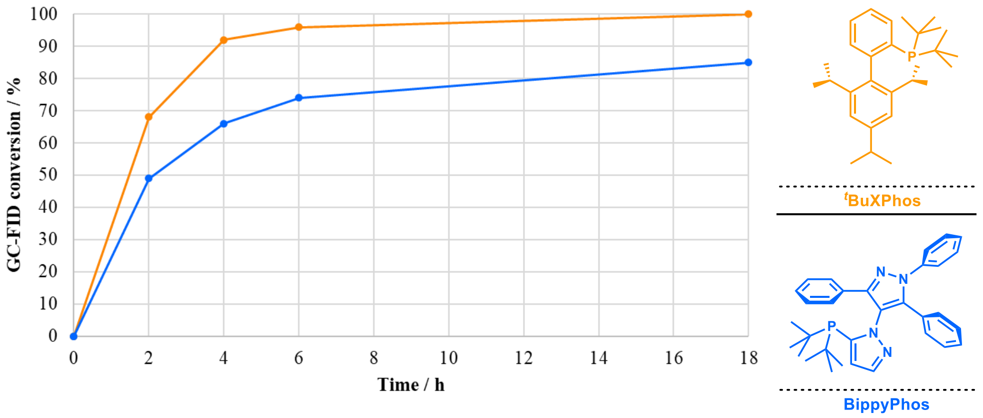

3.3 Optimization of Pd catalyst loading

Different catalyst and ligand loadings were tested. The experiments were carried out under the reaction conditions shown below, using catalyst loadings of 1000 ppm, 500 ppm, 250 ppm, and 50 ppm. The exact quantities used are shown in the table below.

[(cinnamyl)PdCl]$_2$	*^{t}BuXPhos*	*Notes*
1000 ppm	4000 ppm	I measured them in solid form.
500 ppm	2000 ppm	
250 ppm	1000 ppm	
50 ppm	200 ppm	40 µl

Based on the results of the optimization experiments, it can be concluded that the reaction did not go to completion within 18 hours even with a catalyst loading of 250 ppm. As expected, the reaction carried out with the very low catalyst loading of 50 ppm showed only minor conversion. According to the figure below, among the tested catalyst loadings, the highest – 1000 ppm – proved to be the most effective. However, using 500 ppm is also ideal, as the reaction proceeds to completion with only half the amount of catalyst, albeit requiring a longer reaction time. This makes it a much more economical option for scale-up.

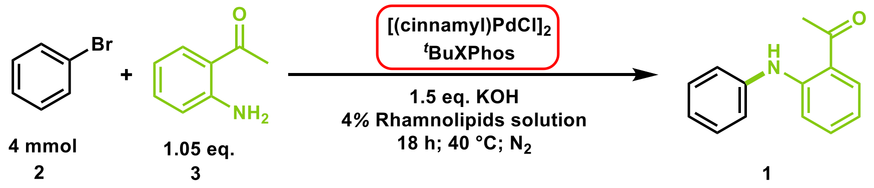

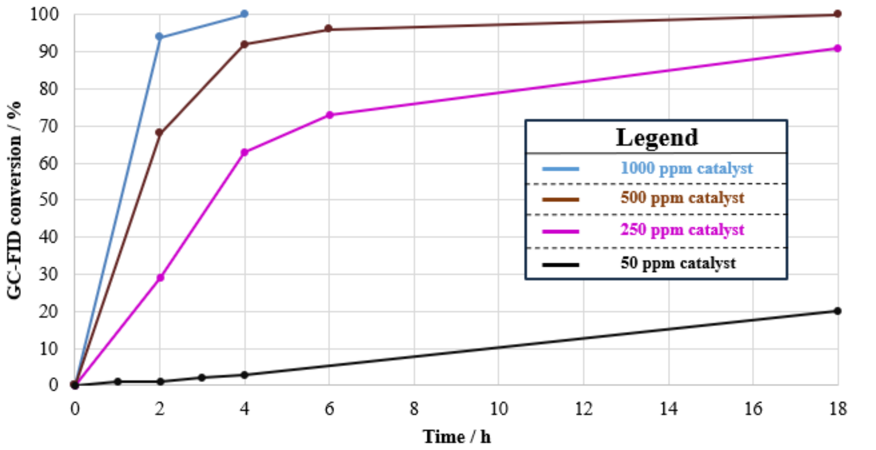

3.4 Effect of Rhamnolipids solution concentration on the reaction

In terms of reaction progress, a significant improvement was observed in the transformation taking place in the 1% Rhamnolipids solution, even compared to the reaction carried out in pure water. The main reason for this is that at a concentration of 1%, rhamnolipid associates were already present, as this concentration was well above the CMC. When comparing the reactions performed in surfactant solutions, only minor differences were observed between the 1 and 2% Rhamnolipids solutions. If the use of a 2% Rhamnolipids solution is sufficient for reaction optimization, it may be worth considering the use of a 1% solution instead, as this can reduce excess surfactant usage and lower additional costs during scale-up. Further analysis of the figure below reveals that the reaction in the 4% solution proceeded more rapidly than in the less concentrated rhamnolipid solutions. Therefore, it is advisable to also test more concentrated solutions during reaction optimization, as this may allow for a reduction in the catalyst loading – often the most expensive component – in cross-coupling reactions, for example. As with the two more dilute solutions, similar reaction profiles were observed with the 4 and 5% Rhamnolipids solutions. Thus, in this case, the surfactant concentration did not significantly influence reactivity either.

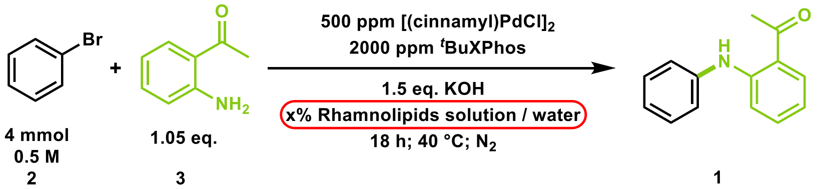

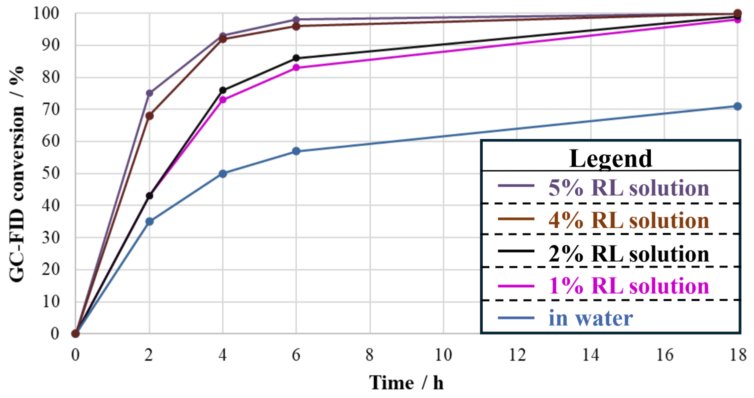

3.5 Comparison of reactions in various surfactant solutions

The conversion curves shown in the figure below indicate that the different surfactant solutions have on significant effect on the reaction outcome. While Kolliphor EL was not the most advantageous for this catalysis, allowing a longer reaction time still led to full conversion. After two hours of reaction time, the catalysis in Tween 80 and TPGS-750-M solution showed slightly better reactivity compared to the Rhamnolipids-solution. However, over longer reaction times all three surfactant solutions resulted in similarly high conversions.

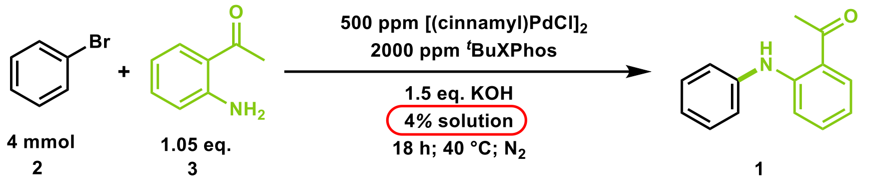

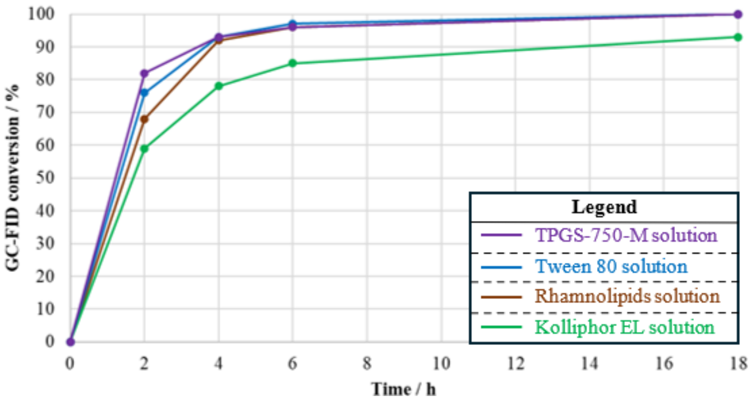

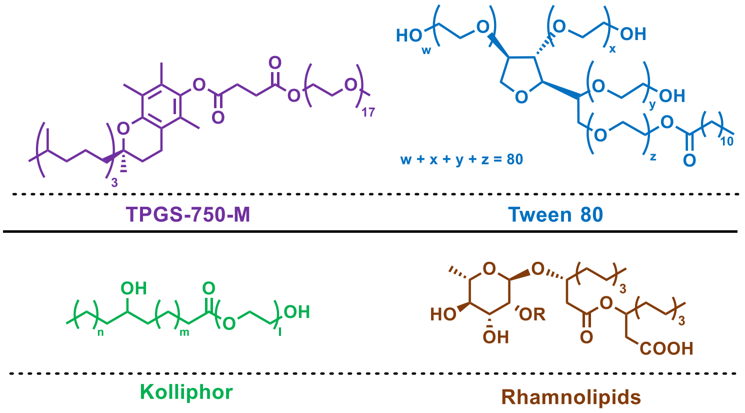

3.6 Comparison with organic solvents

I carried out a comparison of classical solvents that are important in industrial applications. The initial experiments were performed in commonly used cross-coupling solvents such as toluene, dioxane, and *tert*-butanol. To enable a meaningful comparison with the micellar effect of the 4% Rhamnolipids solution, the reaction was also carried out in water. Finally, the transformation was performed under neat conditions, made possible by the fact that both reactants are liquids and thus can serve simultaneously as solvents and substrates. A comparison of the different media is shown below.

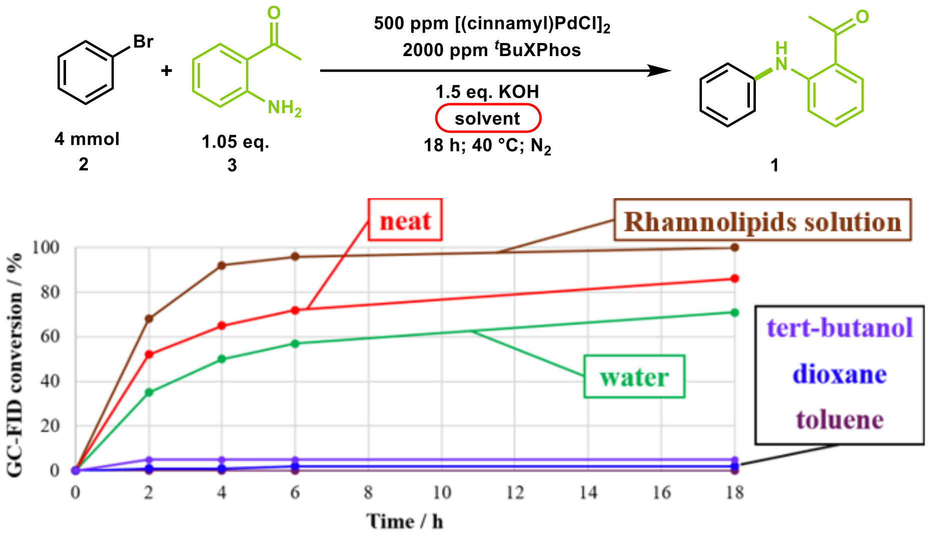

3.7 Comparison of different bases

The model reaction was carried out using KOH, ^{t}BuOK, Na_3PO_4, Na_2CO_3, $NaHCO_3$, and NaOAc as bases. Based on the experiments, KOH was found to be the most suitable base for the amination reaction.

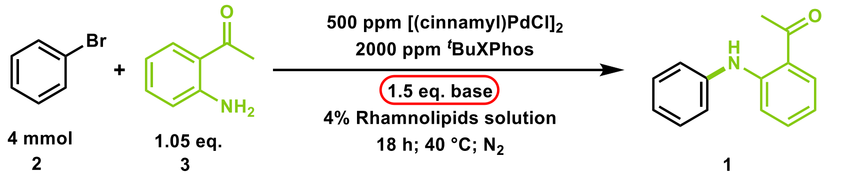

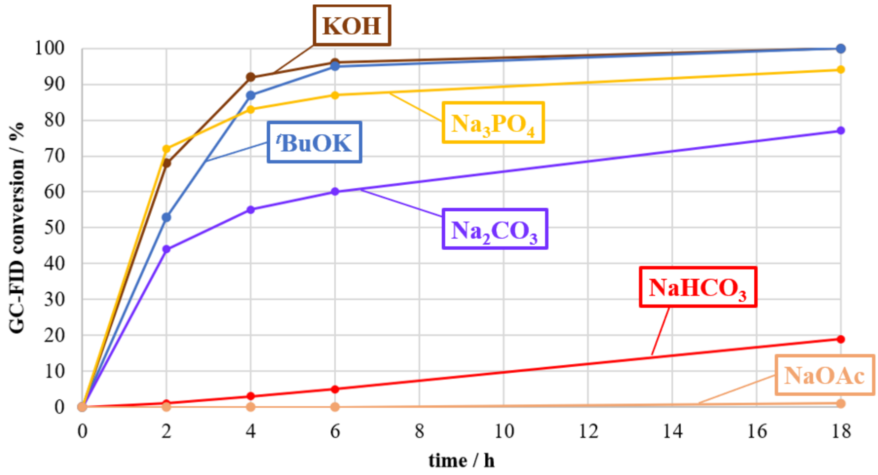

3.8 Temperature screening

The experiments were carried out both at 40 °C and at room temperature. It was found that increasing the temperature facilitates the progress of the reaction and enhances its robustness. The reproducibility of the experiments performed at 40 °C was also greatly improved compared to the reactions conducted at 25 °C.

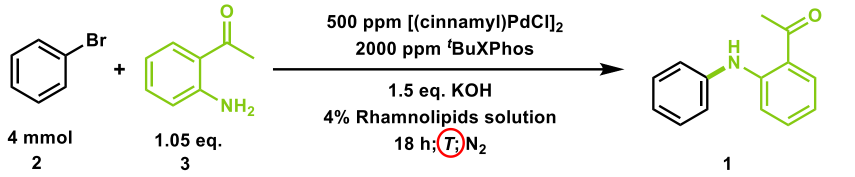

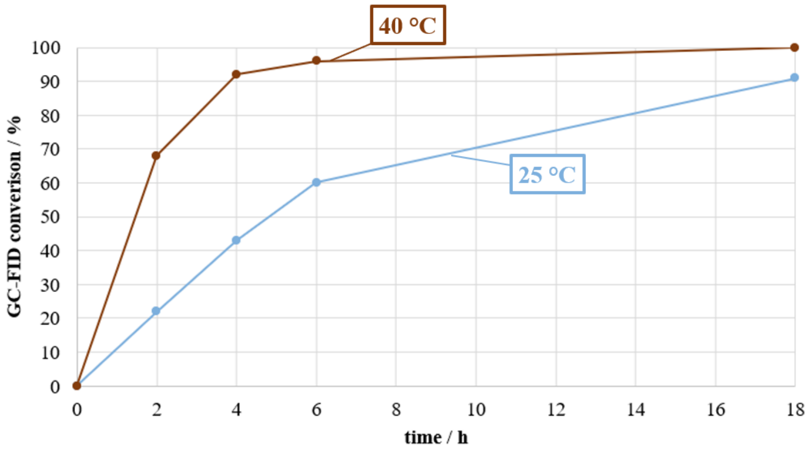

3.9 Catalyst stock-solution in RL

The stock solution was prepared by first weighing out 5.2 mg (0.01 mmol) of [(cinnamyl)PdCl]$_2$, followed by 17 mg (0.04 mmol) of ^{t}BuXPhos. After performing inertization, 2.0 mL of a 4 m/m% rhamnolipid solution was added. Following approximately 2 hours of vigorous stirring, the appearance of a red color indicated complex formation, at which point the solution was added to the reaction mixture. The initial yellow color and the burgundy hue observed upon complex formation are shown in the picture below.

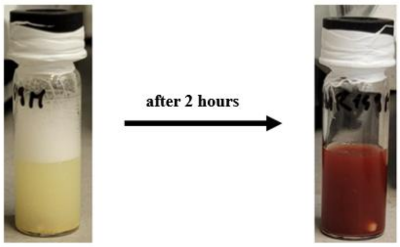

Compared to the solid catalyst, the preformed catalyst complex added after two hours of complexation had a significant impact on the reaction profile. Complete conversion was achieved within two hours.

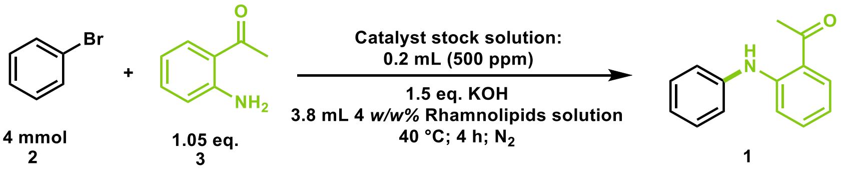

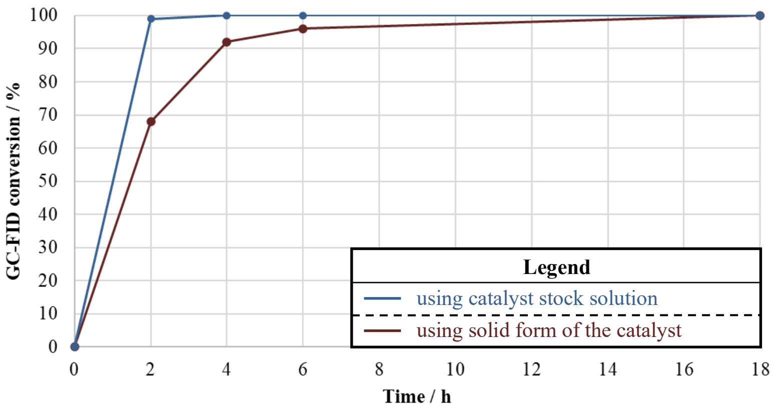

3.10 Comparison of ^{t}BuXPhos-Pd-G3 and catalyst stock solution

The activity of catalyst–ligand complexes that can also be stored in solid form was investigated. As an example, ^{t}BuXPhos-Pd-G3 was selected.

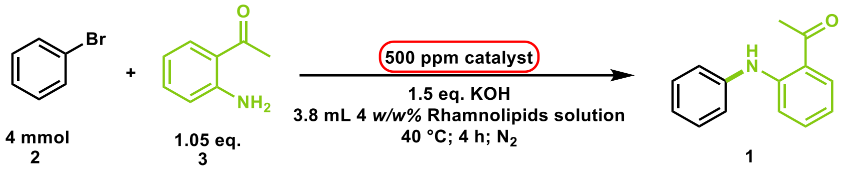

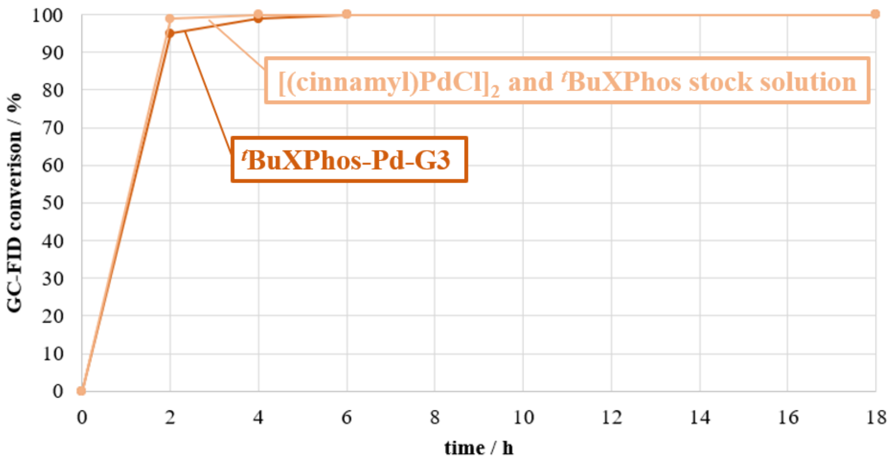

3.11 Substrate scope

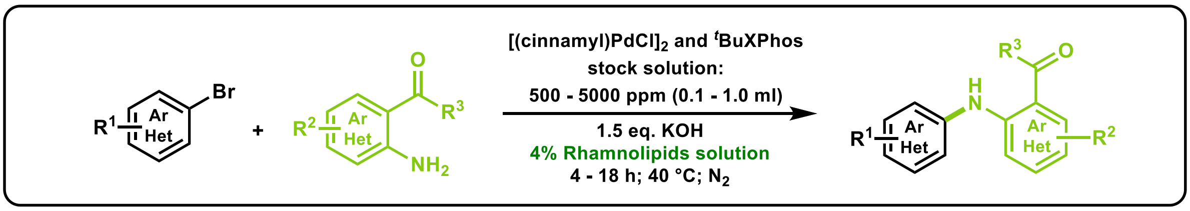

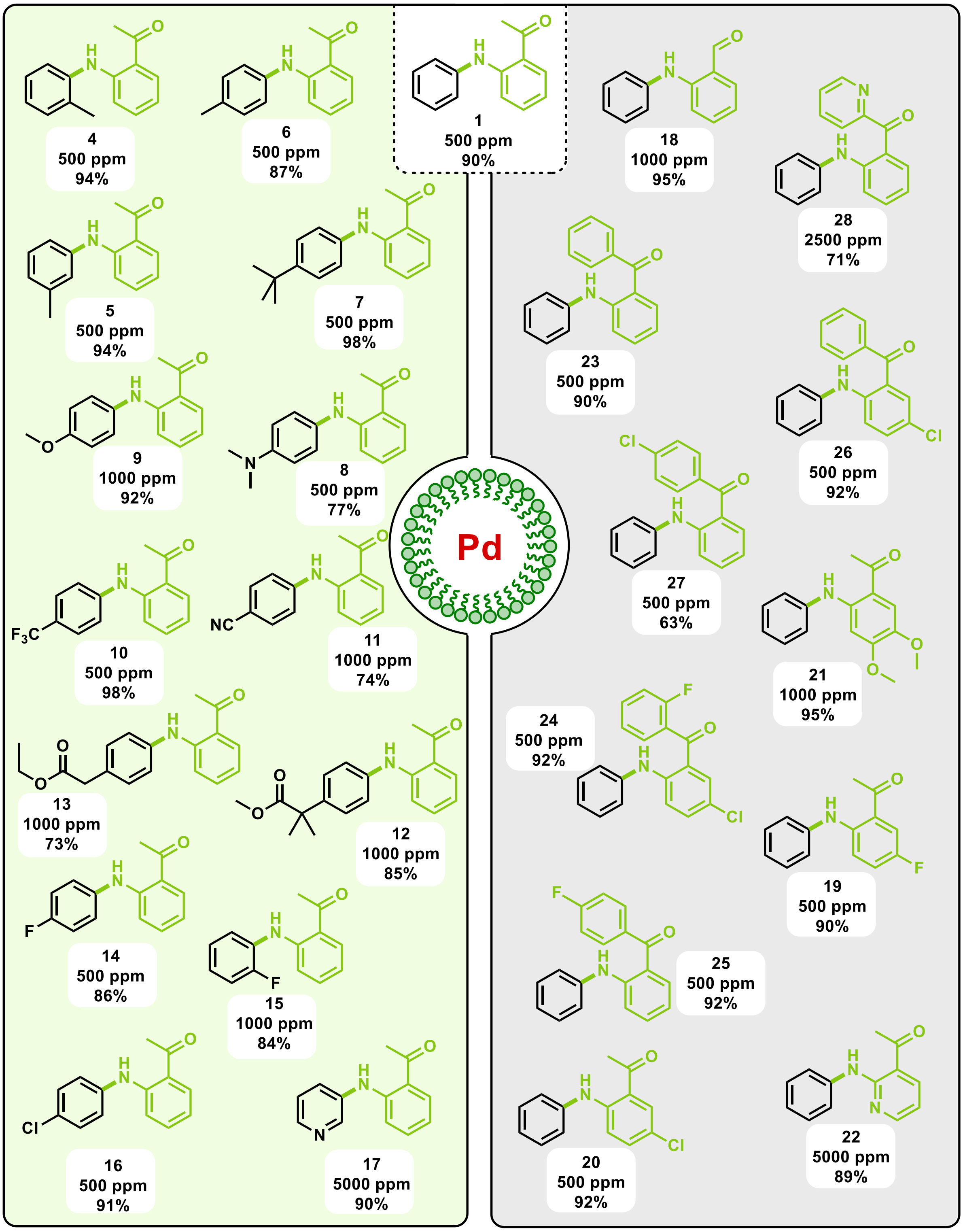

3.12 Green metric analysis[87]

Green metric calculations were also performed for the scaled-up (5 mmol) preparation of product **1**.

**REACTION METRICS FORM**							
**DATE:**	######						
**NAME OF TARGET PRODUCT:**	1-(2-(phenylamino)phenyl)ethan-1-one						
**REACTION CLASSIFICATION:**	B-H cross-coupling reactio						
**BALANCED CHEMICAL EQUATIONS:**							

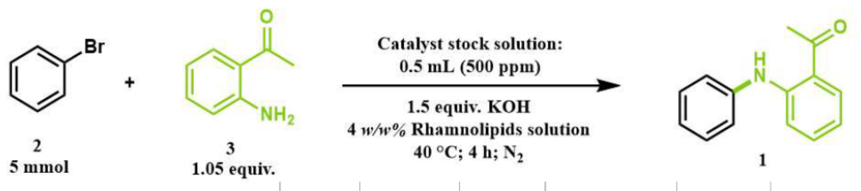

**PART 1: RAW MATERIALS USAGE**							
(A) REACTION STAGE:							
(i) REAGENTS	MW (g/mol)	Density (g/mL)	Volume (mL)	Moles	Mass (g)	Cost ($/g)	Cost ($)
bromobenzene	157	1,49	0,53	0,00499	0,784	4,47	3,504
1-(2-aminophenyl)ethan-1-one	135	1,11	0,64	0,00526	0,71	10,84	7,696
KOH	56,1	-	-	0,00750	0,421	1,35	0,568
TOTAL REAGENTS	348,1				1,915		11,769
(ii) CATALYSTS	MW (g/mol)	Density (g/mL)	Volume (mL)	Moles	Mass (g)	Cost ($/g)	Cost ($)
[(Cinnamyl)PdCl]$_2$	518,08	-	-	0,0000025	0,0013	173	0,225
^{t}BuXPhos	424,65	-	-	0,00001	0,0043	63,1	0,271
TOTAL CATALYSTS					0,0043		0,271
(iii) SOLVENTS	Density (g/mL)	Volume (mL)		Mass (g)	Cost ($/g)	Cost ($)	
Rhamnolipds solution	1,08	10,00		10,8		0,000	19
TOTAL SOLVENTS				10,8		0,000	**20**
**Reaction Materials**				12,7193		12,040	**21**
(B) WORK-UP STAGE:							
MATERIAL	Density (g/mL)	Volume (mL)		Mass (g)	Cost ($/g)	Cost ($)	
EtOAc	0,901	150,00		135,15	0,0828	11,190	22

Na_2CO_3	-	-		5	0,45	2,250	23
TOTAL WORK-UP MATERIALS				140,15		13,440	**24**
(C) PURIFICATION STAGE:							
MATERIAL	Density (g/mL)	Volume (mL)		Mass (g)	Cost ($/g)	Cost ($)	
EtOAc	0,901	47,20		42,5272	0,0828	3,521	25
Hexane	0,659	424,80		279,9432	0,0612	17,133	26
TOTAL PURIFICATION MATERIALS				322,4704		20,654	**27**
**Post-reaction Materials Subtotals**				462,6204		34,094	**28**
				**Mass (g)**	**Cost ($)**		
**TOTAL INPUT MATERIALS**				475,3397	46,135	**29**	
	**MW (g/mol)**	**Moles**	**Yield**	**Mass (g)**	**Cost ($/g)**		
**OUTPUT TARGET PRODUCT**	211,26	0,00	0,9015	0,951	48,512	**30**	
**PART 2: GREEN METRICS ANALYSIS**							
Limiting reagent:							
PARAMETER	VALUE						
Reaction Scale	0,004994	moles		**31**			
E(mw)	0,647733	MW byproducts/ MW product		**32**			
AE	0,606895	MW product/Σ MW reagents		**33**			
(i) Under reclaiming reaction solvents, catalysts, and byproducts, and all post-reaction materials							
Mass of waste	0,964	g		**34**			
E(m)	1,01367	g waste/g product		**35**			
RME	0,496606	g product/Σ g reagents		**36**			
SF	1,101662			**37**			
Wasted input costs ($)	5,924						
(ii) Under committing all reaction solvents, catalysts, and byproducts, and post-reaction materials to waste							
Mass of waste	474,3887	g		**38**			
E(m)	498,8314	g waste/g product		**39**			
RME	0,002001	g product/Σ g reagents		**40**			
Wasted input costs ($)	46,042						
Check formula	0,002001						
(iii) Under reclaiming ...							

Mass of waste		g		**41**
E(m)	0,000	g waste/g product		**42**
RME	1,000	g product/Σ g reagents		**43**
Wasted input costs ($)				

Raw Materials Footprint

Parameter	Complete Reclai	Partial Reclaiming	No Reclaiming	Ideal
AE	0,607	0,607	**0,607**	1
Rxn Yield	0,901	0,901	**0,901**	1
1/SF	0,908	0,908	**0,908**	1
MRP	1	2,014	**0,004**	1
RME	0,497	1,000	**0,002**	1
PMI	499,83	499,8314	**499,83**	1

4. Synthesis

4.1 General preparations and syntheses

4.1.1 Preparation of rhamnolipid solutions

Preparation 100 mL of a 2 *w/w%* Rhamnolipids solution: 4 g of 50 *w/w%* Rhamnolipids solution, and 96 mL of degassed distilled water was added into a 250 mL flask.

This method was used to prepare the 1, 4 and 5 *w/w%* solutions using the measurement data given in ***1. Table***.

Concentration of the solution	Weight of 50 *w/w%* solution (g)	Weight of distilled water (g)
1 *w/w%*	2	98
4 *w/w%*	8	92
5 *w/w%*	10	90

1. Table: Measurement table for solutions of different concentrations for 100 ml of solution.

4.1.2 Preparation of other surfactant solutions

Preparation 10 mL solutions of TPGS-750-M, Brij S100, Kolliphor EL and Tween 80 2 and 4 *w/w%*: 200 or 400 mg of each surfactant and 9.8 or 9.6 mL of degassed distilled water was added into a 16 mL screwcap vial, respectively.

4.1.3 Preparation of catalyst stock solution

A 4 mL reaction vial was charged with $[(Cinnamyl)PdCl]_2$ (0.01 mmol, 5.2 mg) and ^{t}BuXPhos ligand (0.04 mmol, 17.0 mg). A stirring bar was added, then the vial was sealed, evacuated and backfilled with nitrogen three times. In a continuous nitrogen gas flow degassed Rhamnolipids solution (2 mL) was added. The resulting solution has a catalyst complex concentration with 0.005 mmol/mL.

4.1.4 General synthesis of Buchwald-Hartwig amination for 2 mmol product

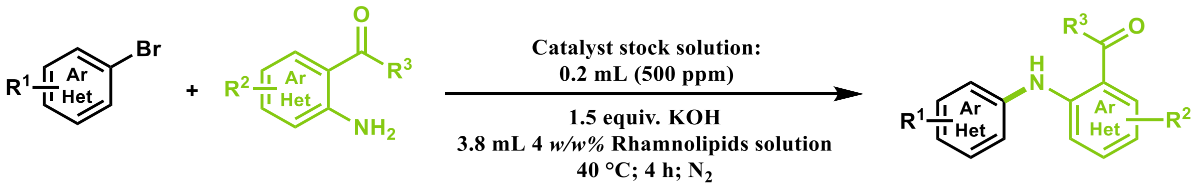

An 8 mL reaction vial was charged with KOH (168 mg, 1.50 equiv., 3 mmol). A stirring bar was added, then the vial was sealed, evacuated and backfilled with nitrogen three times.

Degassed rhamnolipids solution (3.8 mL), aryl- or heteroarylamine (1.05 equiv.), and aryl- or heteroaryl bromide (2 mmol, 1.00 equiv.) were added. Finally, catalyst stock solution (0.2 mL containing [(Cinnamyl)PdCl]$_2$: 0.5 mg, 0.001 mmol, 500 ppm, 0.05 mol%; and ^{t}BuXPhos: 1.7 mg, 0.004 mmol, 2000 ppm, 0.2 mol%) was measured into the mixture. The reaction mixture was vigorously stirred at 40 °C for 4 hours. The reaction was monitored by GC-MS or GC-FID. After the completion, the mixture was allowed to cool down to room temperature and it was diluted with water. The organic phase was separated, and the aqueous phase was extracted with 3x20 ml ethyl acetate. The combined organic phase was dried on Na_2CO_3 and concentrated under vacuo. The product was purified by flash column chromatography.

4.2 Recipes and characterization of Buchwald-Hartwig-products

5 mmol scale reaction of 1-(2-(phenylamino)phenyl)ethan-1-one (1)[88]

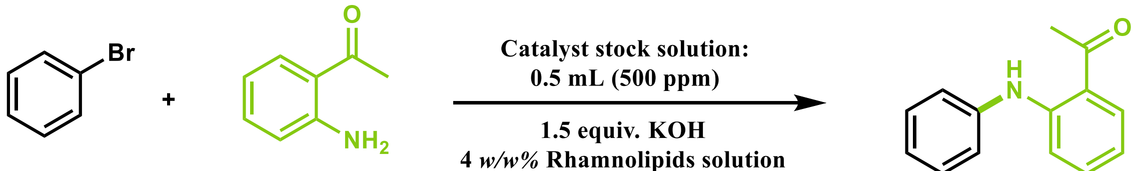

2
5 mmol

3
1.05 equiv.

1

The preparation of the product was carried out in 5 mmol size, and it was based on the general synthesis (see at section **4.1.4**). KOH (1.50 equiv., 7.5 mmol, 421 mg), rhamnolipids-solution (9.5 mL), bromobenzene (1.00 equiv., 5 mmol, 784 mg, 526 µL), 1-(2-aminophenyl)ethan-1-one (1.05 equiv., 5.25 mmol, 710 mg, 638 µL), catalyst stock solution (0.5 mL, amount of [(Cinnamyl)PdCl]$_2$: 1.3 mg, 0.0025 mmol, 500 ppm, 0.05 mol%; amount of ^{t}BuXPhos: 4.3 mg, 0.01 mmol, 2000 ppm, 0.2 mol%) was added. The product was isolated by flash column chromatography using hexane and ethyl acetate 10 : 1 as eluent.

Yield: 951 mg (4.5 mmol, 90%), yellow oil.

R$_f$ = 0.5 (hexane : ethyl acetate = 10 : 1). **^{1}H NMR** (400 MHz, $CDCl_3$) δ 10.56 (bs, 1H), 7.83 (d, J = 1.6 Hz, 1H), 7.41 – 7.26 (m, 6H), 7.17 – 7.09 (m, 1H), 6.79 – 6.71 (m, 1H), 2.66 (s, 3H) ppm. **^{13}C NMR** (101 MHz, $CDCl_3$) δ 201.3, 148.0, 139.5, 134.7, 132.6, 129.5, 124.1, 123.3, 119.7, 116.7, 114.3, 28.3 ppm. **MS** (EI, 70 eV): *m/z* (%): 212(100[M$^+$]), 197(14), 196(99), 168(33), 167(54), 166(16), 120(33).

1-(2-(*o*-tolylamino)phenyl)ethan-1-one (4)[87]

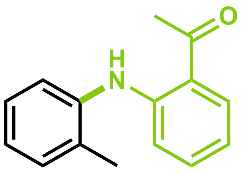

4

The preparation of the product was carried out in 2 mmol size, and it was based on the general synthesis (see at section **4.1.4**). 1-bromo-2-methylbenzene (1.00 equiv., 2 mmol, 342 mg, 241 µL), 1-(2-aminophenyl)ethan-1-one (1.05 equiv., 2.1 mmol, 284 mg, 255 µL), and catalyst stock solution (0.2 mL) were added. The reaction mixture was vigorously stirred at 40 °C for 16 hours.

Yield: 423 mg (1.88 mmol, 94%), yellow solid. Mp= 71 – 73 °C.

R_f = 0.4 (hexane : ethyl acetate = 10 : 1). **^{1}H NMR** (400 MHz, $CDCl_3$) δ 10.41 (s, 1H), 7.85 – 7.78 (m, 1H), 7.36 – 7.31 (m, 1H), 7.31 – 7.25 (m, 2H), 7.24 – 7.18 (m, 1H), 7.14 – 7.08 (m, 1H), 6.95 – 6.88 (m, 1H), 6.73 – 6.66 (m, 1H), 2.67 (s, 3H), 2.29 (s, 3H) ppm. **^{13}C NMR** (101 MHz, $CDCl_3$) δ 201.3, 148.9, 138.7, 134.7, 133.3, 132.6, 131.2, 126.7, 125.0, 124.7, 118.6, 116.0, 114.1, 28.2, 18.2 ppm. **MS** (EI, 70 eV): *m/z* (%): 226(100[M^+]), 210(55), 208(10), 195(14), 182(37), 180(28), 167(28), 120(21).

1-(2-(*m*-tolylamino)phenyl)ethan-1-one (5)

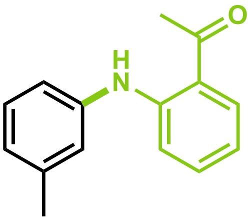

5

The preparation of the product was carried out in 2 mmol size, and it was based on the general synthesis (see at section **4.1.4**). 1-bromo-3-methylbenzene (1.00 equiv., 2 mmol, 342 mg, 243 µL), 1-(2-aminophenyl)ethan-1-one (1.05 equiv., 2.1 mmol, 284 mg, 255 µL), and catalyst stock solution (0.2 mL) were added. The reaction mixture was vigorously stirred at 40 °C for 16 hours.

Yield: 424 mg (1.88 mmol, 94%), yellow oil.

R_f = 0.5 (hexane : ethyl acetate = 10 : 1). **^{1}H NMR** (400 MHz, $CDCl_3$) δ 10.51 (s, 1H), 7.81 (dd, *J* = 8.0, 1.6 Hz, 1H), 7.34 – 7.19 (m, 3H), 7.10 – 7.04 (m, 2H), 6.93 (d, *J* = 7.5 Hz, 1H), 6.72 (ddd, *J* = 8.1, 6.7, 1.4 Hz, 1H), 2.64 (s, 3H), 2.35 (s, 3H) ppm. **^{13}C NMR** (101 MHz, $CDCl_3$) δ 201.2, 148.2, 140.3, 139.4, 134.6, 132.6, 129.2, 124.9, 124.0, 120.3, 119.0, 116.4, 114.4, 28.2, 21.5 ppm. **MS** (EI, 70 eV): *m/z* (%): 226(100[M+]), 210(63), 195(21), 182(27), 180(17), 167(32), 120(30). **HRMS** m/z calcd for $C_{15}H_{16}NO^+$: 226.1226, found: 226.1227.

1-(2-(*p*-tolylamino)phenyl)ethan-1-one (6)[89]

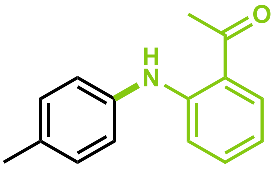

6

The preparation of the product was carried out in 2 mmol size, and it was based on the general synthesis (see at section **4.1.4**). 1-bromo-4-methylbenzene (1.00 equiv., 2 mmol, 342 mg, 246 µL), 1-(2-aminophenyl)ethan-1-one (1.05 equiv., 2.1 mmol, 284 mg, 255 µL), and catalyst stock solution (0.2 mL) were added. The reaction mixture was vigorously stirred at 40 °C for 4 hours.

Yield: 391 mg (1.74 mmol, 87%), yellow solid. Mp= 40 – 45 °C.

R*f* = 0.4 (hexane : ethyl acetate = 10 : 1). **^{1}H NMR** (400 MHz, $CDCl_3$) δ 10.49 (bs, 1H), 7.81 (dd, *J* = 8.1, 1.6 Hz, 1H), 7.33 – 7.26 (m, 1H), 7.21 – 7.13 (m, 5H), 6.73 – 6.67 (m, 1H), 2.65 (s, 3H), 2.36 (s, 3H) ppm. **^{13}C NMR** (101 MHz, $CDCl_3$) δ 201.2, 148.7, 137.6, 134.7, 134.0, 132.6, 130.1, 123.9, 118.7, 116.1, 114.1, 28.2, 21.0 ppm. **MS** (EI, 70 eV): *m/z* (%): 226(100[M^+]), 210(58), 195(20), 182(16), 180(21), 167(29), 120(36).

1-(2-((4-(*tert*-butyl)phenyl)amino)phenyl)ethan-1-one (7)

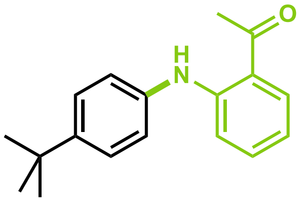

7

The preparation of the product was carried out in 2 mmol size, and it was based on the general synthesis (see at section **4.1.4**). 1-bromo-4-(tert-butyl)benzene (1.00 equiv., 2 mmol, 426 mg, 347 µL), 1-(2-aminophenyl)ethan-1-one (1.05 equiv., 2.1 mmol, 284 mg, 255 µL), and catalyst stock solution (0.2 mL) were added. The reaction mixture was vigorously stirred at 40 °C for 4 hours.

Yield: 525 mg (1.96 mmol, 98%), yellow solid. Mp= 93 – 96 °C.

R*f* = 0.6 (hexane : ethyl acetate = 10 : 1). **^{1}H NMR** (400 MHz, $CDCl_3$) δ 10.51 (s, 1H), 7.84 – 7.77 (m, 1H), 7.43 – 7.35 (m, 2H), 7.32 – 7.25 (m, 1H), 7.24 – 7.17 (m, 3H), 6.74 – 6.66 (m, 1H), 2.64 (s, 3H), 1.34 (s, 9H) ppm. **^{13}C NMR** (101 MHz, $CDCl_3$) δ 201.2, 148.6, 147.2, 137.7, 134.7, 132.6, 126.3, 123.3, 118.8, 116.2, 114.2, 34.5, 31.6, 28.2 ppm. **MS** (EI, 70 eV): *m/z* (%): 268(42[M^+]), 253(19), 252(100). **HRMS** m/z calcd for $C_{18}H_{22}NO^+$: 268.1696, found: 268.1696.

1-(2-((4-(dimethylamino)phenyl)amino)phenyl)ethan-1-one (8)[90]

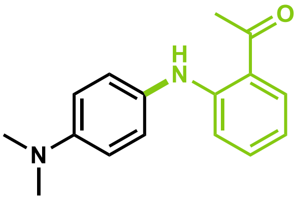

8

The preparation of the product was carried out in 2 mmol size, and it was based on the general synthesis (see at section **4.1.4**). 4-bromo-*N,N*-dimethylaniline (1.00 equiv., 2 mmol, 400 mg, 303 µL), 1-(2-aminophenyl)ethan-1-one (1.05 equiv., 2.1 mmol, 284 mg, 255 µL),

and catalyst stock solution (0.2 mL) were added. The reaction mixture was vigorously stirred at 40 °C for 16 hours.

Yield: 393 mg (1.54 mmol, 77%), yellow solid. Mp= 128 – 130 °C.

R*f* = 0.4 (hexane : ethyl acetate = 3 : 1). **^{1}H NMR** (400 MHz, $CDCl_3$) δ 10.38 (s, 1H), 7.87 – 7.77 (m, 1H), 7.32 – 7.24 (m, 1H), 7.22 – 7.11 (m, 2H), 7.06 – 6.94 (m, 1H), 6.84 – 6.75 (m, 2H), 6.72 – 6.61 (m, 1H), 3.00 (s, 6H), 2.68 (s, 3H) ppm. **^{13}C NMR** (101 MHz, $CDCl_3$) δ 201.0, 150.3, 148.5, 134.6, 132.6, 129.4, 126.5, 117.9, 115.2, 113.9, 113.5, 41.0, 28.1. **MS** (EI, 70 eV): *m/z* (%): 255(100[M^+]), 239(18), 196(10), 167(14), 134(43).

1-(2-((4-methoxyphenyl)amino)phenyl)ethan-1-one (9)[88]

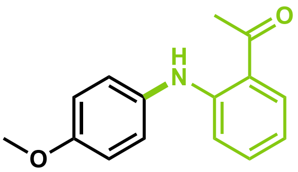

9

The preparation of the product was carried out in 2 mmol size, and it was based on the general synthesis (see at section **4.1.4**). 1-bromo-4-methoxybenzene (1.00 equiv., 2 mmol, 374 mg), 1-(2-aminophenyl)ethan-1-one (1.05 equiv., 2.1 mmol, 284 mg, 255 µL), and catalyst stock solution (0.4 mL) were added. The reaction mixture was vigorously stirred at 40 °C for 16 hours.

Yield: 442 mg (1.83 mmol, 92%), yellow oil.

R*f* = 0.6 (hexane : ethyl acetate = 5 : 1). **^{1}H NMR** (400 MHz, $CDCl_3$) δ 10.38 (s, 1H), 7.79 (dd, *J* = 8.0, 1.1 Hz, 1H), 7.26 (t, *J* = 1.2 Hz, 1H), 7.21 – 7.13 (m, 2H), 6.99 (d, *J* = 8.5 Hz, 1H), 6.95 – 6.87 (m, 2H), 6.71 – 6.62 (m, 1H), 3.82 (s, 3H), 2.64 (s, 3H) ppm. **^{13}C NMR** (101 MHz, $CDCl_3$) δ 201.2, 157.0, 149.6, 134.7, 133.1, 132.6, 126.4, 118.3, 115.7, 114.8, 113.8, 55.6, 28.2 ppm. **MS** (EI, 70 eV): *m/z* (%): 242(100[M+]), 227(11), 226(72), 184(14), 183(14), 180(23), 154(16), 120(16).

1-(2-((4-(trifluoromethyl)phenyl)amino)phenyl)ethan-1-one (10)[88]

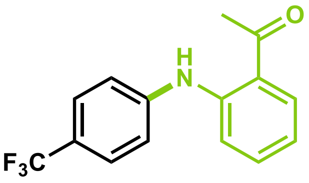

10

The preparation of the product was carried out in 2 mmol size, and it was based on the general synthesis (see at section **4.1.4**). 1-bromo-4-(trifluoromethyl)benzene (1.00 equiv., 2 mmol, 450 mg, 280 µL), 1-(2-aminophenyl)ethan-1-one (1.05 equiv., 2.1 mmol, 284 mg, 255 µL), and catalyst stock solution (0.2 mL) were added. The reaction mixture was vigorously stirred at 40 °C for 16 hours.

Yield: 545 mg (1.96 mmol, 98%), yellow solid. Mp= 74 – 78 °C.

R*f* = 0.5 (hexane : ethyl acetate = 5 : 1). **^{1}H NMR** (400 MHz, $CDCl_3$) δ 10.67 (s, 1H), 7.94 – 7.80 (m, 1H), 7.62 – 7.51 (m, 2H), 7.45 – 7.28 (m, 4H), 6.91 – 6.79 (m, 1H), 2.66 (s, 3H) ppm.

^{13}**C NMR** (101 MHz, CDCl$_3$) δ 201.7, 146.0, 144.2, 132.7, 126.7 (q, *J* = 3.6 Hz), 124.7 (q, *J* = 32.7 Hz), 124.5 (q, *J* = 271.4 Hz), 120.9, 120.5, 118.3, 115.1, 28.4 ppm. ^{19}**F NMR** (376 MHz, CDCl3) δ - 61.9 ppm. **MS** (EI, 70 eV): *m/z* (%): 280(100[M$^+$]), 265(14), 264(93), 236(12), 216(11), 195(24), 167(26), 166(11), 120(29).

4-((2-acetylphenyl)amino)benzonitrile (11)[91]

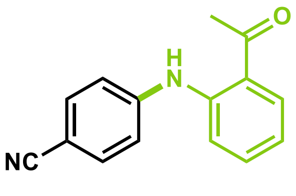

11

The preparation of the product was carried out in 2 mmol size, and it was based on the general synthesis (see at section **4.1.4**). 4-bromobenzonitrile (1.20 equiv., 2.4 mmol, 437 mg, 280 µL), 1-(2-aminophenyl)ethan-1-one (1.00 equiv., 2 mmol, 270 mg, 243 µL), and catalyst stock solution (0.4 mL) were added. The reaction mixture was vigorously stirred at 40 °C for 24 hours.

Yield: 349 mg (1.48 mmol, 74%), yellow oil.

R*f* = 0.4 (hexane : ethyl acetate = 5 : 1). ^{1}**H NMR** (400 MHz, CDCl$_3$) δ 10.66 (s, 1H), 7.86 (d, *J* = 8.0 Hz, 1H), 7.54 (d, *J* = 8.4 Hz, 2H), 7.46 – 7.36 (m, 2H), 7.25 (d, *J* = 8.4 Hz, 2H), 6.91 (tt, *J* = 6.6, 1.3 Hz, 1H), 2.63 (s, 3H) ppm. ^{13}**C NMR** (101 MHz, CDCl$_3$) δ 201.9, 145.4, 144.6, 134.6, 133.7, 132.7, 121.5, 119.8, 119.5, 119.5, 116.0, 28.5 ppm. **MS** (EI, 70 eV): *m/z* (%): 237(90[M+]), 222(16), 221(100), 193(18), 192(30), 120(30).

Methyl 2-(4-((2-acetylphenyl)amino)phenyl)-2-methylpropanoate (12)

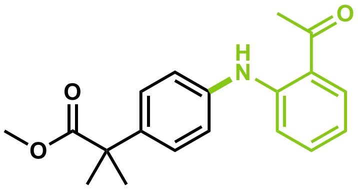

12

The preparation of the product was carried out in 2 mmol size, and it was based on the general synthesis (see at section **4.1.4**). Methyl 2-(4-bromophenyl)-2-methylpropanoate (1.05 equiv., 2.1 mmol, 540 mg, 404 µL), 1-(2-aminophenyl)ethan-1-one (1.00 equiv., 2.0 mmol, 270 mg, 230 µL), and catalyst stock solution (0.4 mL) were added. The reaction mixture was vigorously stirred at 40 °C for 16 hours.

Yield: 527 mg (1.70 mmol, 85%), yellow solid. Mp= 64 – 67 °C.

R*f* = 0.5 (hexane : ethyl acetate = 5 : 1). ^{1}**H NMR** (400 MHz, CDCl$_3$) δ 10.54 (s, 1H), 7.82 (d, *J* = 8.2 Hz, 1H), 7.36 – 7.26 (m, 4H), 7.24 – 7.19 (m, 2H), 6.73 (t, *J* = 7.0 Hz, 1H), 3.68 (s, 3H), 2.64 (s, 3H), 1.60 (s, 6H) ppm. ^{13}**C NMR** (101 MHz, CDCl$_3$) δ 201.3, 177.3, 148.0, 140.2, 139.0, 134.6, 132.6, 126.8, 123.0, 119.1, 116.6, 114.4, 52.4, 46.2, 28.2, 26.7 ppm. **MS** (EI, 70 eV): *m/z* (%): 311(34[M$^+$]), 253(19), 252(100), 234(7). **HRMS** m/z calcd for $C_{19}H_{21}NO_3^+$: 311.1594, found: 311.15220.

Ethyl 2-(4-((2-acetylphenyl)amino)phenyl)acetate (13)

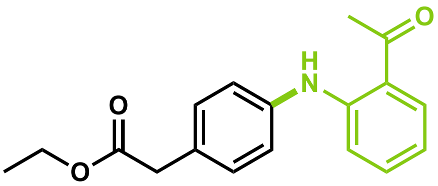

13

The preparation of the product was carried out in 2 mmol size, and it was based on the general synthesis (see at section **4.1.4**). ethyl 2-(4-bromophenyl)acetate (1.00 equiv., 2.00 mmol, 486 mg, 325 µL) and catalyst stock solution (0.4 mL) were added. The reaction mixture was vigorously stirred at 40 °C for 16 hours.

Yield: 434 mg (1.46 mmol, 73%), yellow liquid.

R*f* = 0.4 (hexane : ethyl acetate = 5 : 1). **^{1}H NMR** (400 MHz, $CDCl_3$) δ 10.51 (s, 1H), 7.81 (dd, *J* = 8.1, 1.6 Hz, 1H), 7.35 – 7.17 (m, 6H), 6.72 (ddd, *J* = 8.1, 6.9, 1.4 Hz, 1H), 4.17 (q, *J* = 7.1 Hz, 2H), 3.60 (s, 2H), 2.64 (s, 3H), 1.27 (t, *J* = 7.2 Hz, 3H) ppm. **^{13}C NMR** (101 MHz, $CDCl_3$) δ 201.3, 171.8, 148.0, 139.4, 134.7, 132.6, 130.3, 129.8, 123.4, 119.1, 116.6, 114.3, 61.0, 41.0, 28.3, 14.3 ppm. **MS** (EI, 70 eV): *m/z*(%): 298(49[M^+]), 225(17), 224(100), 180(16), 120(11). **HRMS** m/z calcd for $C_{18}H_{20}NO_3^+$: 298.1438, found: 298.1439.

1-(2-((4-fluorophenyl)amino)phenyl)ethan-1-one (14)[88]

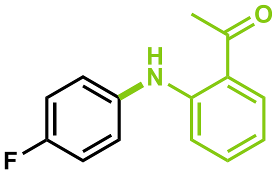

14

The preparation of the product was carried out in 2 mmol size, and it was based on the general synthesis (see at section **4.1.4**). 1-bromo-4-fluorobenzene (1.00 equiv., 2.0 mmol, 350 mg, 220 µL), 1-(2-aminophenyl)ethan-1-one (1.05 equiv., 2.1 mmol, 284 mg, 255 µL), and catalyst stock solution (0.2 mL) were added. The reaction mixture was vigorously stirred at 40 °C for 6 hours.

Yield: 421 mg (1.72 mmol, 86%), yellow solid. Mp= 54 – 57 °C.

R*f* = 0.4 (hexane : ethyl acetate = 10 : 1). **^{1}H NMR** (400 MHz, $CDCl_3$) δ 10.44 (s, 1H), 7.87 – 7.77 (m, 1H), 7.32 – 7.27 (m, 1H), 7.25 – 7.17 (m, 2H), 7.11 – 7.00 (m, 3H), 6.77 – 6.67 (m, 1H), 2.65 (s, 3H) ppm. **^{13}C NMR** (101 MHz, $CDCl_3$) δ 201.4, 159.8 (d, *J* = 243.4 Hz), 148.7, 136.3 (d, *J* = 2.9 Hz), 134.8, 132.7, 125.9 (d, *J* = 8.0 Hz), 118.8, 116.4 (d, *J* = 11.6 Hz), 116.1, 113.8, 28.2 ppm. **^{19}F NMR** (376 MHz, CDC13) δ – 118.2 ppm. **MS** (EI, 70 eV): *m/z* (%): 230(100[M^+]), 215(13), 214(93), 186(23), 185(45), 184(16), 120(37).

1-(2-((2-fluorophenyl)amino)phenyl)ethan-1-one (15)[88]

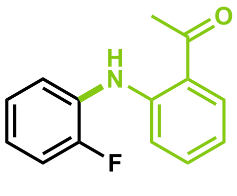

15

The preparation of the product was carried out in 2 mmol size, and it was based on the general synthesis (see at section **4.1.4**). 1-bromo-2-fluorobenzene (1.00 equiv., 2.0 mmol, 350 mg, 219 µL), 1-(2-aminophenyl)ethan-1-one (1.05 equiv., 2.1 mmol, 284 mg, 255 µL), and catalyst stock solution (0.4 mL) were added. The reaction mixture was vigorously stirred at 40 °C for 16 hours.

Yield: 387 mg (1.68 mmol, 84%), yellow liquid.

R*f* = 0.4 (hexane : ethyl acetate = 10 : 1). **^{1}H NMR** (400 MHz, $CDCl_3$) δ 10.45 (s, 1H), 7.84 (dd, *J* = 8.0, 1.3 Hz, 1H), 7.42 (td, *J* = 7.6, 1.9 Hz, 1H), 7.37 – 7.31 (m, 1H), 7.21 – 7.04 (m, 4H), 6.78 (t, *J* = 7.6 Hz, 1H), 2.66 (s, 3H) ppm. **^{13}C NMR** (101 MHz, $CDCl_3$) δ 201.5, 156.3 (d, *J* = 246.7 Hz), 147.4, 134.7, 132.5, 128.4 (d, *J* = 11.6 Hz), 125.0 (d, *J* = 7.6 Hz), 124.7 (d, *J* = 1.8 Hz), 124.4 (d, *J* = 3.6 Hz), 119.6, 117.2, 116.4 (d, *J* = 20.0 Hz), 114.3, 28.2 ppm. **^{19}F NMR** (376 MHz, CDC13) δ – 123.8 ppm. **MS** (EI, 70 eV): *m/z* (%): 230(100[M^+]), 215(11), 214(80), 209(15), 186(10), 185(23), 166(11), 120(19).

1-(2-((4-chlorophenyl)amino)phenyl)ethan-1-one (16)[88]

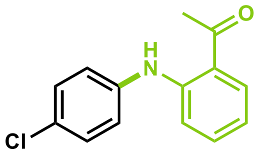

16

The preparation of the product was carried out in 2 mmol size, and it was based on the general synthesis (see at section **4.1.4**). 1-bromo-4-chlorobenzene (1.00 equiv., 2.0 mmol, 383 mg), 1-(2-aminophenyl)ethan-1-one (1.05 equiv., 2.1 mmol, 284 mg, 255 µL), and catalyst stock solution (0.2 mL) were added. The reaction mixture was vigorously stirred at 40 °C for 6 hours.

Yield: 418 mg (1.82 mmol, 91%), yellow solid. Mp= 48 – 52 °C.

R*f* = 0.6 (hexane : ethyl acetate = 5 : 1). **^{1}H NMR** (400 MHz, $CDCl_3$) δ 10.51 (s, 1H), 7.91 – 7.74 (m, 1H), 7.43 – 7.27 (m, 3H), 7.24 – 7.11 (m, 3H), 6.76 (ddd, *J* = 8.1, 7.0, 1.2 Hz, 1H), 2.64 (s, 3H) ppm. **^{13}C NMR** (101 MHz, $CDCl_3$) δ 201.5, 147.6, 139.1, 134.7, 132.7, 129.5, 128.9, 124.3, 119.4, 117.1, 114.2, 28.3 ppm. **MS** (EI, 70 eV): *m/z* (%): 246(100[M^+]), 232(13), 230(41), 195(56), 167(42), 166(23), 139(13), 120(45).

1-(2-(pyridin-3-ylamino)phenyl)ethan-1-one (17)[92]

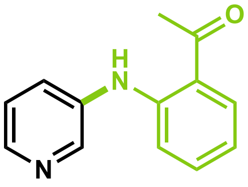

17

The preparation of the product was carried out in 1 mmol size, and it was based on the general synthesis (see at section **4.1.4**). 3-bromopyridine (1.00 equiv., 1.0 mmol, 158 mg), 1-(2-aminophenyl)ethan-1-one (1.05 equiv., 2.1 mmol, 284 mg, 255 µL), and catalyst stock solution (1.0 mL) were added. The reaction mixture was vigorously stirred at 40 °C for 18 hours.

Yield: 193 mg (0.91 mmol, 91%), yellow oil.

R*f* = 0.3 (hexane : ethyl acetate = 1 : 1). **^{1}H NMR** (400 MHz, $CDCl_3$) δ 10.53 (s, 1H), 8.55 (d, *J* = 2.8 Hz, 1H), 8.33 (d, *J* = 4.3 Hz, 1H), 7.83 (dd, *J* = 8.1, 1.6 Hz, 1H), 7.60 – 7.52 (m, 1H), 7.33 (ddd, *J* = 8.3, 6.8, 1.5 Hz, 1H), 7.29 – 7.22 (m, 1H), 7.19 (d, *J* = 8.5 Hz, 1H), 6.79 (ddd, *J* = 8.1, 7.1, 1.1 Hz, 1H), 2.64 (s, 3H) ppm. **^{13}C NMR** (101 MHz, $CDCl_3$) δ 201.7, 147.2, 145.0, 144.9, 137.2, 134.9, 132.7, 129.7, 123.9, 119.8, 117.7, 114.0, 28.3 ppm. **MS** (EI, 70 eV): *m/z*(%): 213(100[M^+]), 211(11), 198(10), 197(71), 169(38), 168(24), 120(20), 115(15).

2-(phenylamino)benzaldehyde (18)[88]

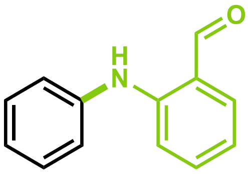

18

The preparation of the product was carried out in 2 mmol size, and it was based on the general synthesis (see at section **4.1.4**). Before the inertization 2-aminobenzaldehyde (1.50 equiv., 3 mmol, 363 mg) was added. Bromobenzene (1.00 equiv., 2 mmol, 314 mg, 211 µL), and catalyst stock solution (0.4 mL) were measured into the mixture. The reaction mixture was vigorously stirred at 40 °C for 16 hours.

Yield: 373 mg (1.90 mmol, 95%), yellow solid. Mp= 74 – 77 °C.

R*f* = 0.6 (hexane : ethyl acetate = 10 : 1). **^{1}H NMR** (400 MHz, $CDCl_3$) δ 10.03 (s, 1H), 9.91 (s, 1H), 7.62 – 7.52 (m, 1H), 7.37 (q, *J* = 7.8 Hz, 3H), 7.29 (d, *J* = 7.5 Hz, 2H), 7.24 (d, *J* = 8.6 Hz, 1H), 7.16 (t, *J* = 7.4 Hz, 1H), 6.83 (t, *J* = 7.4 Hz, 1H) ppm. **^{13}C NMR** (101 MHz, $CDCl_3$) δ 194.4, 147.9, 139.8, 136.7, 135.6, 129.5, 124.5, 123.3, 119.5, 117.2, 113.0 ppm. **MS** (EI, 70 eV): *m/z*(%): 198(56[M^+]), 196(22), 179(11), 169(16), 168(100), 167(37).

1-(5-fluoro-2-(phenylamino)phenyl)ethan-1-one (19)

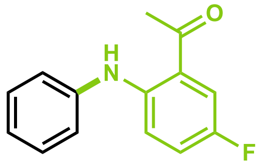

19

The preparation of the product was carried out in 1 mmol size, and it was based on the general synthesis (see at section **4.1.4**). 1-(2-amino-5-fluorophenyl)ethan-1-one (1.05 equiv., 1.05 mmol, 161 mg) and catalyst stock solution (0.1 mL) were added. The reaction mixture was vigorously stirred at 40 °C for 4 hours.

Yield: 207 mg (0.90 mmol, 90%), yellow solid. Mp= 58 – 62 °C.

R*f* = 0.6 (hexane : ethyl acetate = 5 : 1). **^{1}H NMR** (400 MHz, $CDCl_3$) δ 10.30 (s, 1H), 7.48 (dd, *J* = 9.8, 3.0 Hz, 1H),7.38 – 7.32 (m, 2H), 7.26 – 7.19 (m, 3H), 7.14 – 7.04 (m, 2H), 2.62 (s, 3H) ppm. **^{13}C NMR** (101 MHz, $CDCl_3$) δ 200.3 (d, *J* = 2.7 Hz), 153.8 (d, *J* = 236.4 Hz), 144.6, 140.6, 129.6, 124.0, 122.9, 122.4 (d. *J* = 23.1 Hz), 119.0 (d, *J* = 5.2 Hz), 117.3 (d, *J* = 22.1 Hz), 116.0 (d, *J* = 6.9 Hz), 28.3 ppm. **^{19}F NMR** (376 MHz, $CDCl_3$) δ – 127.0 ppm. **MS** (EI, 70 eV): *m/z*(%): 230(100[M^+]), 214(67), 186(32), 185(43), 138(30), 184(15). **HRMS** m/z calcd for $C_{14}H_{13}FNO^+$: 230.0976, found 230.0976.

1-(5-chloro-2-(phenylamino)phenyl)ethan-1-one (20)

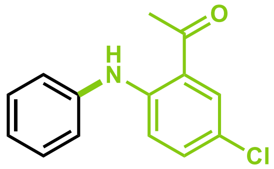

20

The preparation of the product was carried out in 2 mmol size, and it was based on the general synthesis (see at section **4.1.4**). 1-(2-amino-5-chlorophenyl)ethan-1-one (1.05 equiv., 2.10 mmol, 356 mg) and catalyst stock solution (0.2 mL) were added. The reaction mixture was vigorously stirred at 40 °C for 16 hours.

Yield: 452 mg (1.84 mmol, 92%), yellow oil.

R*f* = 0.7 (hexane : ethyl acetate = 5 : 1). **^{1}H NMR** (400 MHz, $CDCl_3$) δ 10.47 (s, 1H), 7.75 (d, *J* = 2.5 Hz, 1H), 7.40 – 7.30 (m, 2H), 7.26 – 7.06 (m, 5H), 2.63 (s, 3H) ppm. **^{13}C NMR** (101 MHz, $CDCl_3$) δ 200.3, 146.7, 139.9, 134.5, 131.7, 129.6, 124.5, 123.4, 120.8, 119.7, 115.9, 28.2 ppm. **MS** (EI, 70 eV): *m/z*(%): 246(100[M^+]), 232(10), 230(30), 195(27), 167(21), 166(10), 154(11). **HRMS** m/z calcd for $C_{14}H_{13}ClNO^+$: 246.0680, found 246.0680.

1-(4,5-dimethoxy-2-(phenylamino)phenyl)ethan-1-one (21)[88]

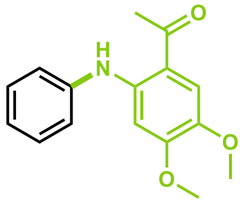

21

The preparation of the product was carried out in 2 mmol size, and it was based on the general synthesis (see at section **4.1.4**). 1-(2-amino-4,5-dimethoxyphenyl)ethan-1-one (1.05 equiv., 2.10 mmol, 411 mg) and catalyst stock solution (0.4 mL) were added. The reaction mixture was vigorously stirred at 40 °C for 16 hours.

Yield: 518 mg (1.90 mmol, 95%), yellow solid. Mp= 90 – 92 °C.

R*f* = 0.5 (hexane : ethyl acetate = 2 : 1). **^{1}H NMR** (400 MHz, $CDCl_3$) δ 10.70 (s, 1H), 7.35 (t, *J* = 7.9 Hz, 2H), 7.26 (d, *J* = 7.3 Hz, 2H), 7.21 (s, 1H), 7.09 (t, *J* = 7.3 Hz, 1H), 6.78 (s, 1H), 3.87 (s, 3H), 3.79 (s, 3H), 2.59 (s, 3H) ppm. **^{13}C NMR** (101 MHz, $CDCl_3$) δ 198.8, 155.4, 145.2, 140.9, 140.5, 129.5, 123.7, 122.7, 114.7, 111.7, 96.9, 56.9, 55.9, 28.1 ppm. **MS** (EI, 70 eV): *m/z*(%): 272(93[M^+]), 257(17), 256(100), 228(10).

1-(2-(phenylamino)pyridin-3-yl)ethan-1-one (22)

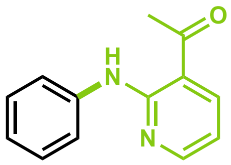

22

The preparation of the product was carried out in 1 mmol size, and it was based on the general synthesis (see at section **4.1.4**). 1-(2-aminopyridin-3-yl)ethan-1-one (1.05 equiv., 1.05 mmol, 143 mg) and catalyst stock solution (1.0 mL) were added. The reaction mixture was vigorously stirred at 40 °C for 18 hours.

Yield: 190 mg (0.89 mmol, 89%), yellow solid. Mp= 70 – 75 °C.

R*f* = 0.5 (hexane : ethyl acetate = 5 : 1). **^{1}H NMR** (400 MHz, $CDCl_3$) δ 11.10 (s, 1H), 8.38 (dd, *J* = 4.8, 2.0 Hz, 1H), 8.08 (dd, *J* = 7.9, 2.0 Hz, 1H), 7.72 (d, *J* = 8.6 Hz, 2H), 7.35 (t, *J* = 8.0 Hz, 2H), 7.07 (t, *J* = 7.4 Hz, 1H), 6.73 (dd, *J* = 7.8, 4.7 Hz, 1H), 2.63 (s, 3H) ppm. **^{13}C NMR** (101 MHz, $CDCl_3$) δ 200.2, 155.6, 154.0, 141.0, 139.6, 128.9, 123.3, 121.6, 113.8, 113.0, 27.5 ppm. **MS** (EI, 70 eV): *m/z*(%): 213(100[M^+]), 193(16), 169(15), 168(13), 77(10). **HRMS** m/z calcd for $C_{13}H_{13}N_2O^+$: 213.1022, found 213.1023.

Phenyl(2-(phenylamino)phenyl)methanone (23)[88]

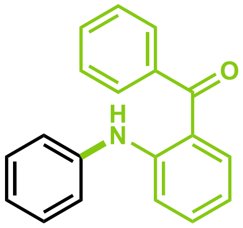

23

The preparation of the product was carried out in 2 mmol size, and it was based on the general synthesis (see at section **4.1.4**). (2-aminophenyl)(phenyl)methanone (1.05 equiv., 2.10 mmol, 414 mg) and catalyst stock solution (0.2 mL) were added. The reaction mixture was vigorously stirred at 40 °C for 6 hours.

Yield: 493 mg (1.80 mmol, 90%), orange oil.

R*f* = 0.5 (hexane : ethyl acetate = 10 : 1). **^{1}H NMR** (400 MHz, $CDCl_3$) δ 10.14 (s, 1H), 7.72 (dd, *J* = 8.3, 1.3 Hz, 2H), 7.59 – 7.54 (m, 2H), 7.49 (t, *J* = 7.3 Hz, 2H), 7.41 – 7.29 (m, 6H), 7.11 (t, *J* = 7.3 Hz, 1H), 6.72 (t, *J* = 1.4 Hz, 1H) ppm. **^{13}C NMR** (101 MHz, $CDCl_3$) δ 199.3, 148.2, 140.7, 139.9, 135.1, 134.3, 131.5, 129.5, 128.3, 123.7, 122.3, 119.9, 116.7, 114.8 ppm. **MS** (EI, 70 eV): *m/z*(%): 274(63[M^+]), 272(100), 167(10).

(5-chloro-2-(phenylamino)phenyl)(2-fluorophenyl)methanone (24)[93]

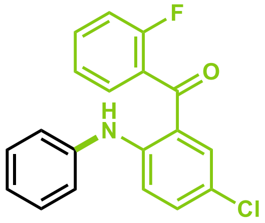

24

The preparation of the product was carried out in 2 mmol size, and it was based on the general synthesis (see at section **4.1.4**). (2-amino-5-chlorophenyl)(2-fluorophenyl)methanone (1.05 equiv., 2.10 mmol, 524 mg) and catalyst stock solution (0.2 mL) were added. The reaction mixture was vigorously stirred at 40 °C for 16 hours.

Yield: 597 mg (1.84 mmol, 92%), orange oil.

R*f* = 0.6 (hexane : ethyl acetate = 10 : 1). **^{1}H NMR** (400 MHz, $CDCl_3$) δ 10.48 (s, 1H), 7.59 – 7.48 (m, 2H), 7.47 – 7.39 (m, 3H), 7.36 – 7.28 (m, 5H), 7.25 – 7.17 (m, 2H) ppm. **^{13}C NMR** (101 MHz, $CDCl_3$) δ 194.8, 159.3 (d, *J* = 250.7 Hz), 147.6, 139.7, 135.3, 133.8 (d, *J* = 1.8 Hz), 132.6 (d, *J* = 8.4 Hz), 130.0 (d, *J* = 2.9 Hz), 129.7, 127.9 (d, *J* = 16.0 Hz), 124.8, 124.5 (d, *J* = 3.6 Hz), 123.5, 121.0, 119.9, 116.5 (d, *J* = 21.4 Hz), 115.9. **^{19}F NMR** (376 MHz, $CDCl_3$) δ – 112.9 ppm. **MS** (EI, 70 eV): *m/z*(%): 326(100[M^+]), 324(85), 308(24), 307(14), 306(36), 305(19), 195(10), 167(16), 166(13), 123(14), 95(11).

(4-fluorophenyl)(2-(phenylamino)phenyl)methanone (25)

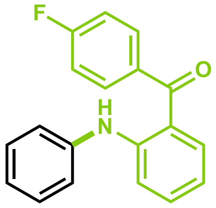

25

The preparation of the product was carried out in 2 mmol size, and it was based on the general synthesis (see at section **4.1.4**). (2-aminophenyl)(4-fluorophenyl)methanone (1.05 equiv., 2.10 mmol, 452 mg) and catalyst stock solution (0.2 mL) were added. The reaction mixture was vigorously stirred at 40 °C for 16 hours.

Yield: 426 mg (1.46 mmol, 73%), yellow oil.

R*f* = 0.6 (hexane : ethyl acetate = 5 : 1). **^{1}H NMR** (400 MHz, $CDCl_3$) δ 9.86 (s, 1H), 7.67 – 7.60 (m, 2H), 7.39 (d, *J* = 8.0 Hz, 1H), 7.30 – 7.20 (m, 4H), 7.20 – 7.13 (m, 2H), 7.05 (t, *J* = 8.5 Hz, 2H), 7.01 – 6.95 (m, 1H), 6.67 – 6.56 (m, 1H) ppm. **^{13}C NMR** (101 MHz, $CDCl_3$) δ 197.7, 166.1, 163.6, 148.1, 140.6, 136.0 (d, *J* = 3.2 Hz), 134.7, 134.4, 132.1 (d, *J* = 9.0 Hz), 129.5, 123.7, 122.2, 119.9, 116.8, 115.5, 115.3, 115.0 ppm. **^{19}F NMR** (376 MHz, $CDCl_3$) δ – 107.59 ppm. **MS** (EI, 70 eV): *m/z*(%): 292(13[M+]), 291(71), 290(100), 167(20), 123(11), 95(16). **HRMS** m/z calcd for $C_{19}H_{15}FNO^+$: 292.1132, found 292.1133.

(5-chloro-2-(phenylamino)phenyl)(phenyl)methanone (26)[88]

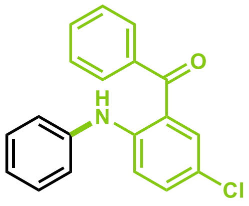

26

The preparation of the product was carried out in 2 mmol size, and it was based on the general synthesis (see at section **4.1.4**). (2-amino-5-chlorophenyl)(phenyl)methanone (1.05 equiv., 2.10 mmol, 487 mg) and catalyst stock solution (0.2 mL) were added. The reaction mixture was vigorously stirred at 40 °C for 16 hours.

Yield: 565 mg (1.84 mmol, 92%), orange oil.

R*f* = 0.6 (hexane : ethyl acetate = 10 : 1). **^{1}H NMR** (400 MHz, $CDCl_3$) δ 9.94 (s, 1H), 7.68 – 7.62 (m, 2H), 7.55 – 7.49 (m, 1H), 7.48 – 7.41 (m, 3H), 7.31 (t, *J* = 7.9 Hz, 2H), 7.25 – 7.16 (m, 4H), 7.10 – 7.03 (m, 1H) ppm. **^{13}C NMR** (101 MHz, $CDCl_3$) δ 198.1, 146.8, 140.2, 139.2, 134.2, 133.8, 132.0, 129.6, 129.5, 128.5, 124.1, 122.5, 121.2, 120.7, 116.4 ppm. **MS** (EI, 70 eV): *m/z*(%): 308(64[M^+]), 306(100), 167(10), 77(12).

(4-chlorophenyl)(2-(phenylamino)phenyl)methanone (27)[92]

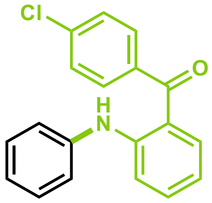

27

The preparation of the product was carried out in 2 mmol size, and it was based on the general synthesis (see at section **4.1.4**). (2-aminophenyl)(4-chlorophenyl)methanone (1.05 equiv., 2.10 mmol, 487 mg) and catalyst stock solution (0.2 mL) were added. The reaction mixture was vigorously stirred at 40 °C for 18 hours.

Yield: 387 mg (1.26 mmol, 63%), yellow solid. Mp= 83 – 86 °C.

R*f* = 0.6 (hexane : ethyl acetate = 10 : 1). **^{1}H NMR** (400 MHz, $CDCl_3$) δ 10.09 (s, 1H), 7.71 – 7.64 (m, 2H), 7.53 – 7.44 (m, 3H), 7.41 – 7.28 (m, 6H), 7.12 (t, *J* = 7.3 Hz, 1H), 6.72 (t, *J* = 7.3 Hz, 1H) ppm. **^{13}C NMR** (101 MHz, $CDCl_3$) δ 197.8, 148.2, 140.5, 138.2, 137.8, 134.7, 134.6, 131.0, 129.5, 128.6, 123.8, 122.4, 119.5, 116.7, 114.9 ppm. **MS** (EI, 70 eV): *m/z*(%): 308(69[M^+]), 306(100), 272(17), 167(17).

(2-(phenylamino)phenyl)(pyridine-2-yl)methanone (28)

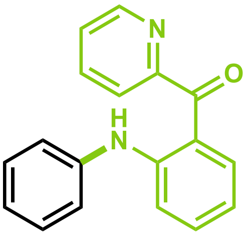

28

The preparation of the product was carried out in 1 mmol size, and it was based on the general synthesis (see at section **4.1.4**). (2-aminophenyl)(pyridin-2-yl)methanone (1.05 equiv., 1.05 mmol, 208 mg) and catalyst stock solution (0.5 mL) were added. The reaction mixture was vigorously stirred at 40 °C for 18 hours.

Yield: 195 mg (0.71 mmol, 71%), orange oil.

R*f* = 0.6 (hexane : ethyl acetate = 2 : 1). **^{1}H NMR** (400 MHz, $CDCl_3$) δ 10.33 (s, 1H), 8.72 (d, *J* = 4.9 Hz, 1H), 7.93 – 7.80 (m, 2H), 7.73 (d, *J* = 8.1 Hz, 1H), 7.44 (ddd, *J* = 6.5, 4.8, 1.6 Hz, 1H), 7.39 – 7.29 (m, 6H), 7.12 (t, *J* = 7.2 Hz, 1H), 6.71 (m, *J* = 4.1 Hz, 1H) ppm. **^{13}C NMR** (101 MHz, $CDCl_3$) δ 196.3, 157.3, 149.2, 148.6, 140.4, 137.1, 135.6, 134.9, 129.5, 125.3, 124.2, 124.0, 122.9, 118.3, 116.7, 114.5 ppm. **MS** (EI, 70 eV): *m/z*(%): 275(14[M^+]), 246(21), 245(100), 169(10), 167(20). **HRMS** m/z calcd for $C_{18}H_{15}N_2O^+$: 275.1179, found: 275.1180.

5. NMR Spectra

1-(2-(phenylamino)phenyl)ethan-1-one (1)

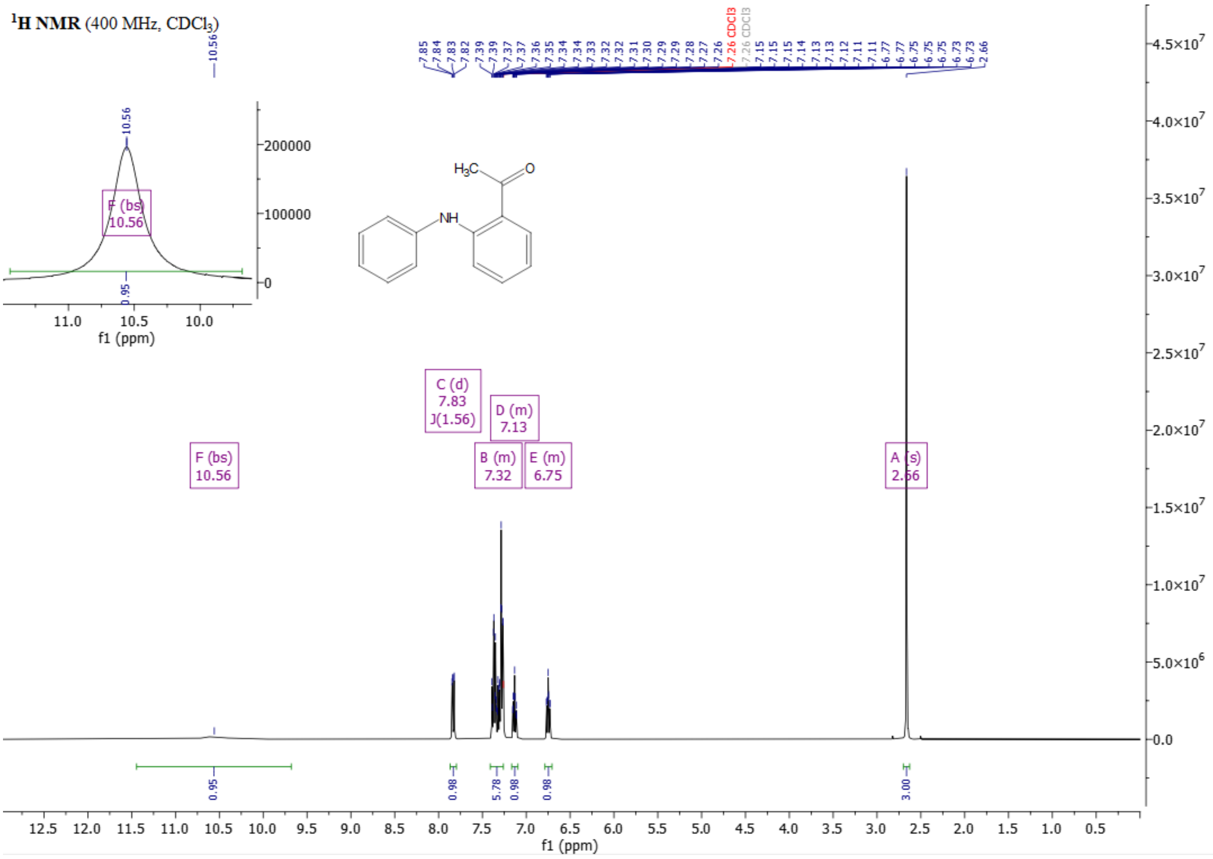

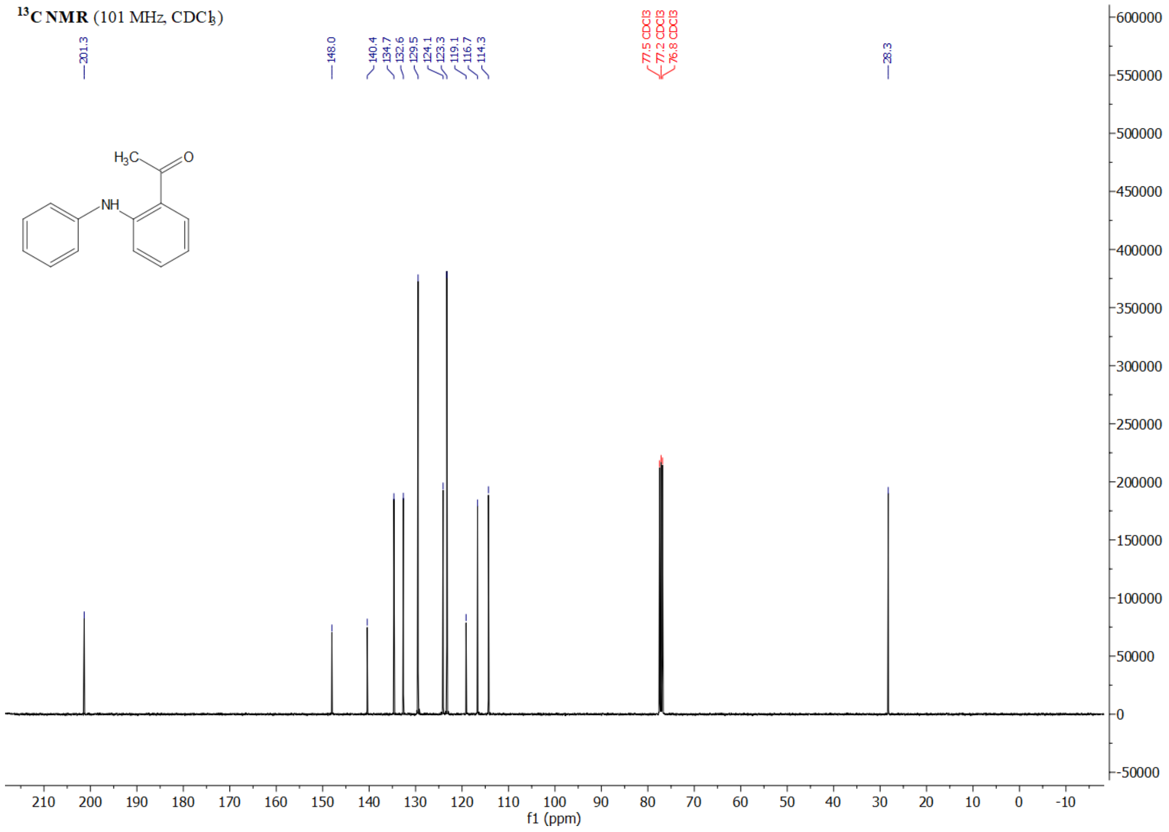

1-(2-(*o*-tolylamino)phenyl)ethan-1-one (4)

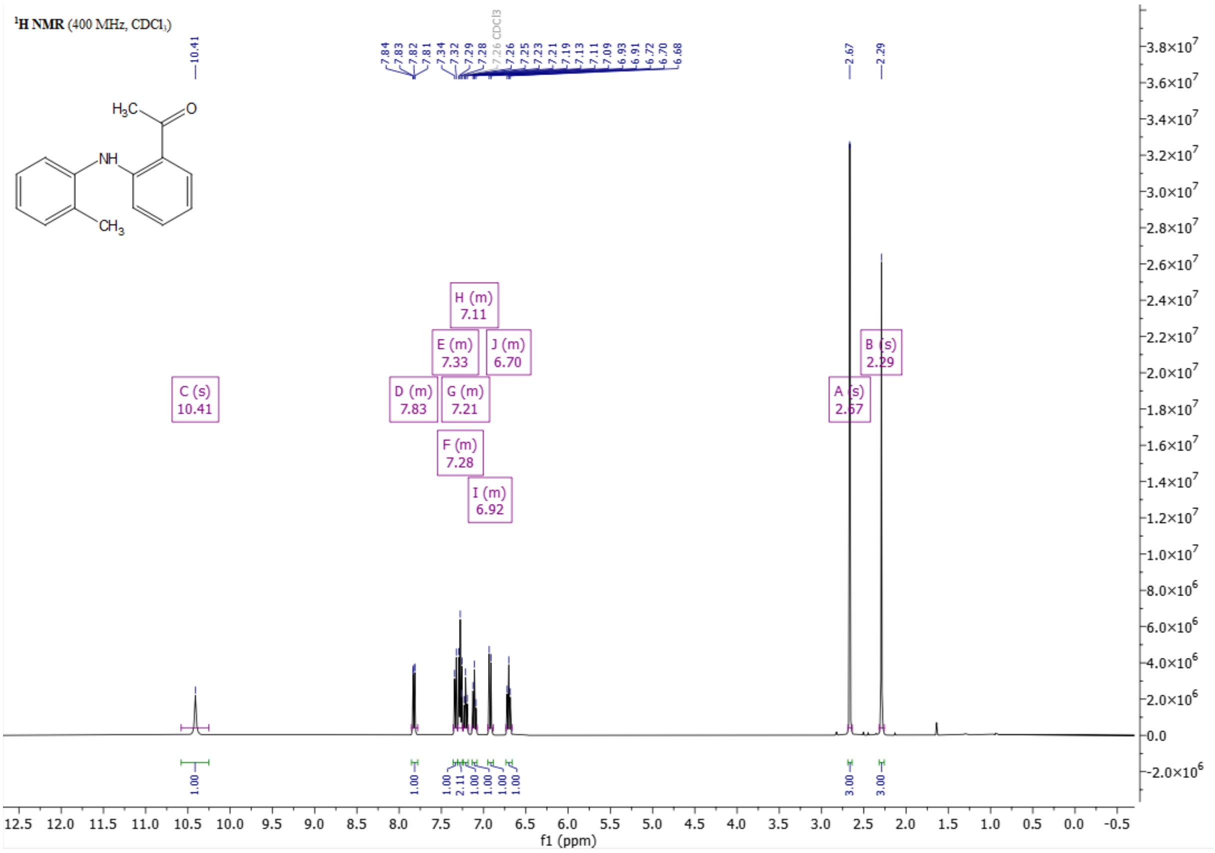

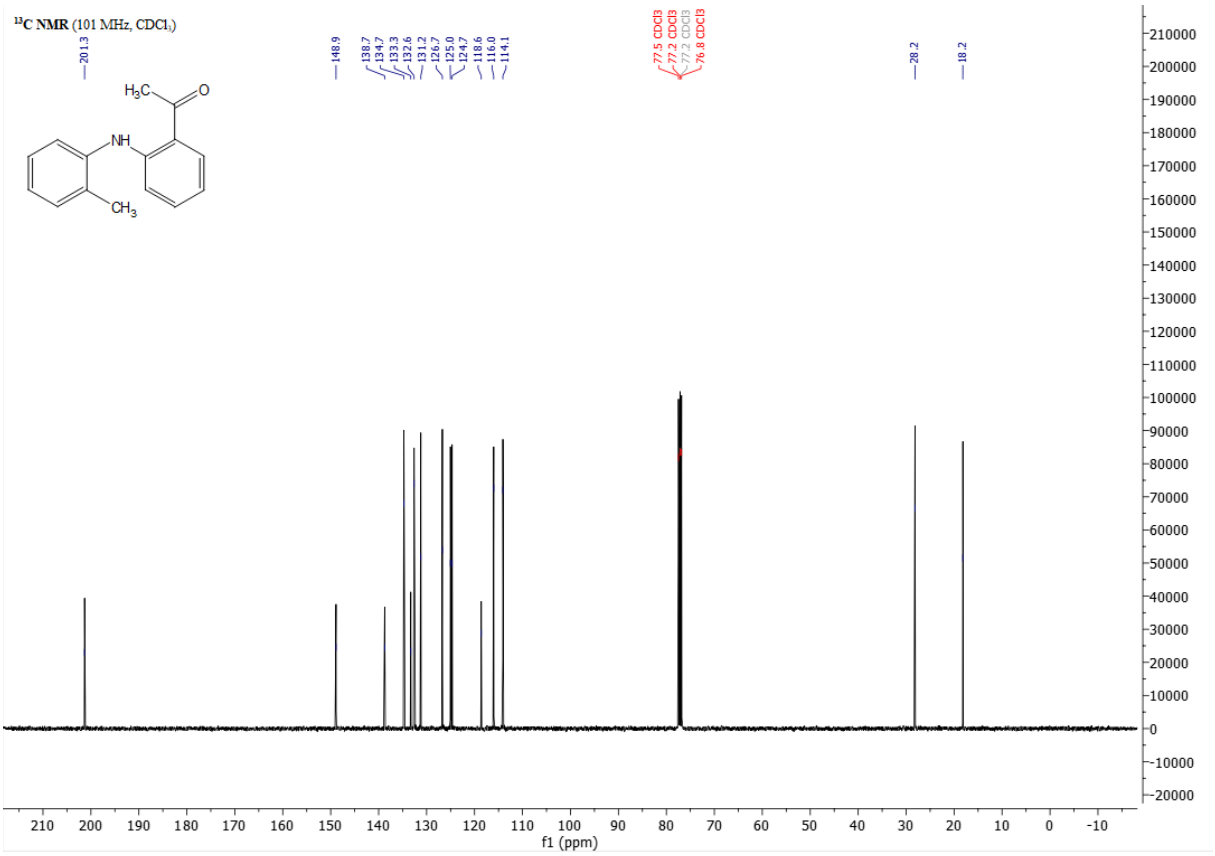

1-(2-(*m*-tolylamino)phenyl)ethan-1-one (5)

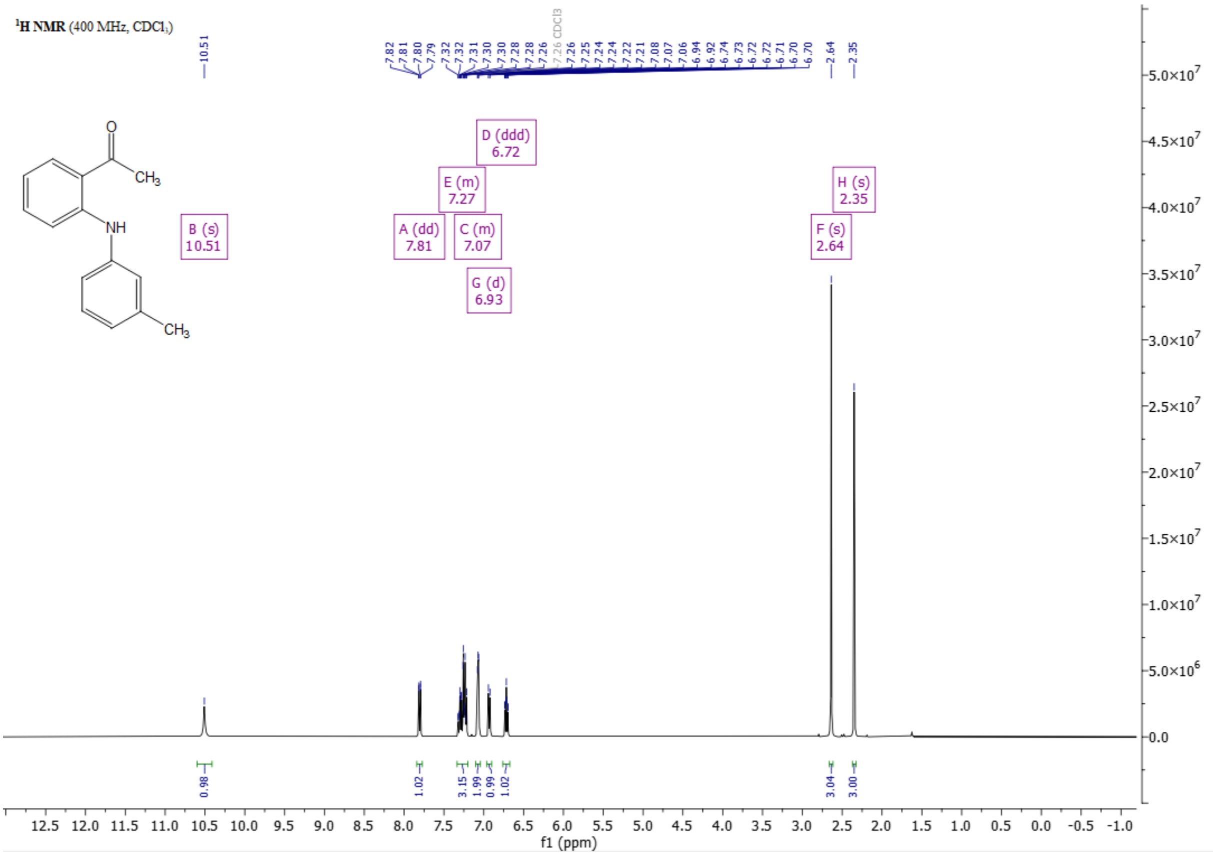

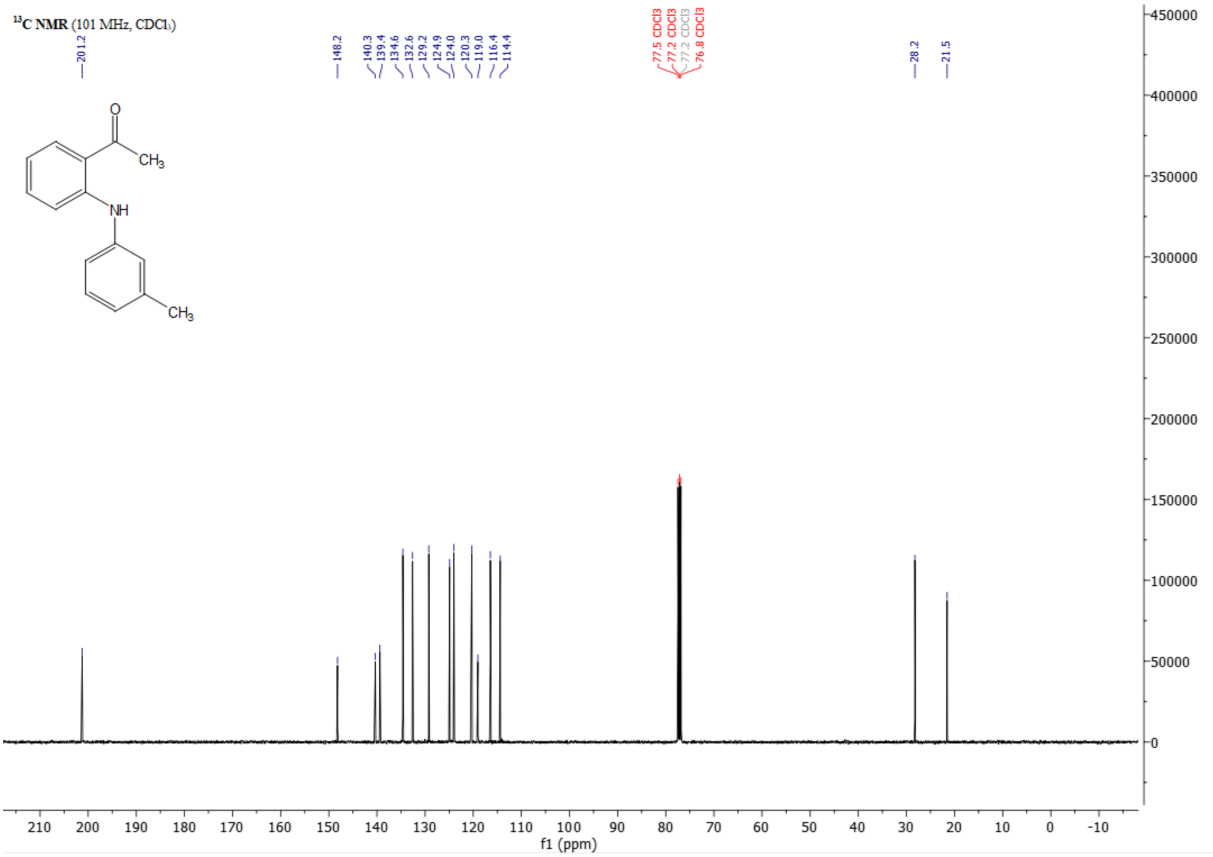

1-(2-(*p*-tolylamino)phenyl)ethan-1-one (6)

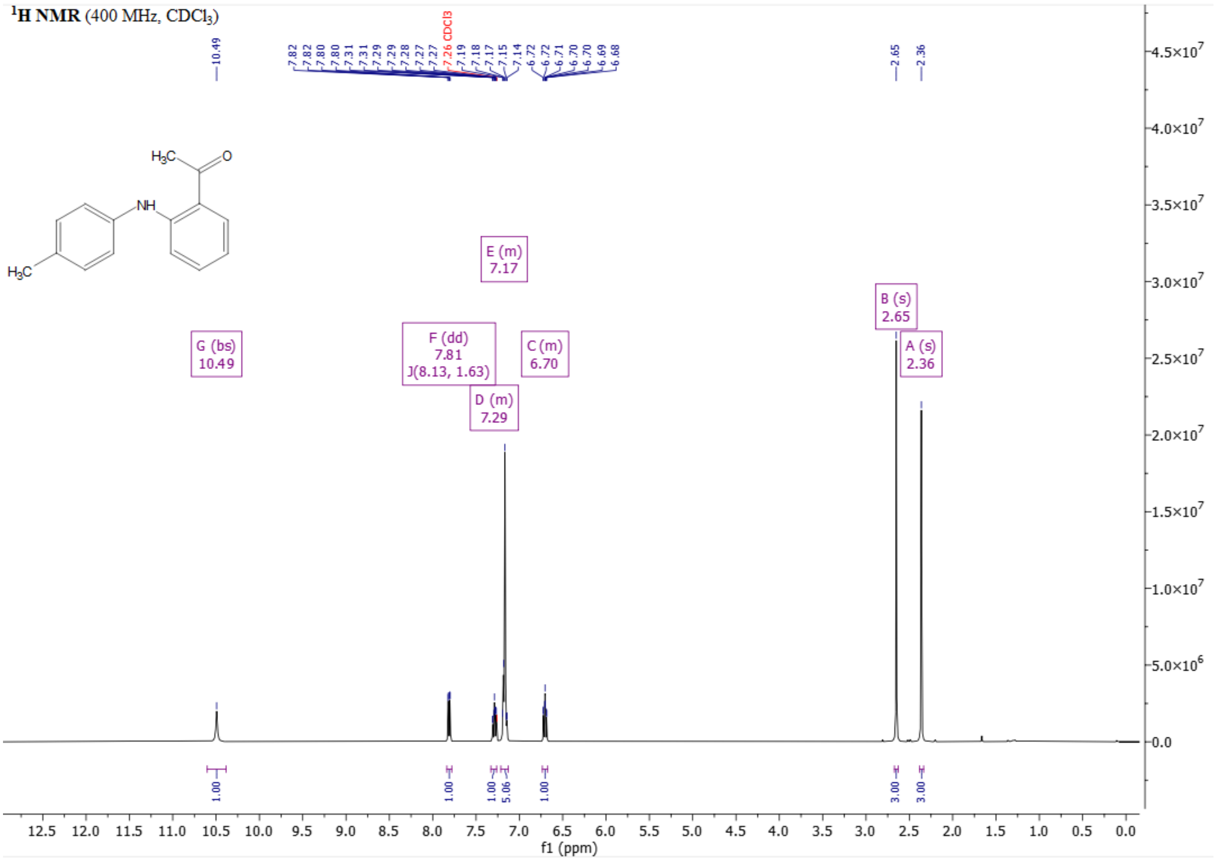

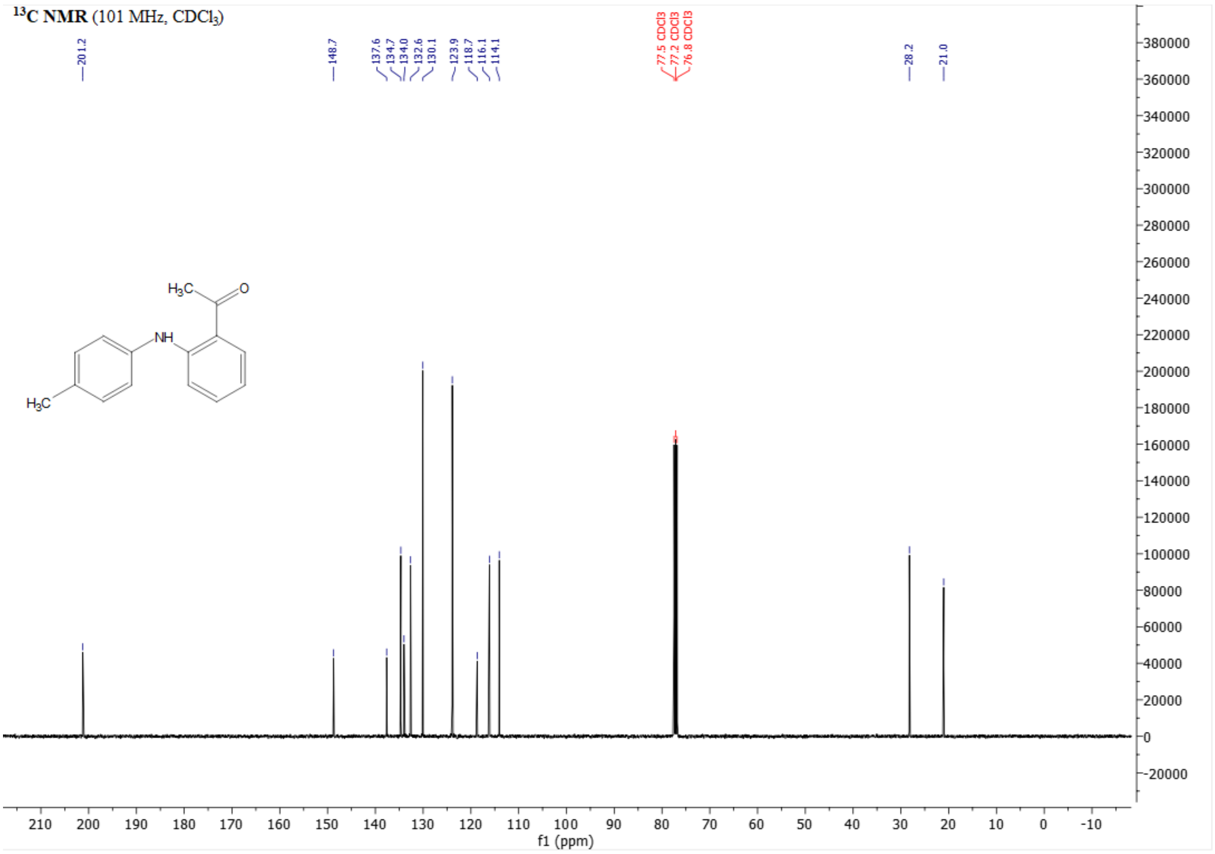

1-(2-((4-(tert-butyl)phenyl)amino)phenyl)ethan-1-one (7)

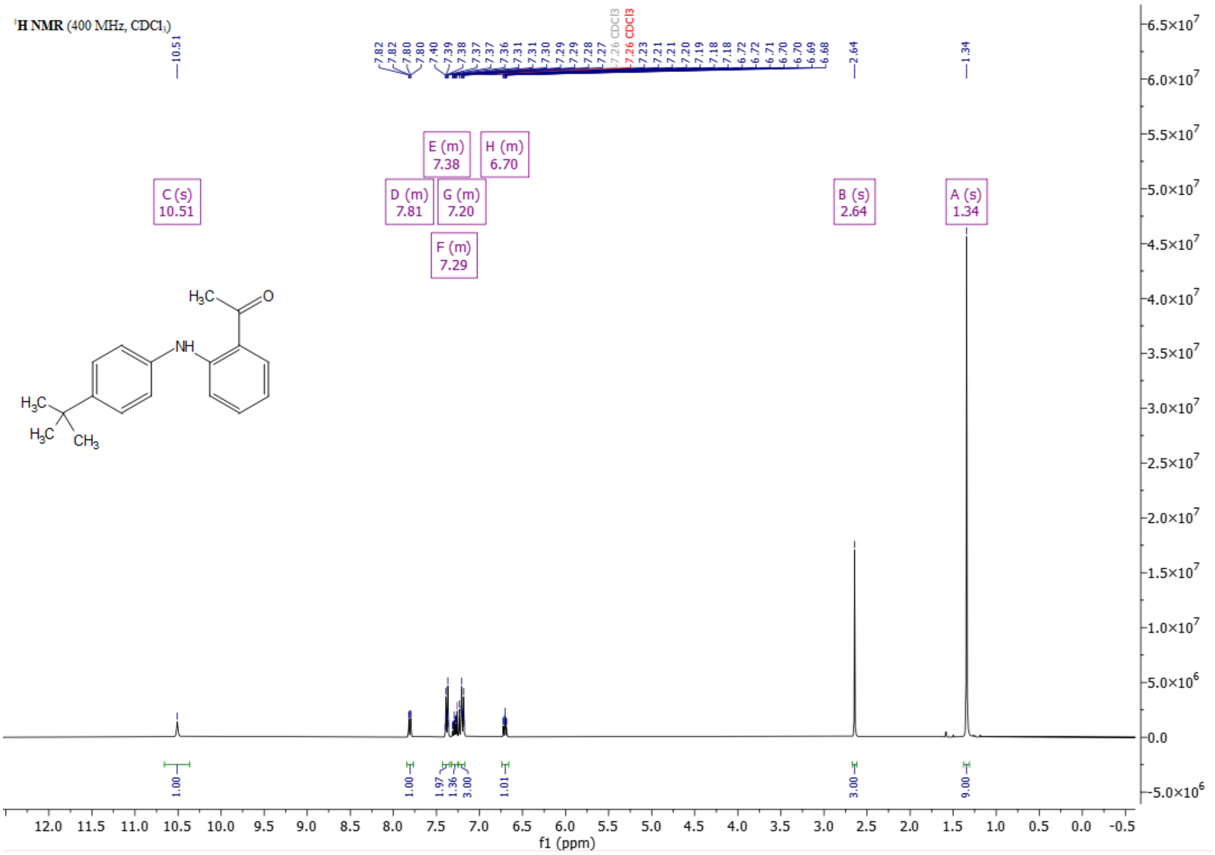

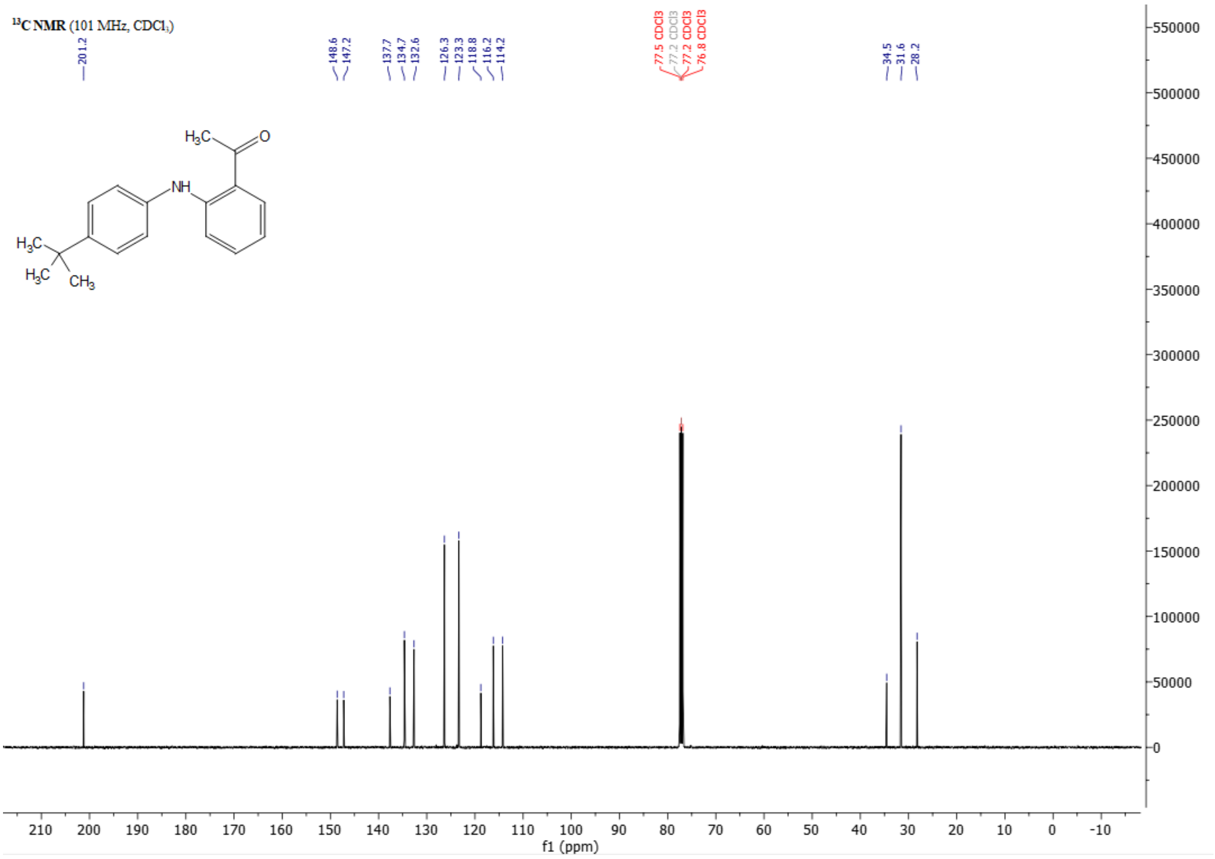

1-(2-((4-(dimethylamino)phenyl)amino)phenyl)ethan-1-one (8)

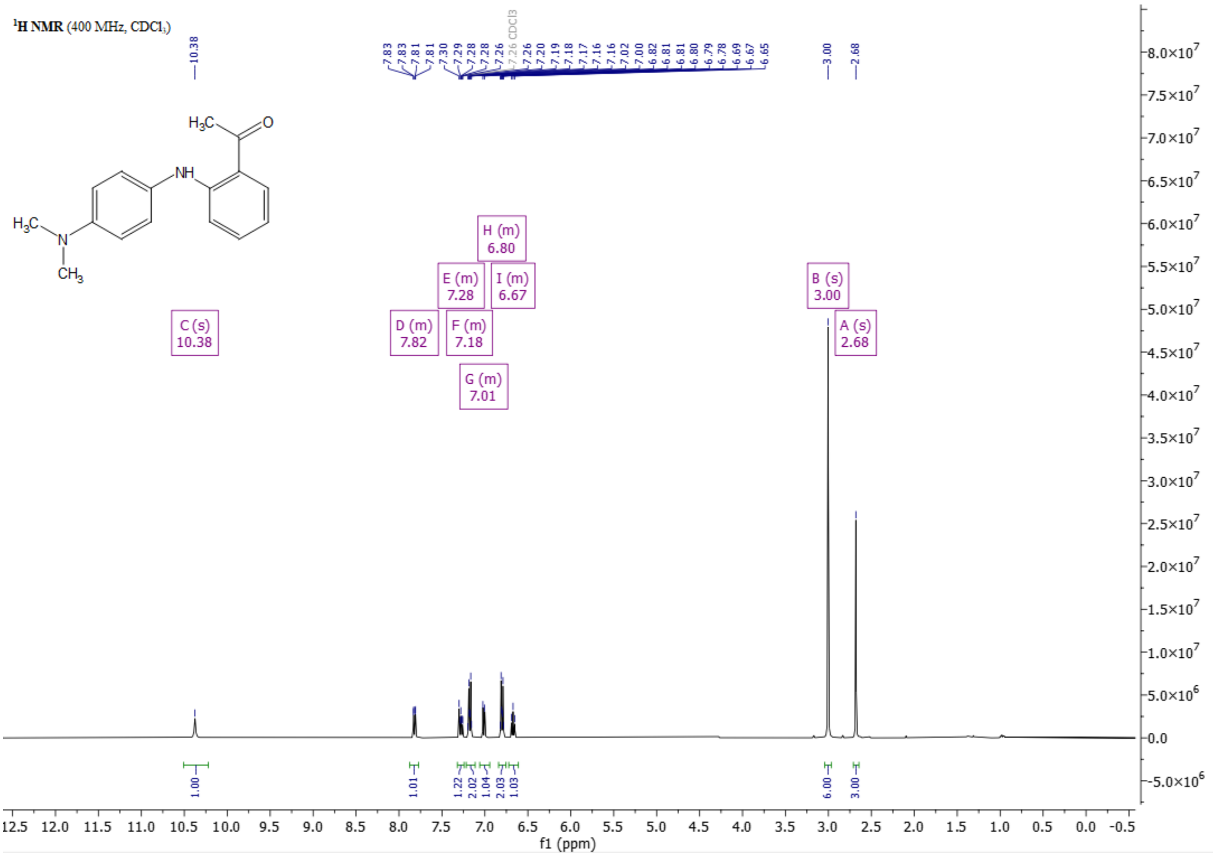

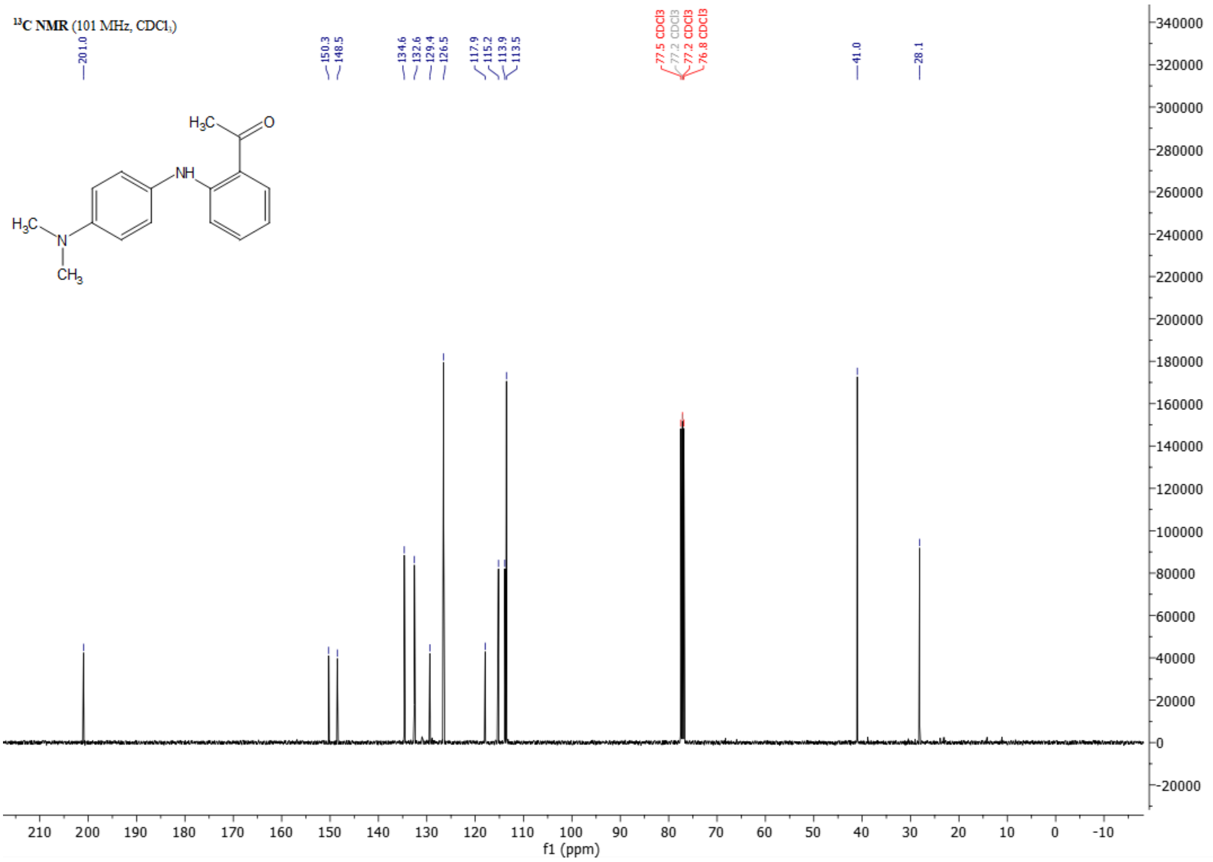

1-(2-((4-methoxyphenyl)amino)phenyl)ethan-1-one (9)

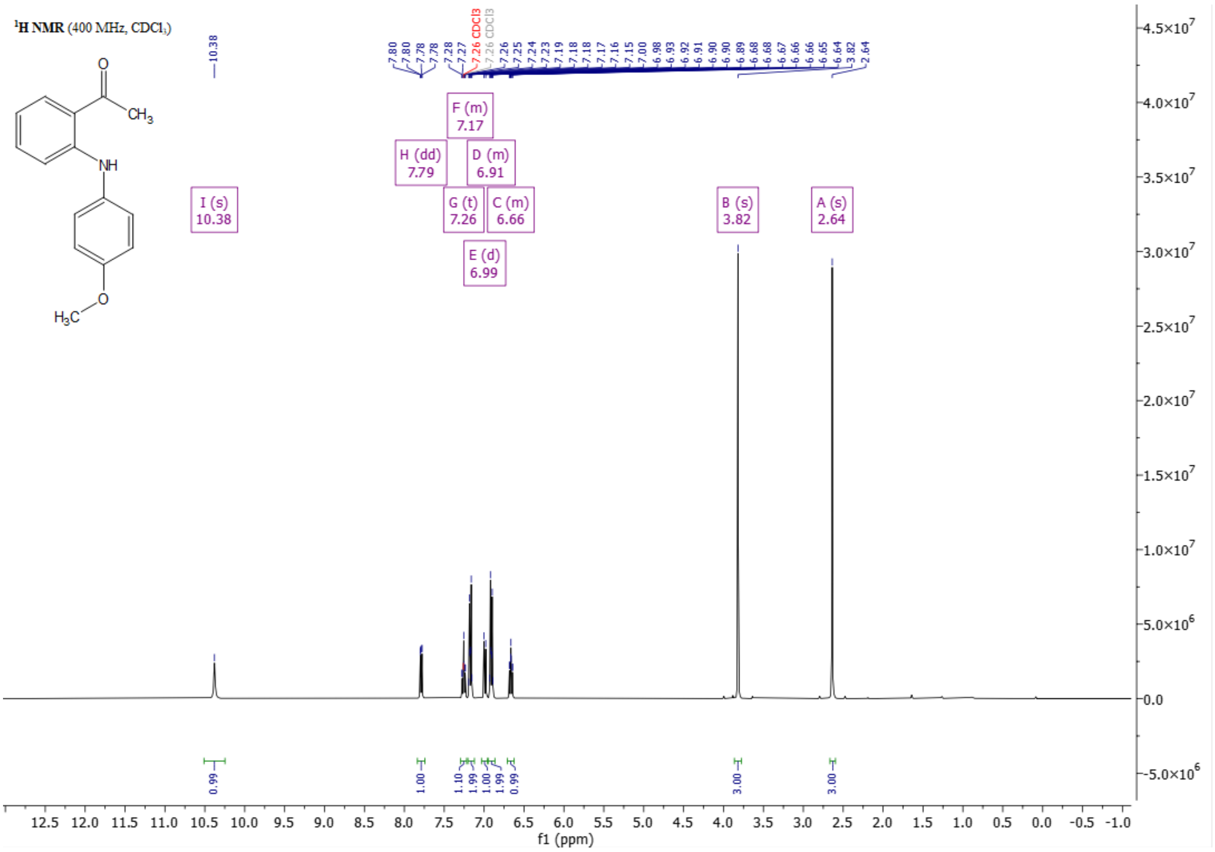

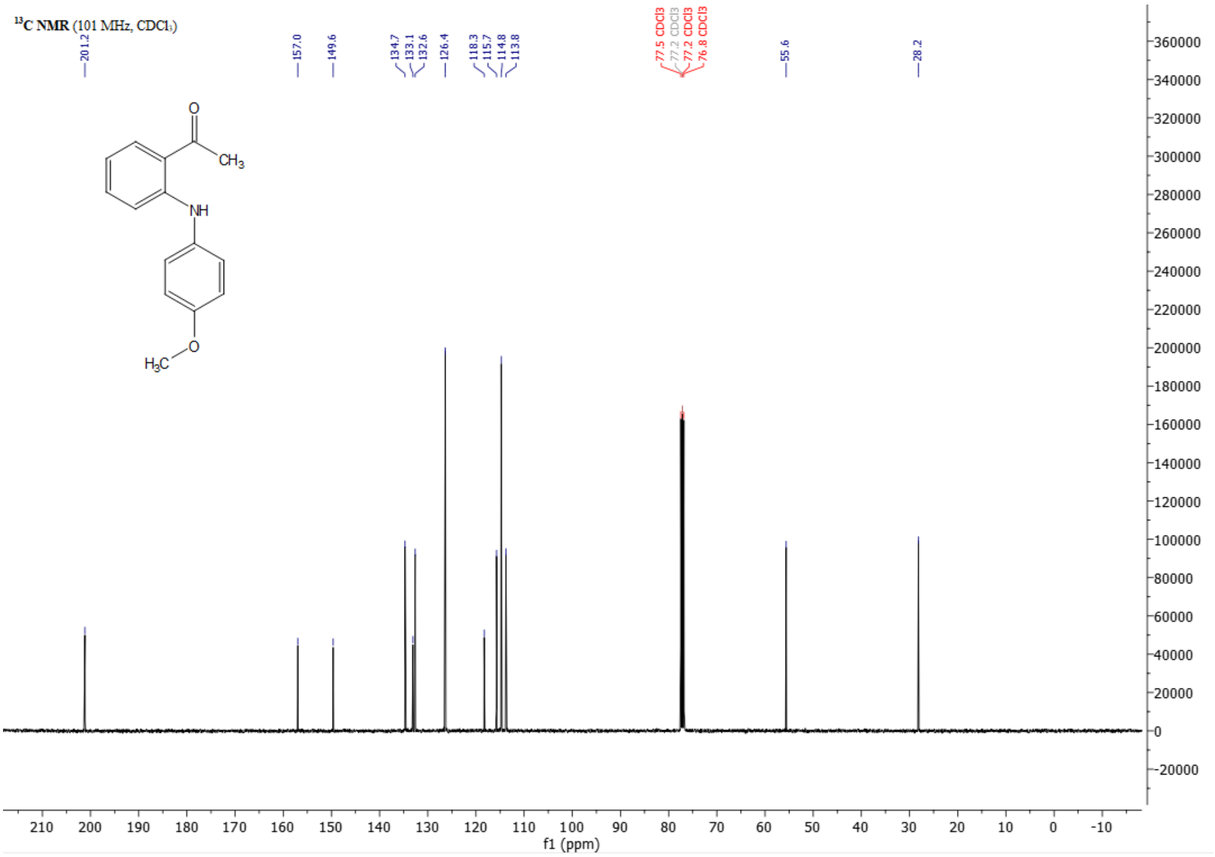

1-(2-((4-(trifluoromethyl)phenyl)amino)phenyl)ethan-1-one (10)

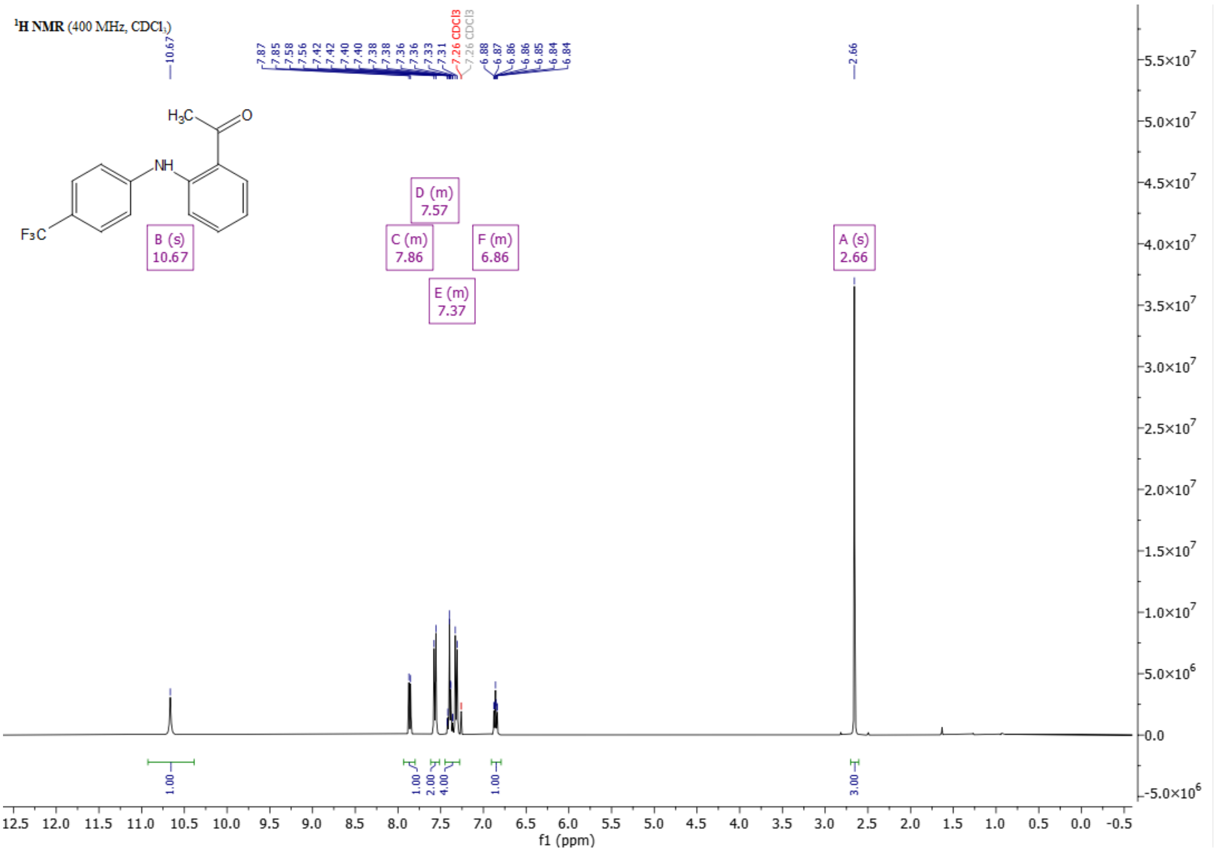

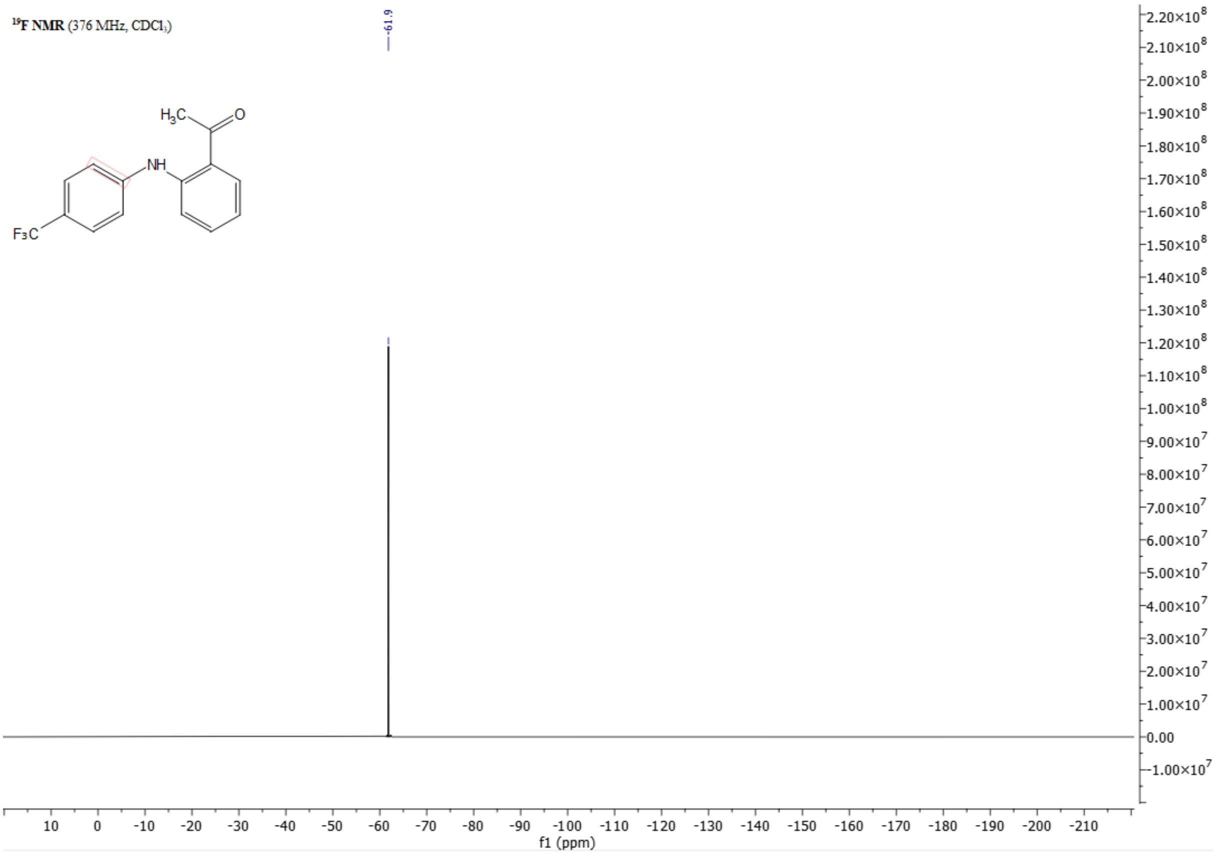

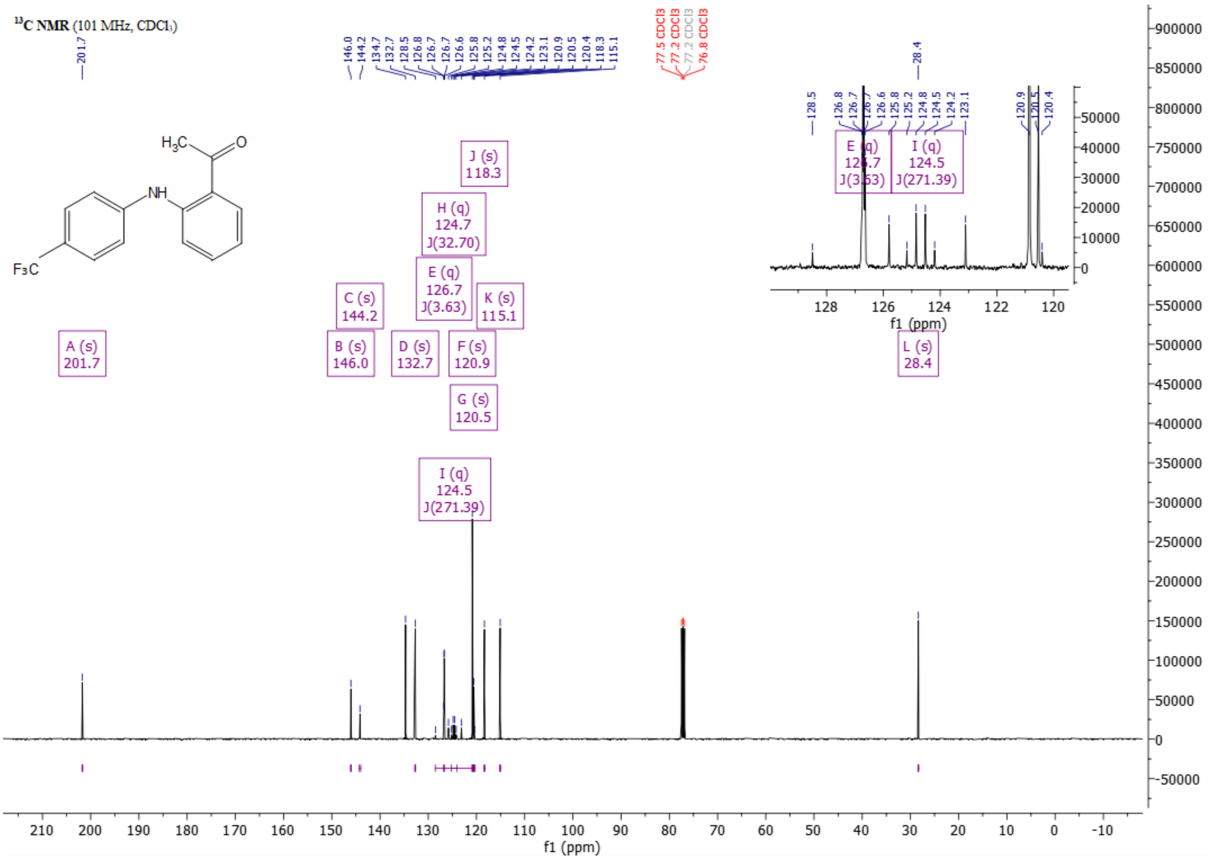

^{13}C NMR (101 MHz, CDCl$_3$)
H3C
O
NH
F3C
A (s) 201.7
B (s) 146.0
C (s) 144.2
D (s) 132.7
E (q) 126.7 J(3.63)
F (s) 120.9
G (s) 120.5
H (q) 124.7 J(32.70)
I (q) 124.5 J(271.39)
J (s) 118.3
K (s) 115.1
L (s) 28.4
f1 (ppm)

4-((2-acetylphenyl)amino)benzonitrile (11)

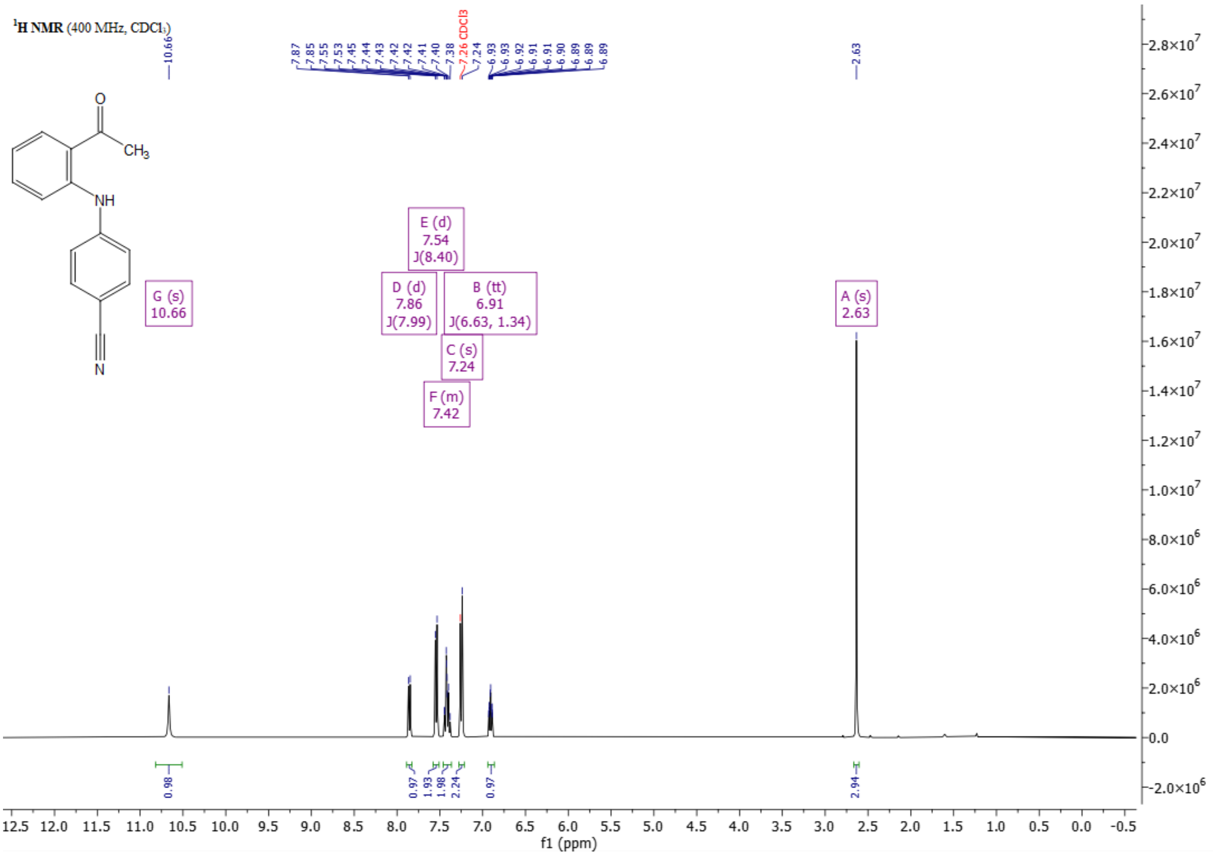

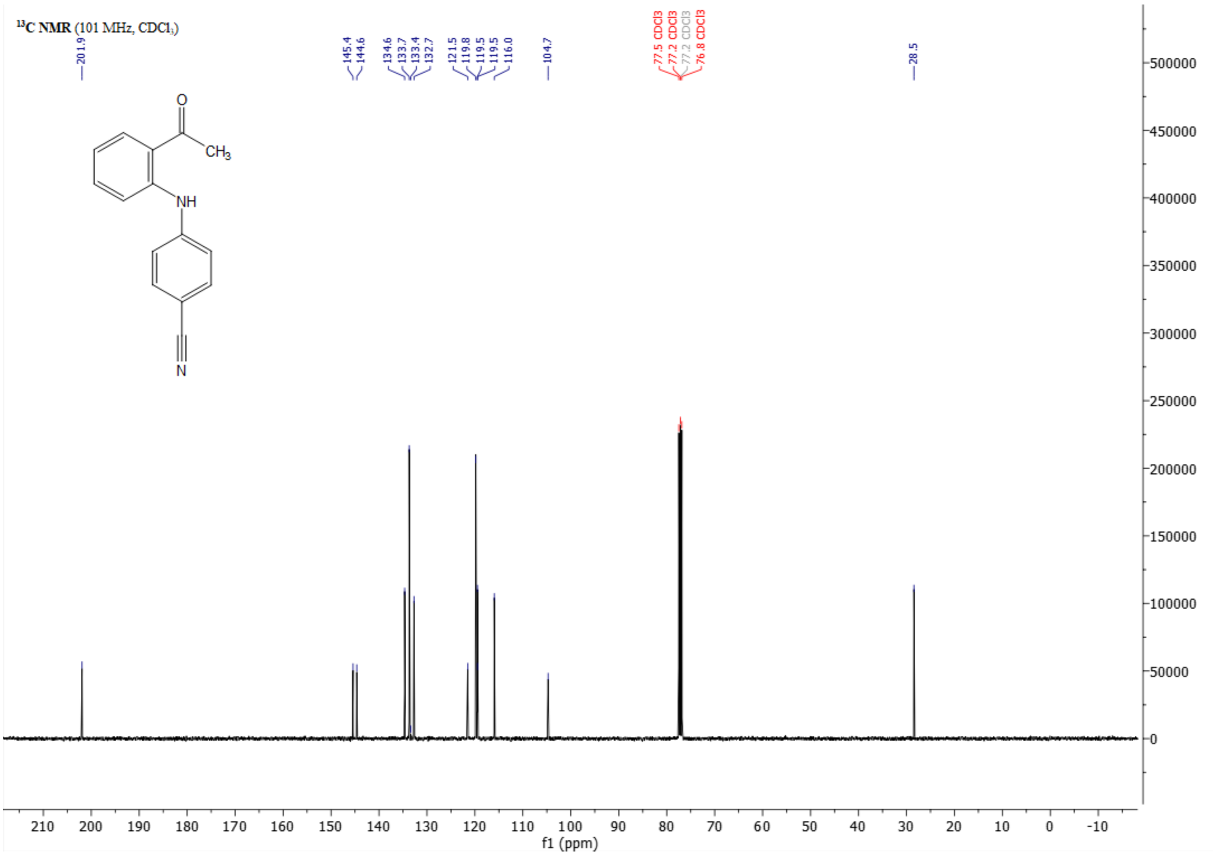

Methyl 2-(4-((2-acetylphenyl)amino)phenyl)-2-methylpropanoate (12)

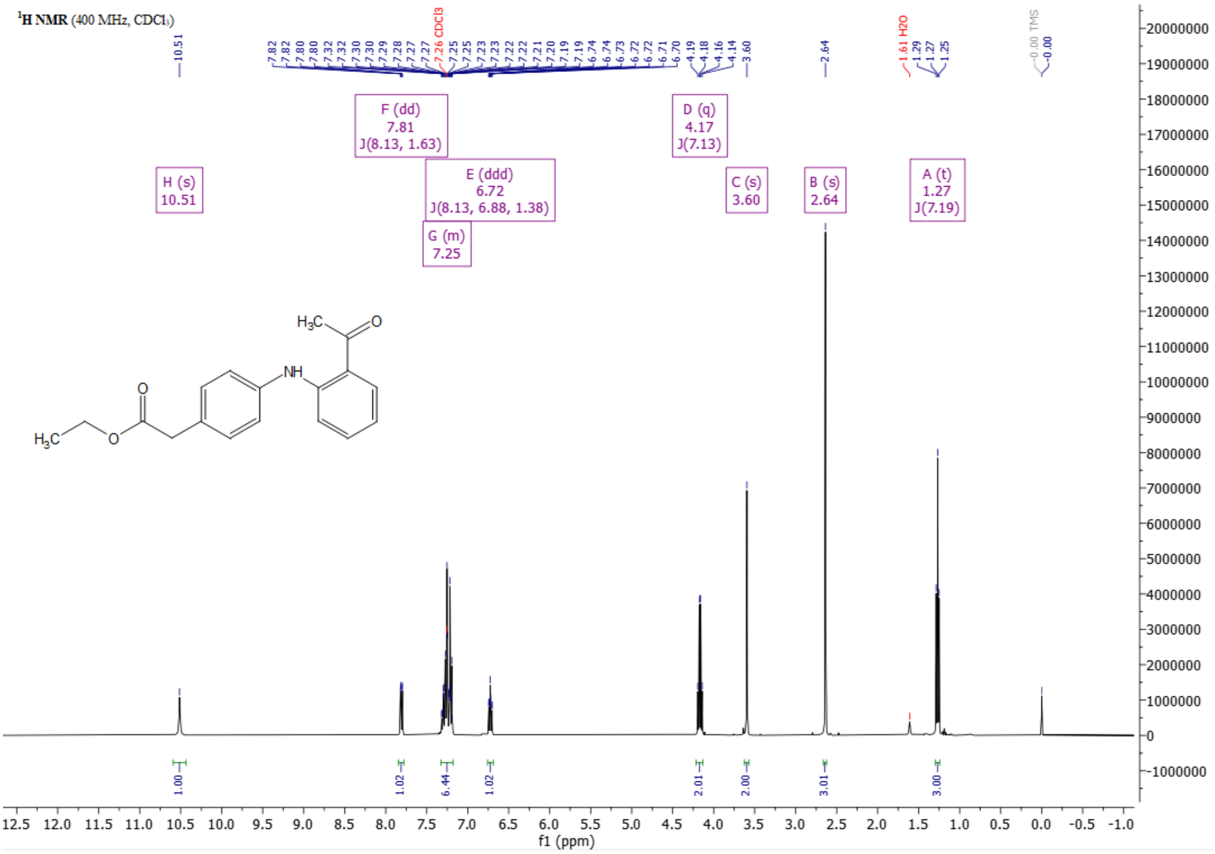

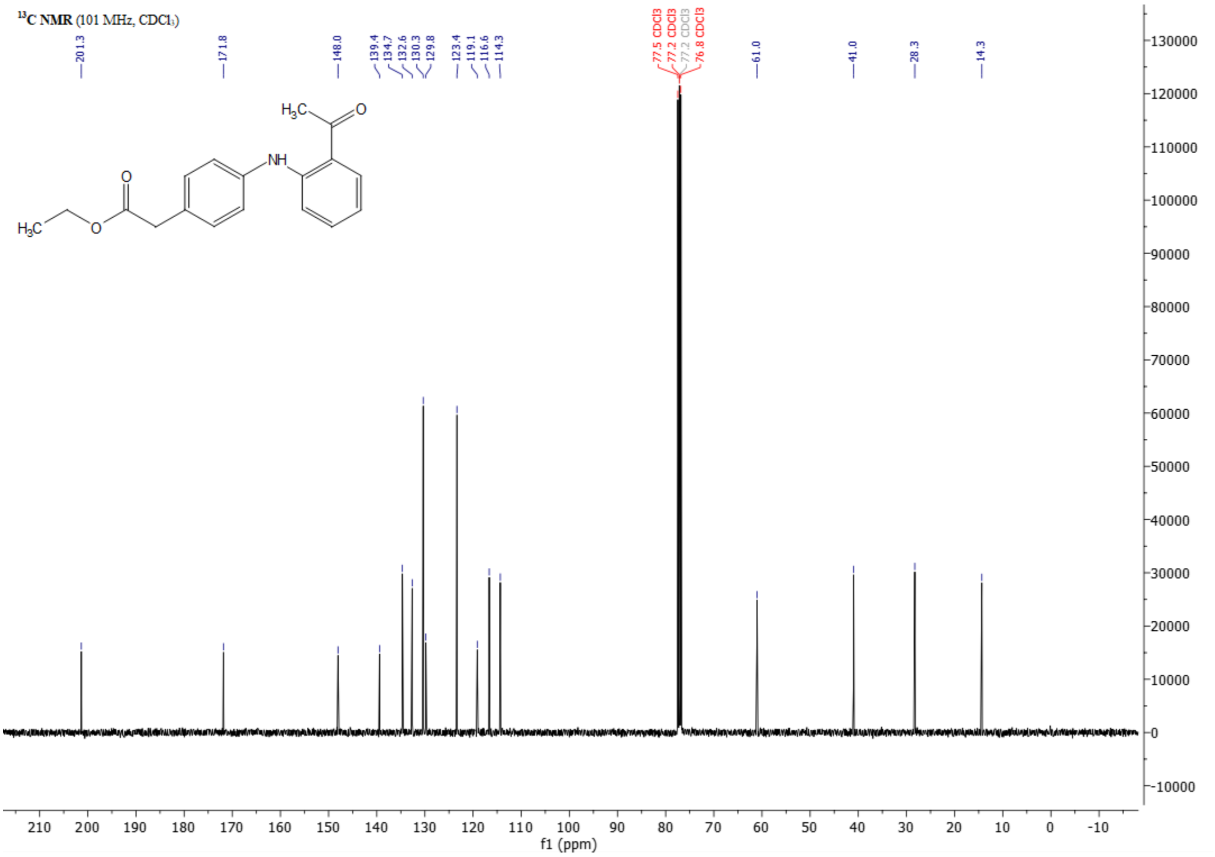

Ethyl 2-(4-((2-acetylphenyl)amino)phenyl)acetate (13)

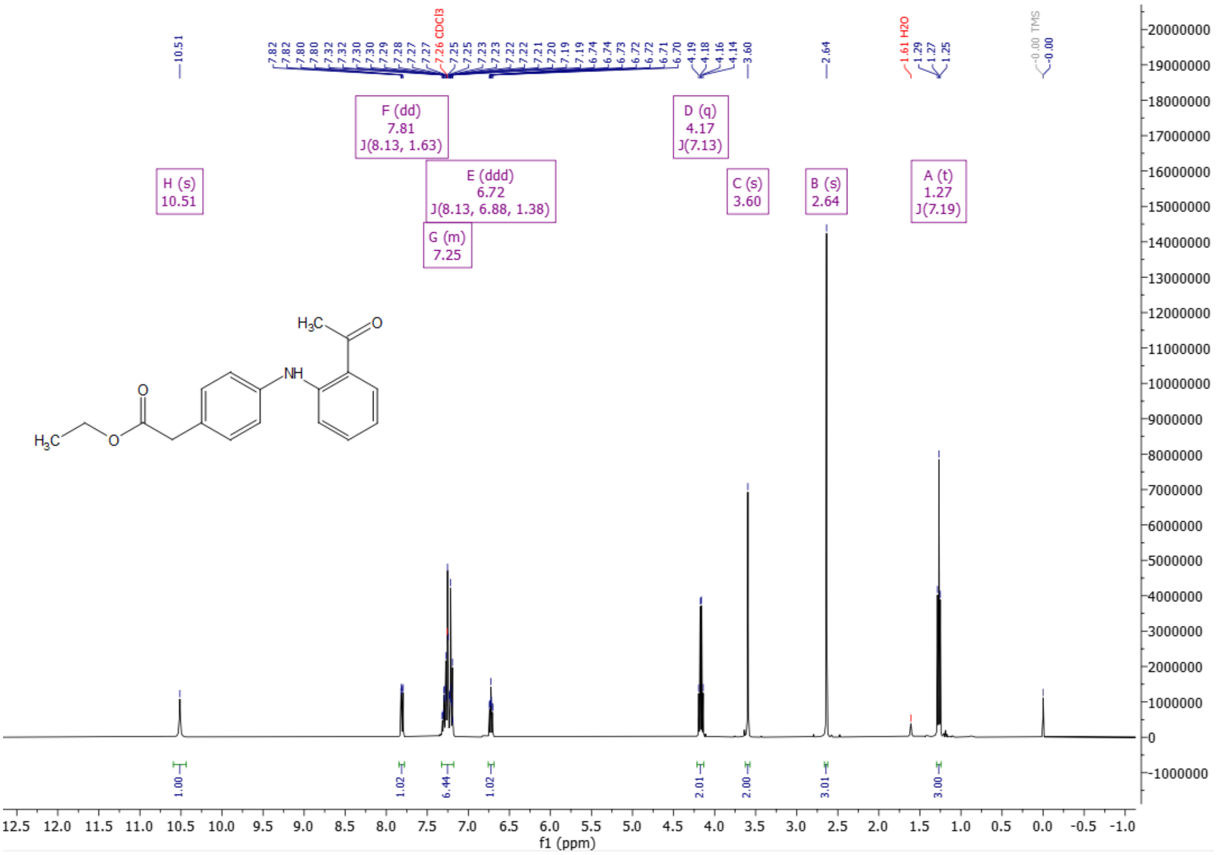

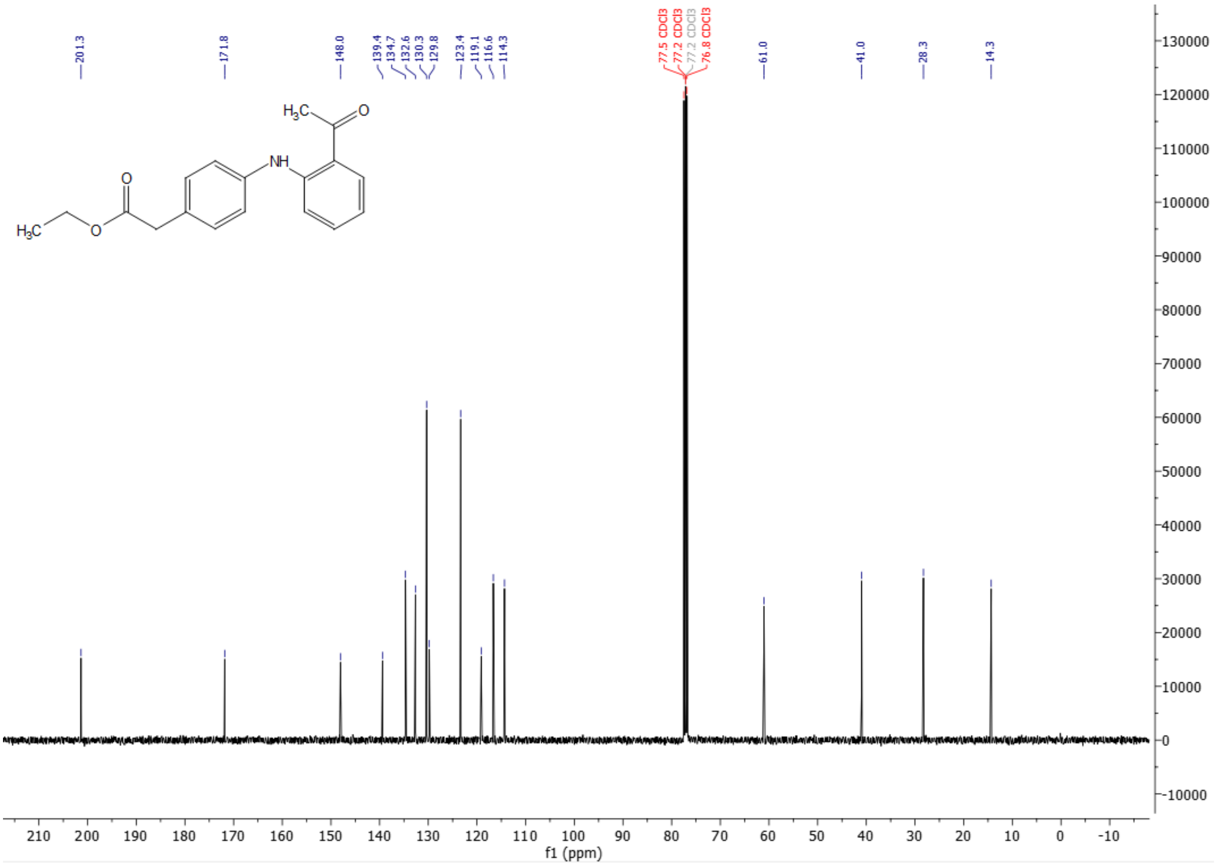

1-(2-((4-fluorophenyl)amino)phenyl)ethan-1-one (14)

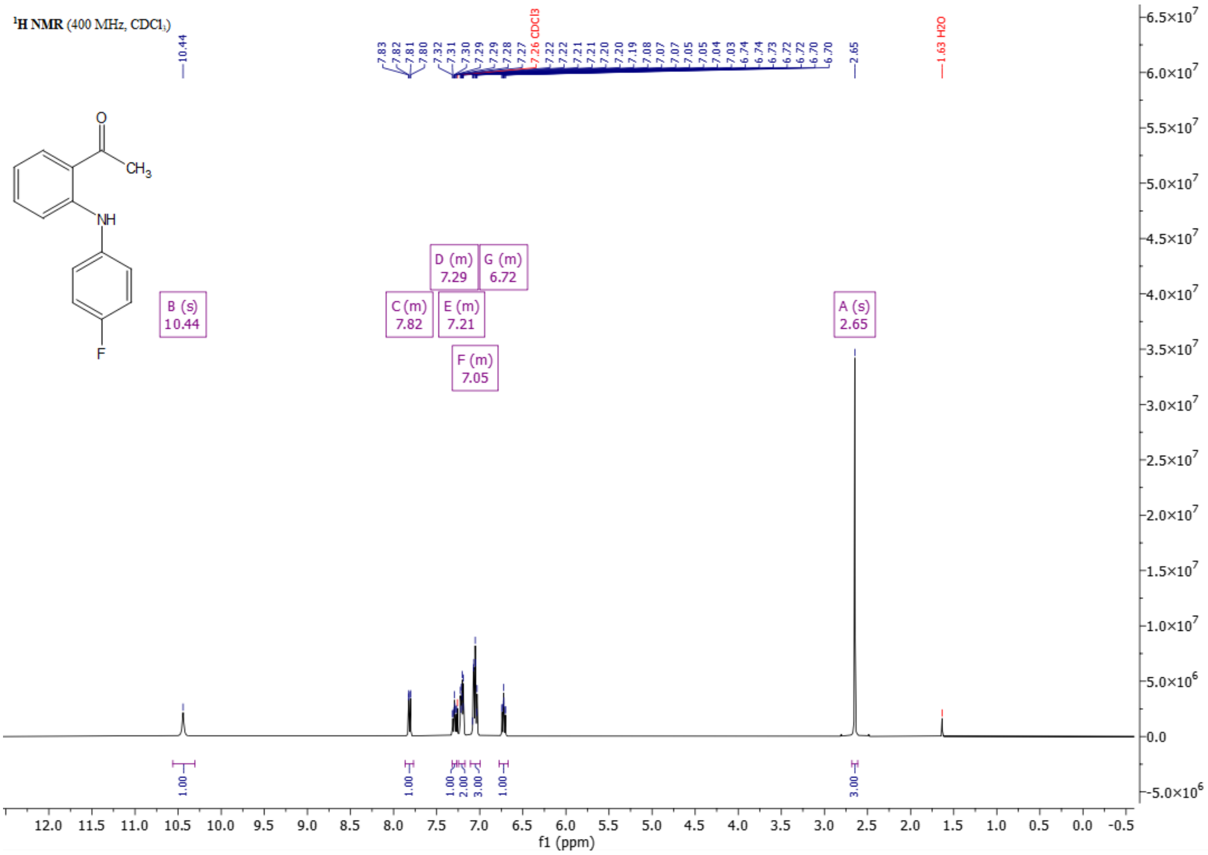

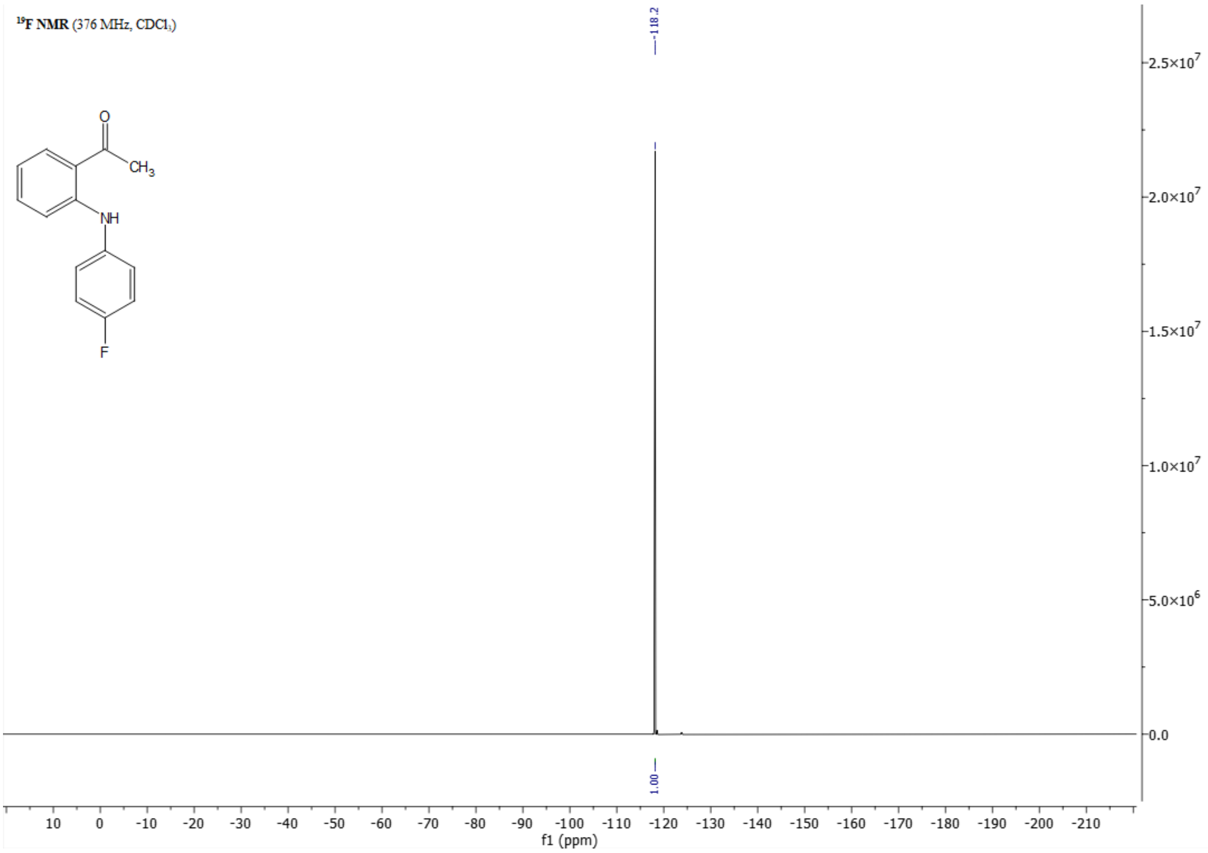

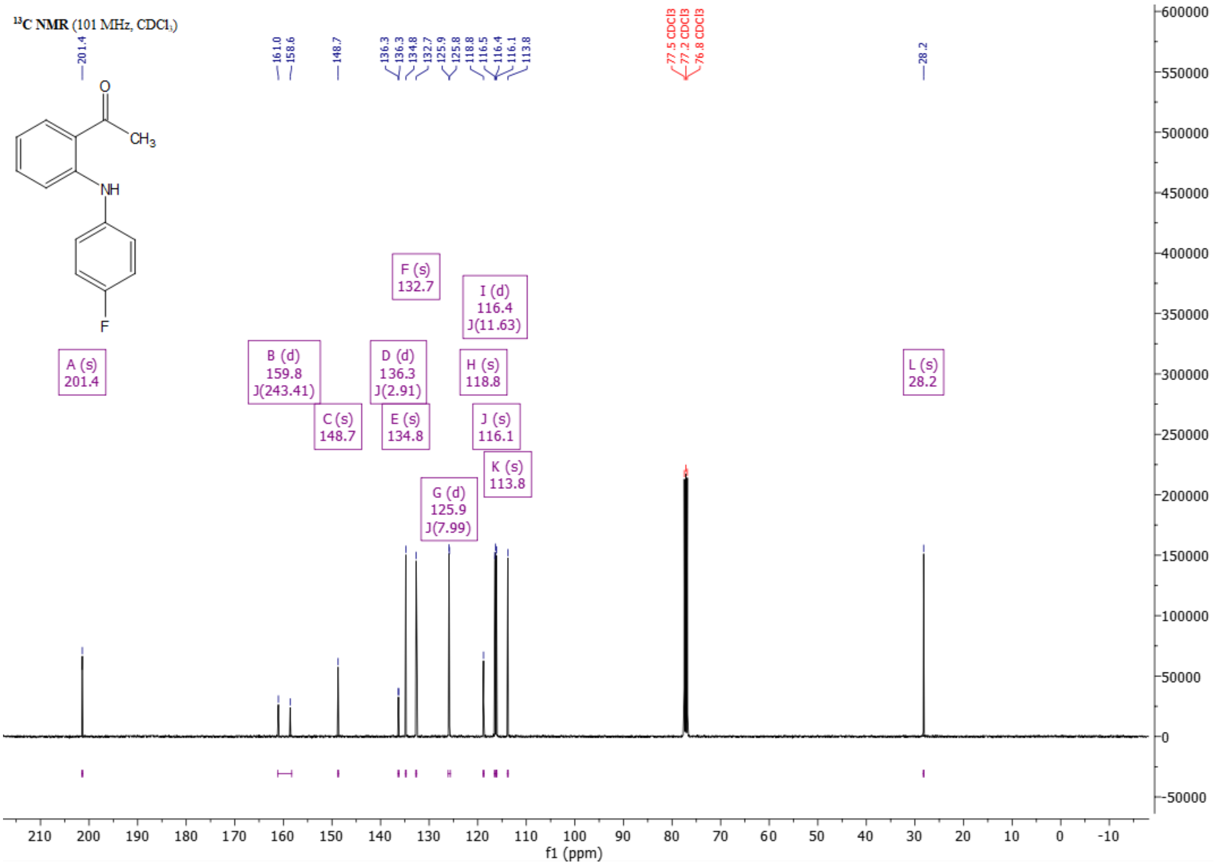

^{13}C NMR (101 MHz, CDCl$_3$)
201.4
161.0
158.6
148.7
136.3
136.3
134.8
132.7
125.9
125.8
118.8
116.5
116.4
116.1
113.8
77.5 CDCl3
77.2 CDCl3
76.8 CDCl3
28.2
O
CH3
NH
F
A (s) 201.4
B (d) 159.8 J(243.41)
C (s) 148.7
D (d) 136.3 J(2.91)
E (s) 134.8
F (s) 132.7
G (d) 125.9 J(7.99)
H (s) 118.8
I (d) 116.4 J(11.63)
J (s) 116.1
K (s) 113.8
L (s) 28.2
f1 (ppm)

1-(2-((2-fluorophenyl)amino)phenyl)ethan-1-one (15)

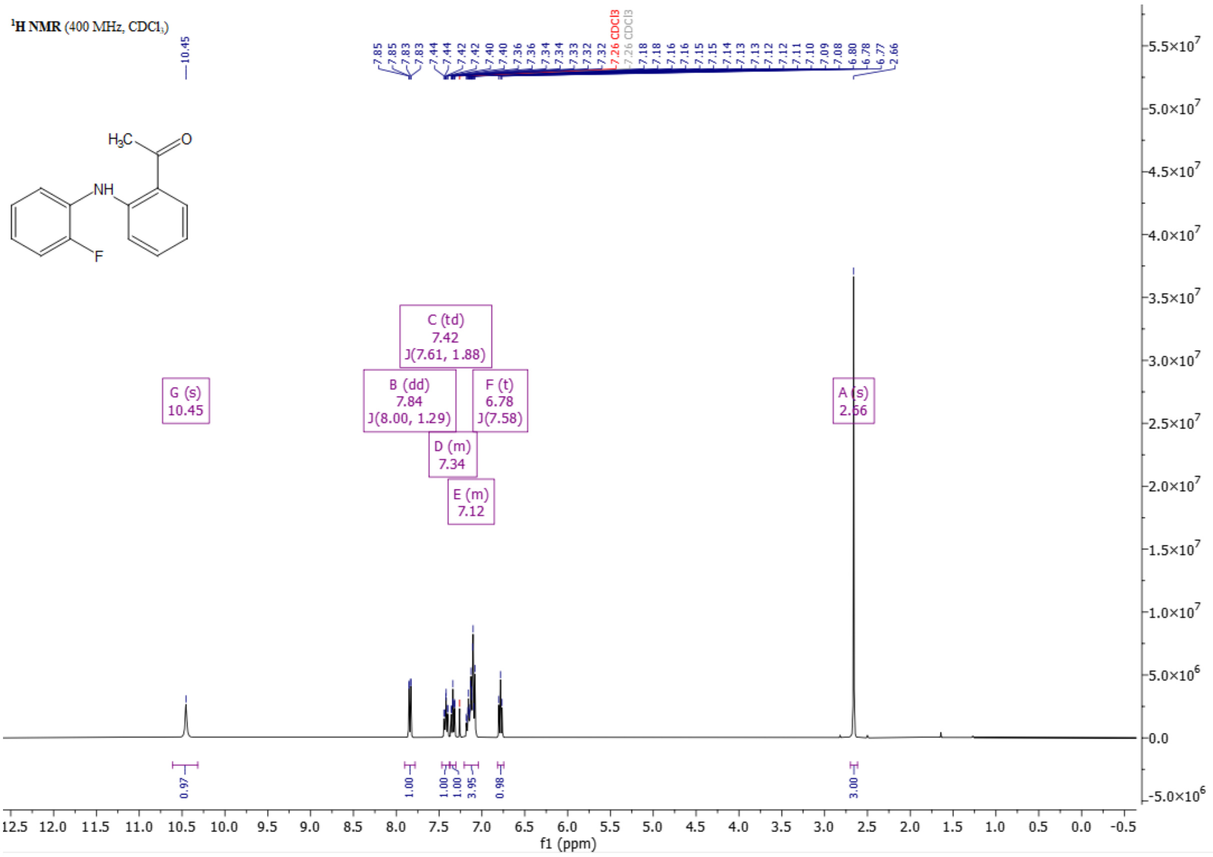

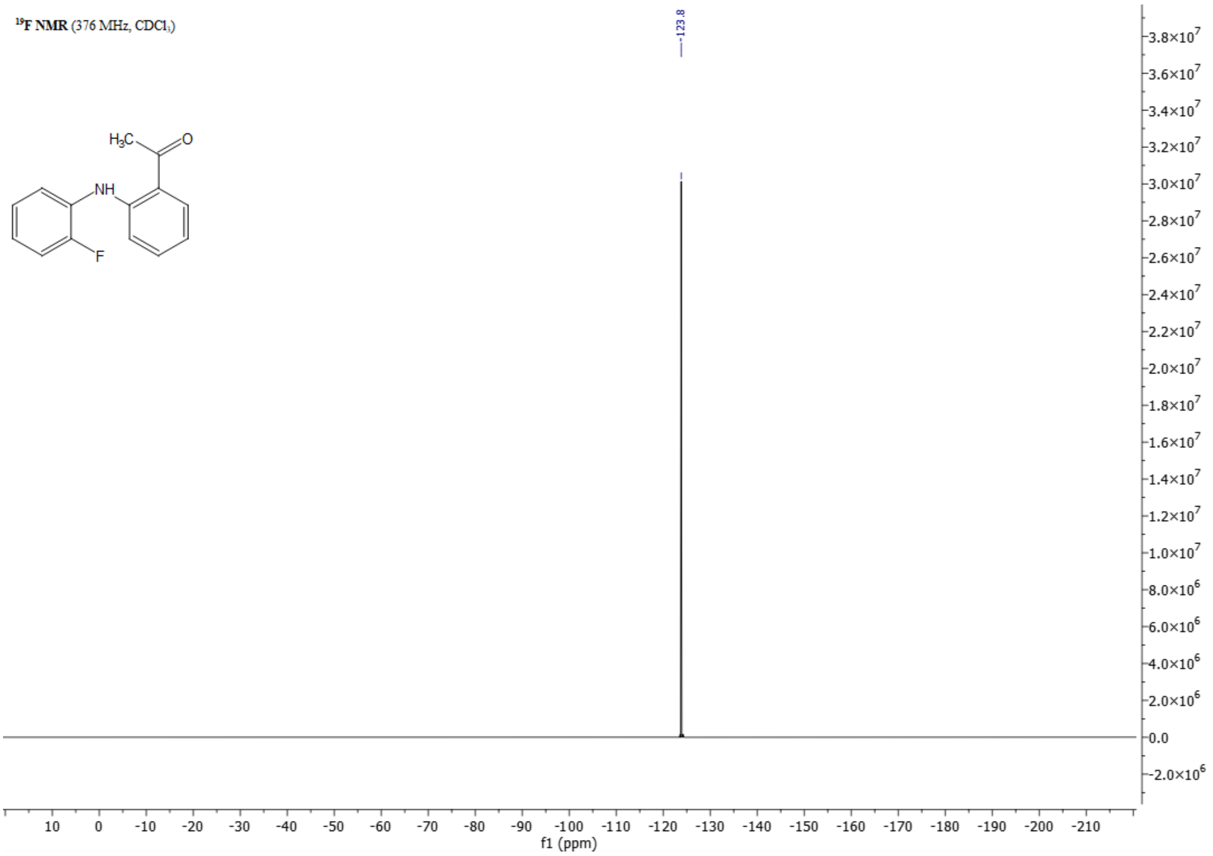

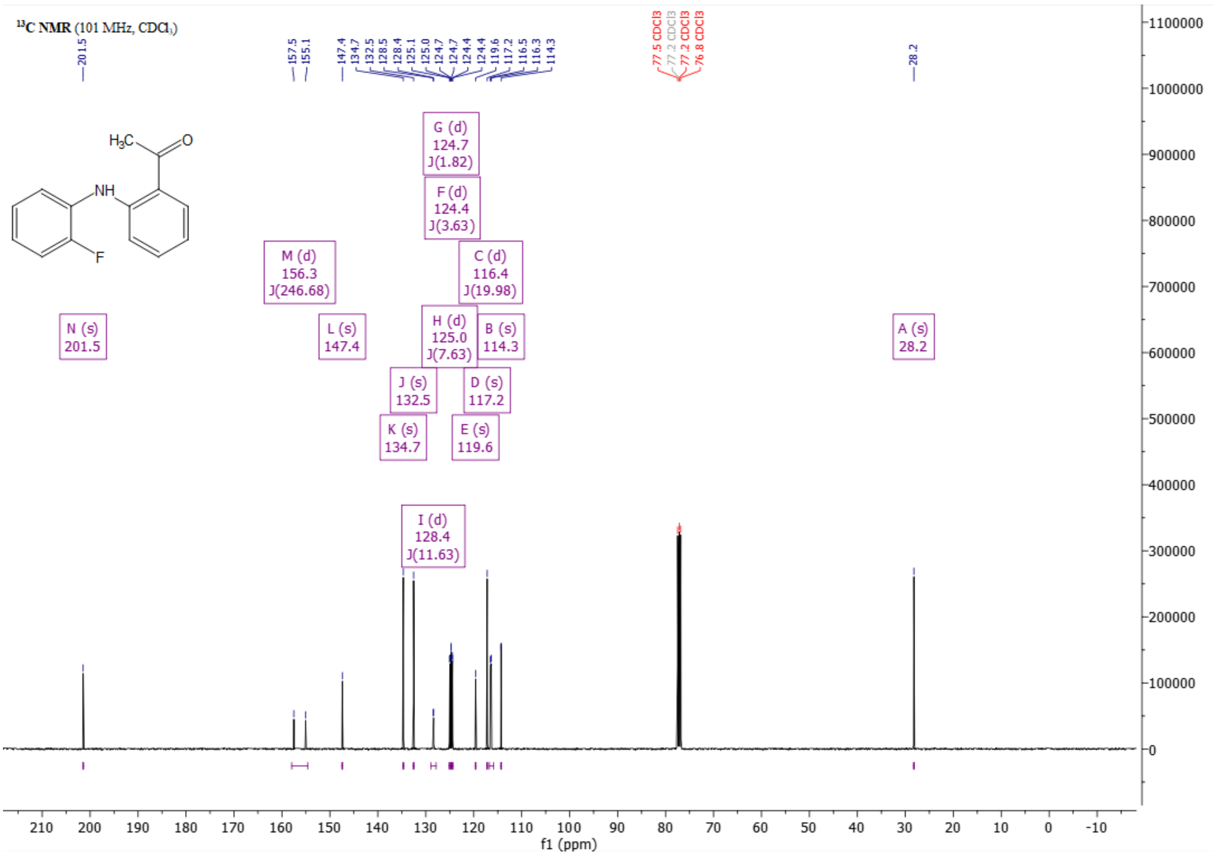

13C NMR (101 MHz, CDCl3)
201.5
157.5
155.1
147.4
134.7
132.5
128.5
128.4
125.1
125.0
124.7
124.7
124.4
124.4
119.6
117.2
116.5
116.3
114.3
77.5 CDCl3
77.2 CDCl3
77.2 CDCl3
76.8 CDCl3
28.2
H3C
O
NH
F
G (d) 124.7 J(1.82)
F (d) 124.4 J(3.63)
M (d) 156.3 J(246.68)
C (d) 116.4 J(19.98)
N (s) 201.5
L (s) 147.4
H (d) 125.0 J(7.63)
B (s) 114.3
A (s) 28.2
J (s) 132.5
D (s) 117.2
K (s) 134.7
E (s) 119.6
I (d) 128.4 J(11.63)
f1 (ppm)

1-(2-((4-chlorophenyl)amino)phenyl)ethan-1-one (16)

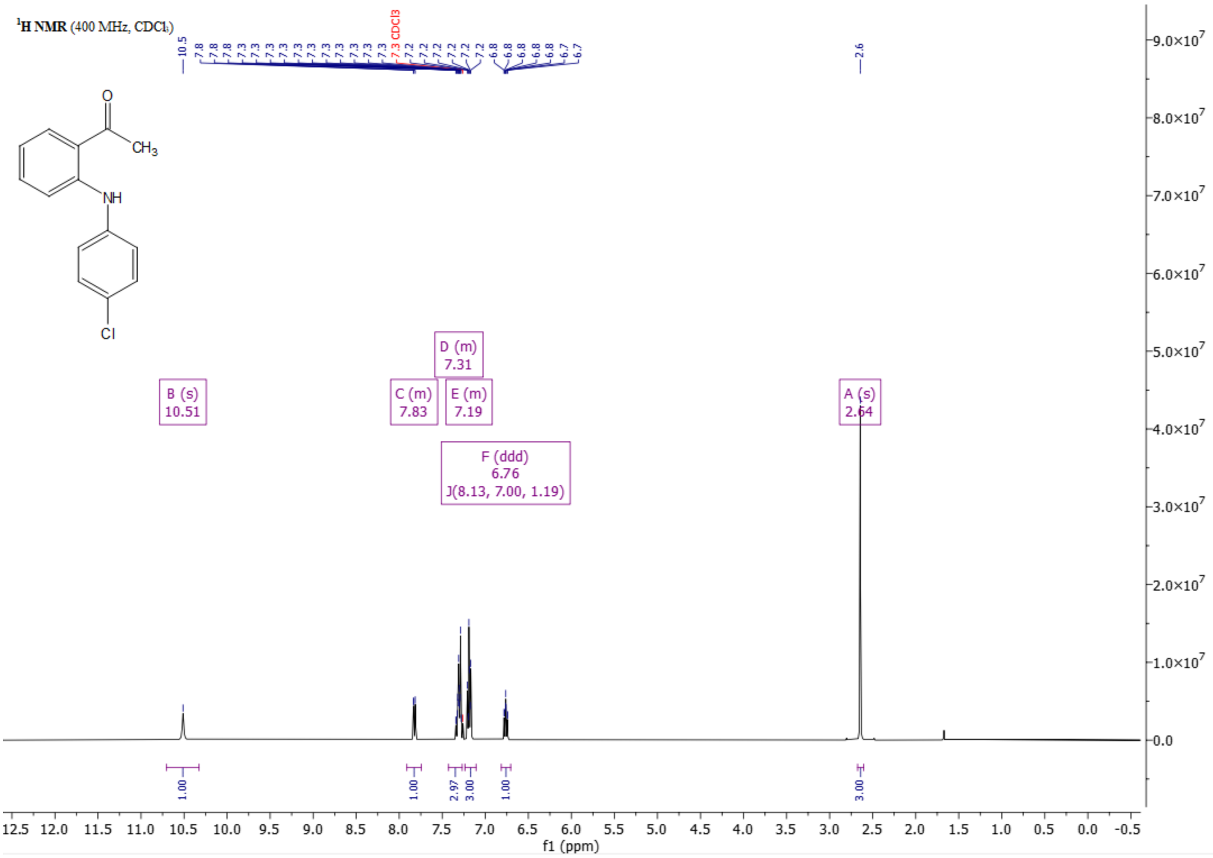

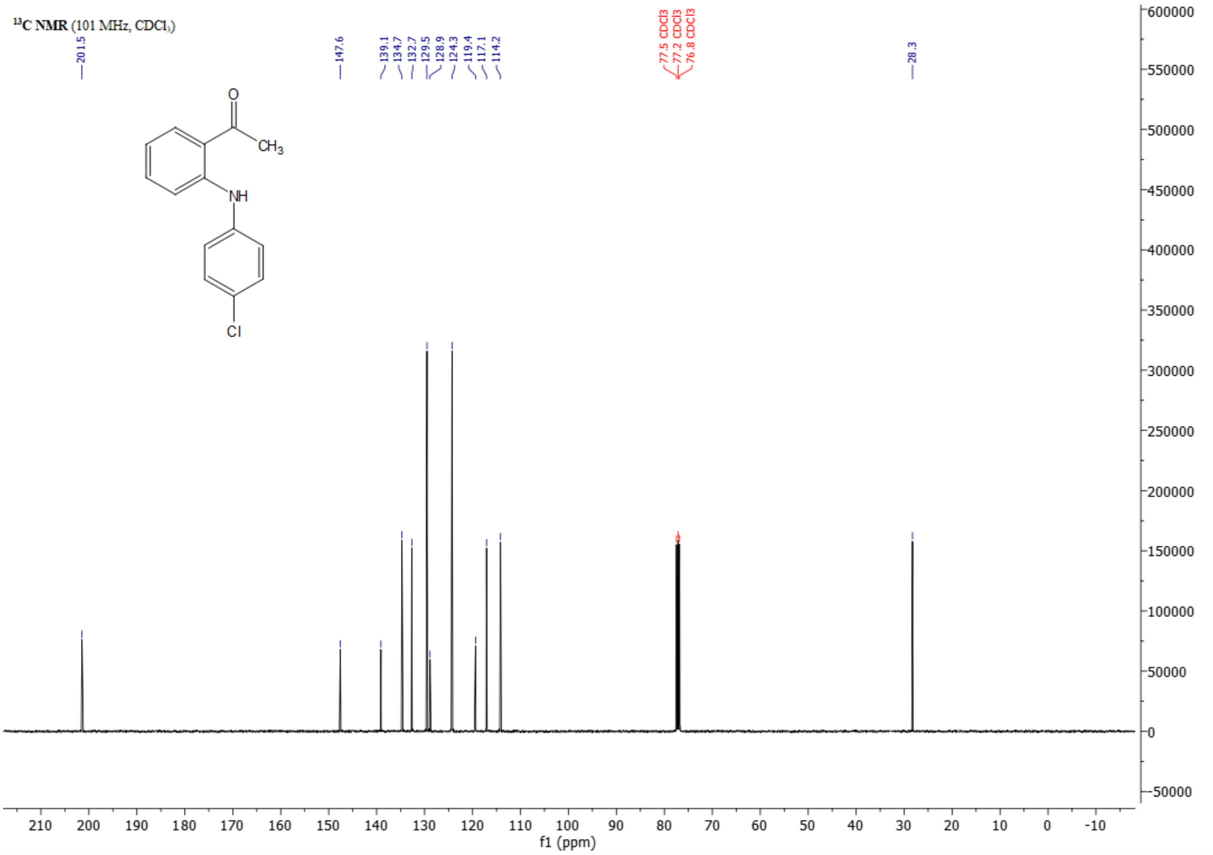

1-(2-(pyridin-3-ylamino)phenyl)ethan-1-one (17)

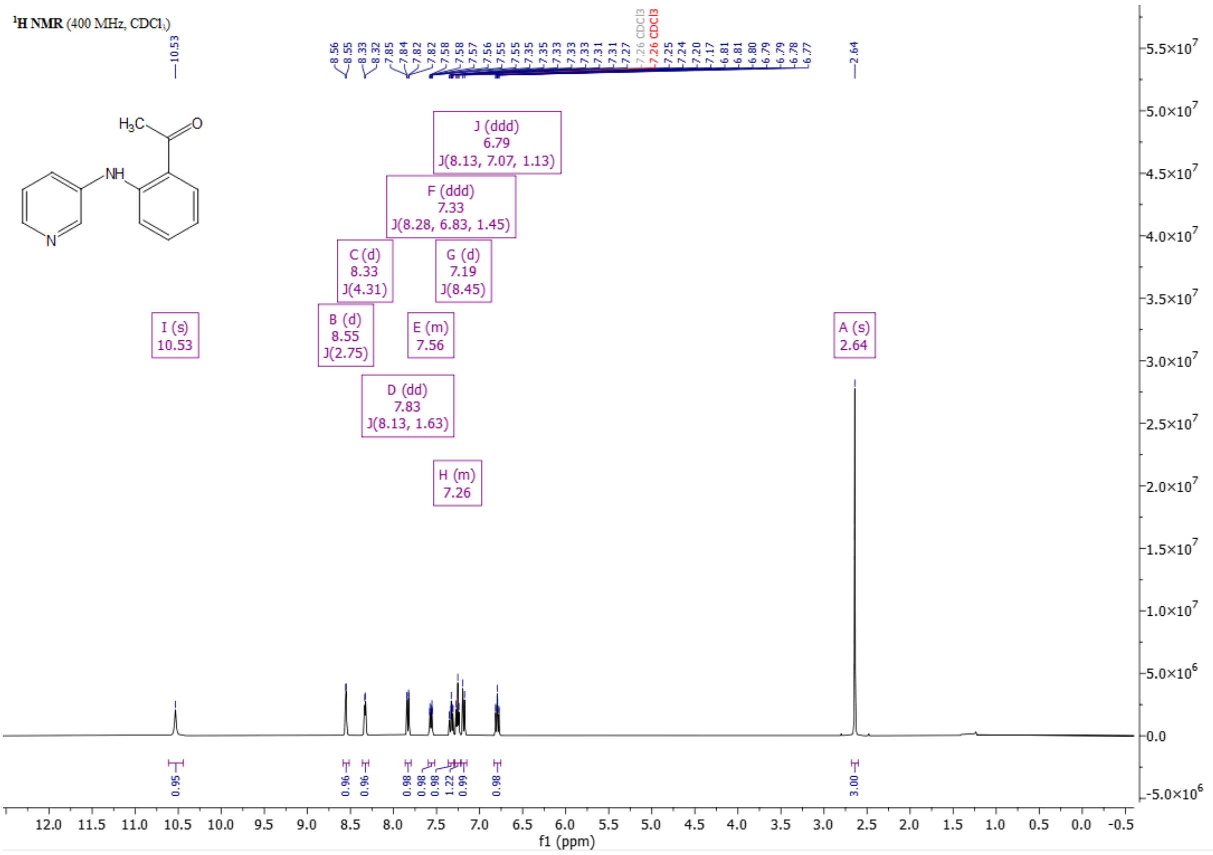

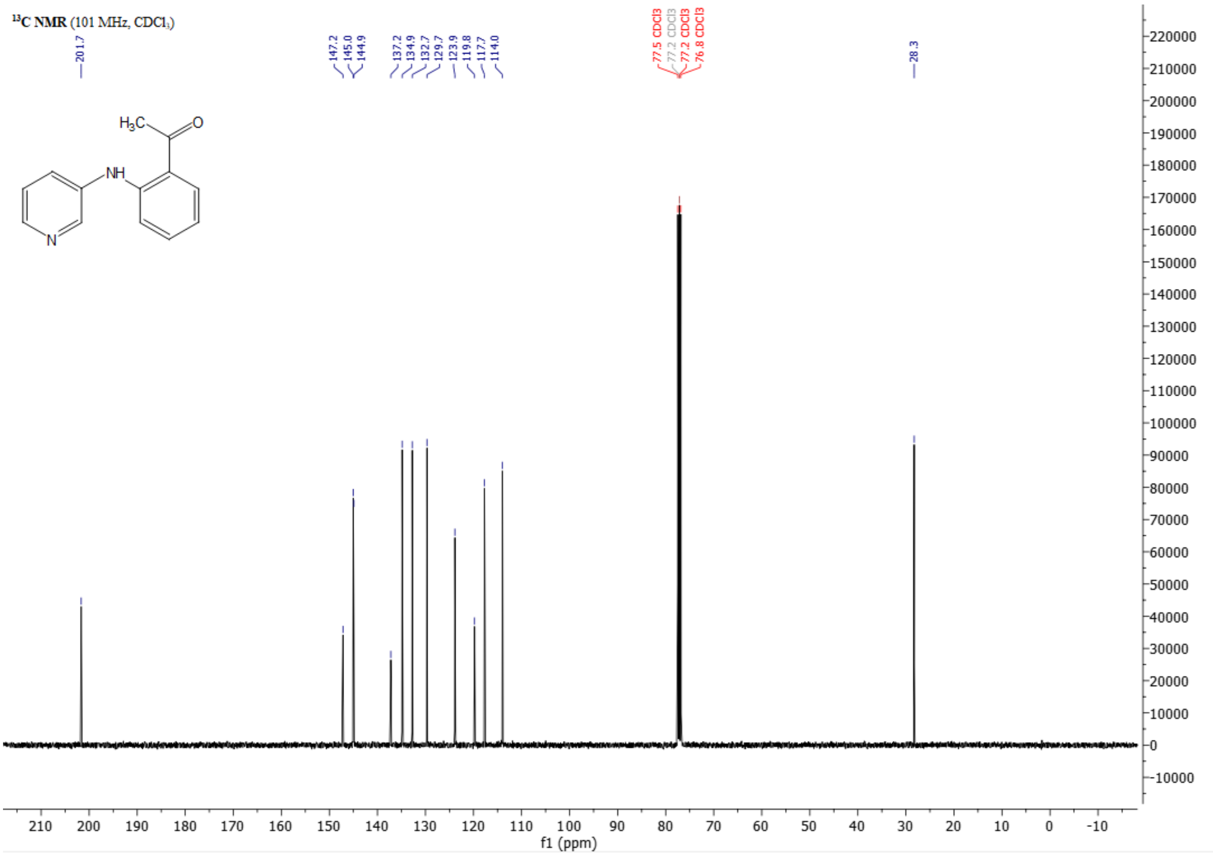

2-(phenylamino)benzaldehyde (18)

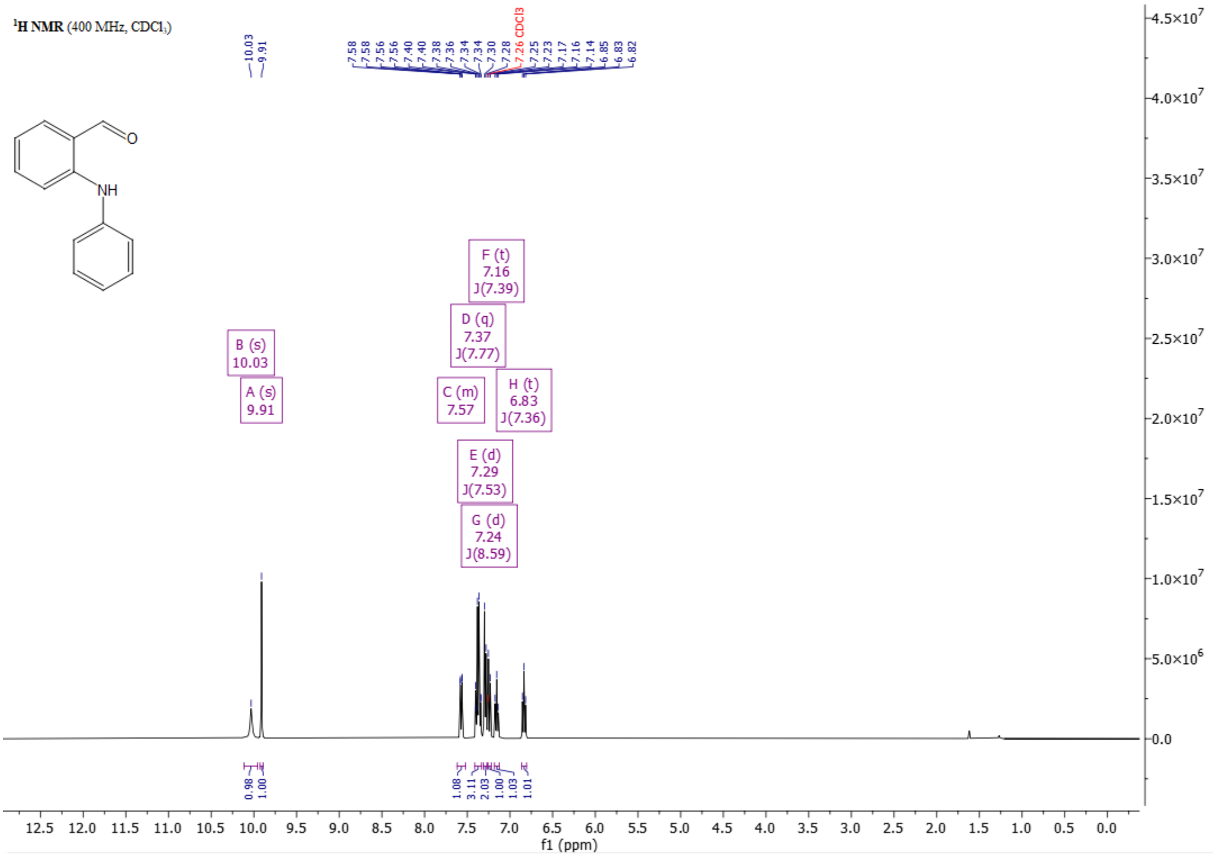

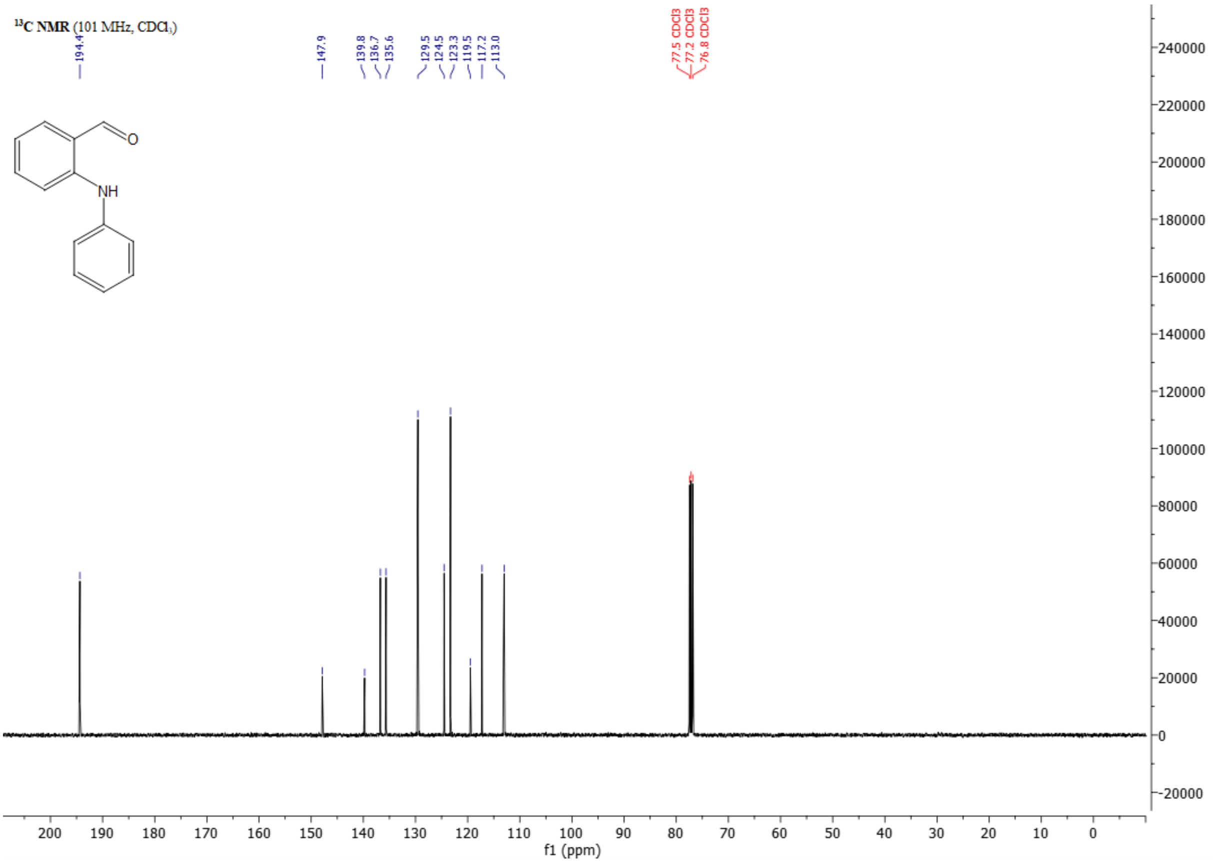

1-(5-fluoro-2-(phenylamino)phenyl)ethan-1-one (19)

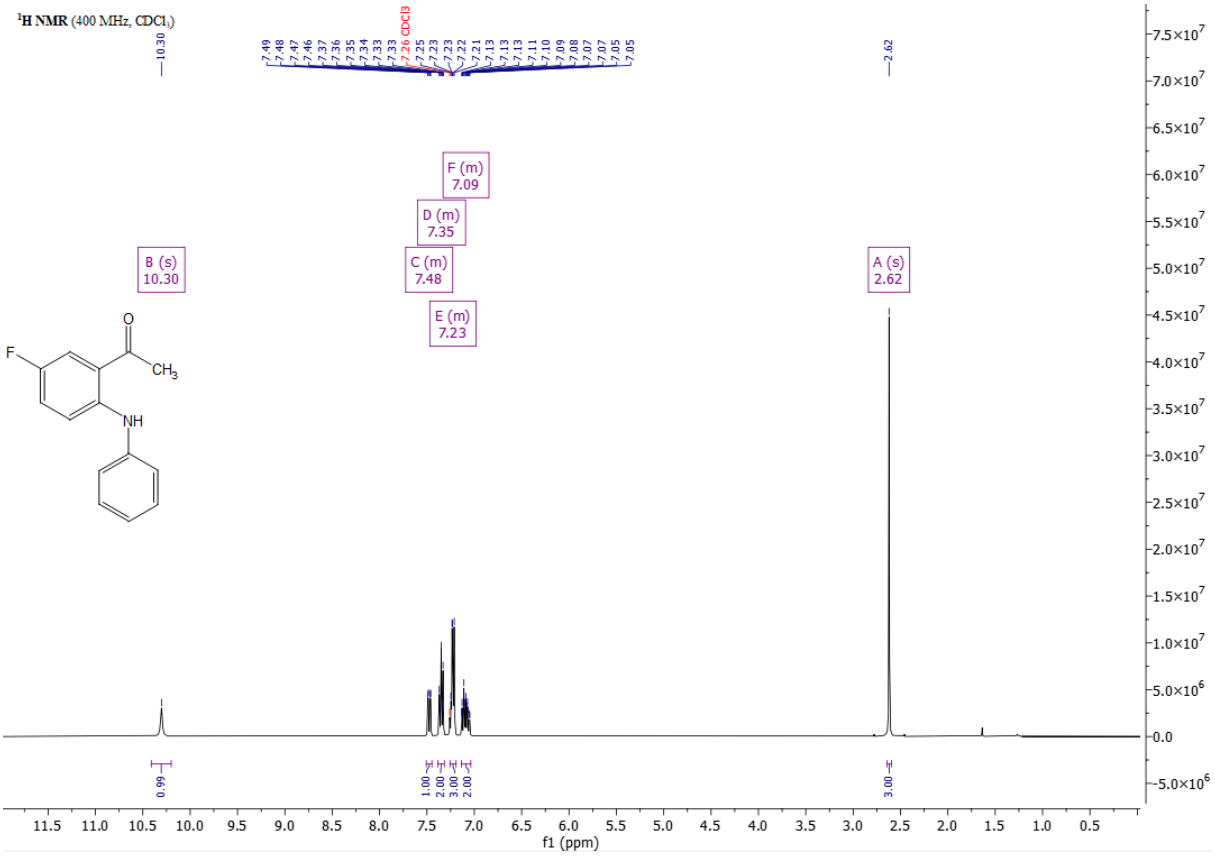

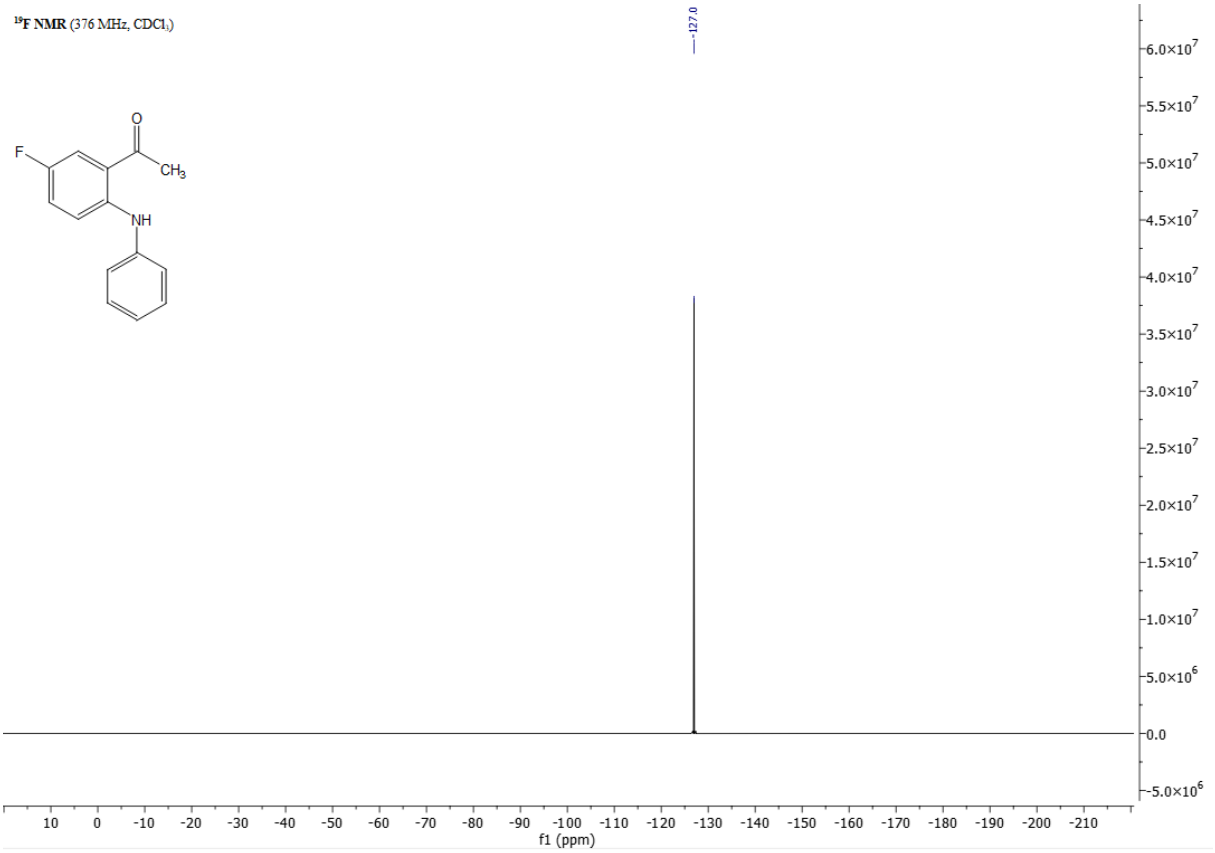

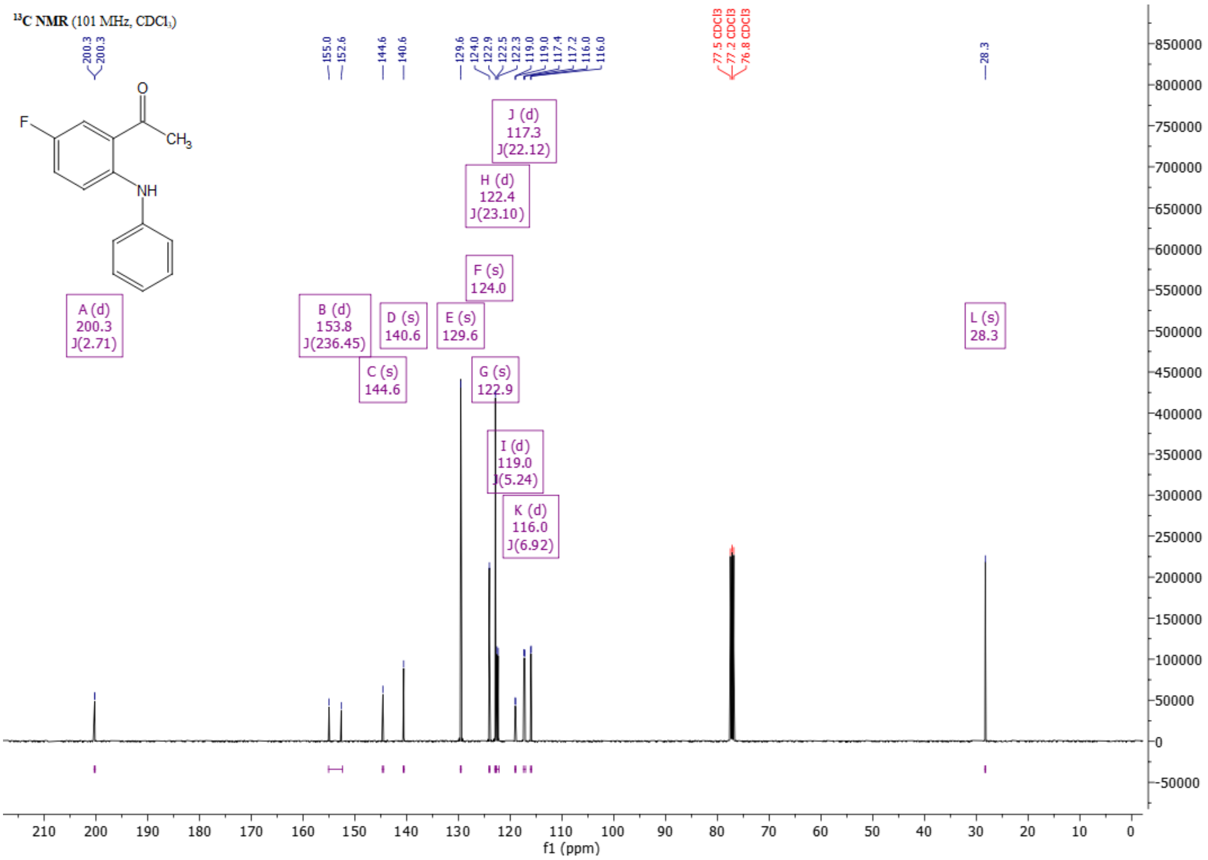

13C NMR (101 MHz, CDCl3)
200.3
200.3
155.0
152.6
144.6
140.6
129.6
124.0
122.9
122.5
122.3
119.0
119.0
117.4
117.2
116.0
116.0
77.5 CDCl3
77.2 CDCl3
76.8 CDCl3
28.3
F
O
CH3
NH
J (d) 117.3 J(22.12)
H (d) 122.4 J(23.10)
F (s) 124.0
A (d) 200.3 J(2.71)
B (d) 153.8 J(236.45)
D (s) 140.6
E (s) 129.6
L (s) 28.3
C (s) 144.6
G (s) 122.9
I (d) 119.0 J(5.24)
K (d) 116.0 J(6.92)
f1 (ppm)

1-(5-chloro-2-(phenylamino)phenyl)ethan-1-one (20)

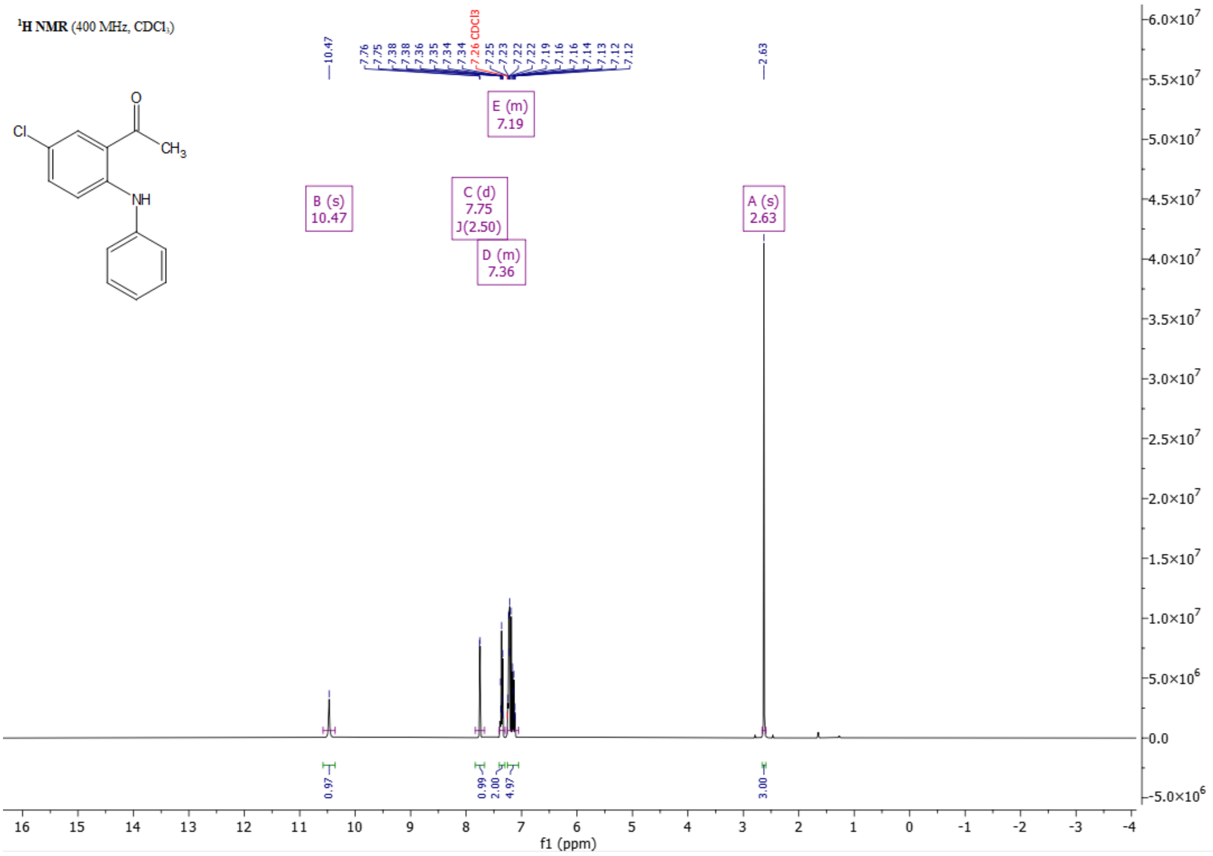

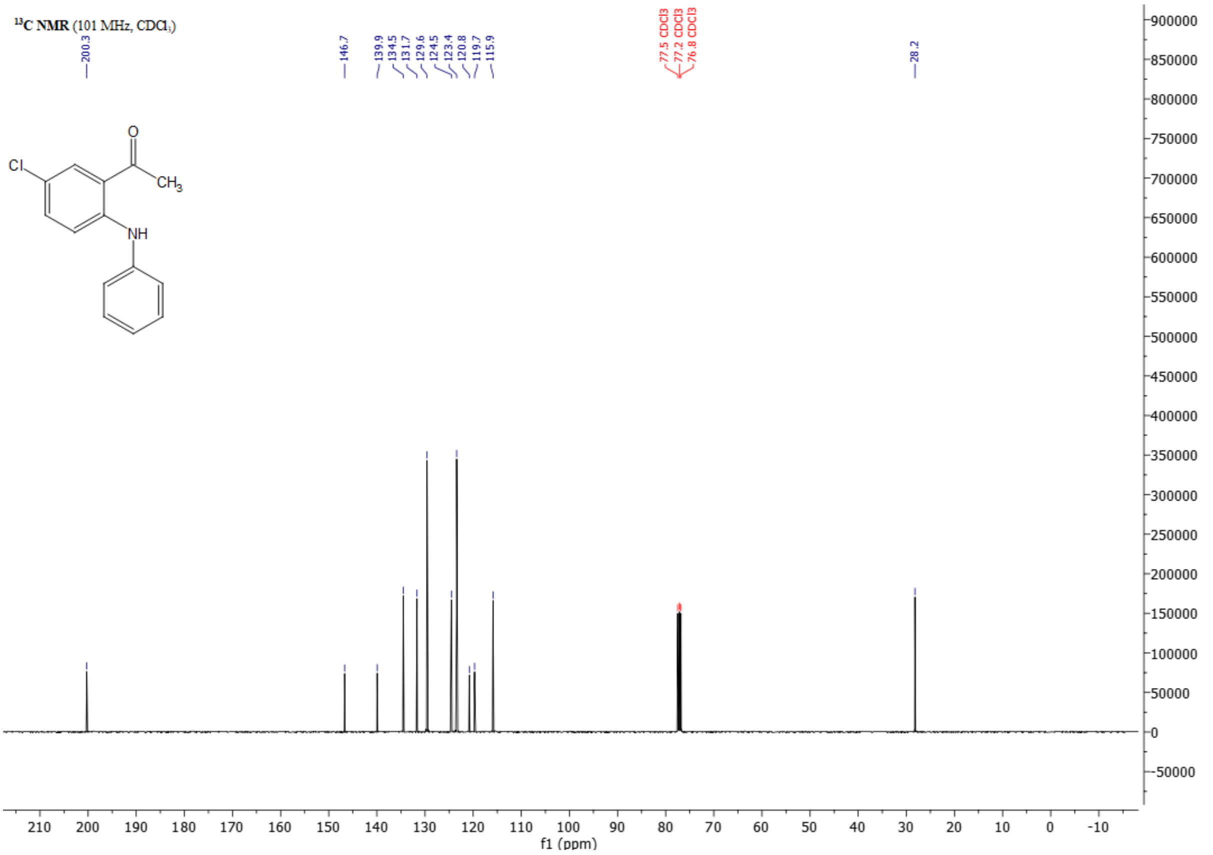

1-(4,5-dimethoxy-2-(phenylamino)phenyl)ethan-1-one (21)

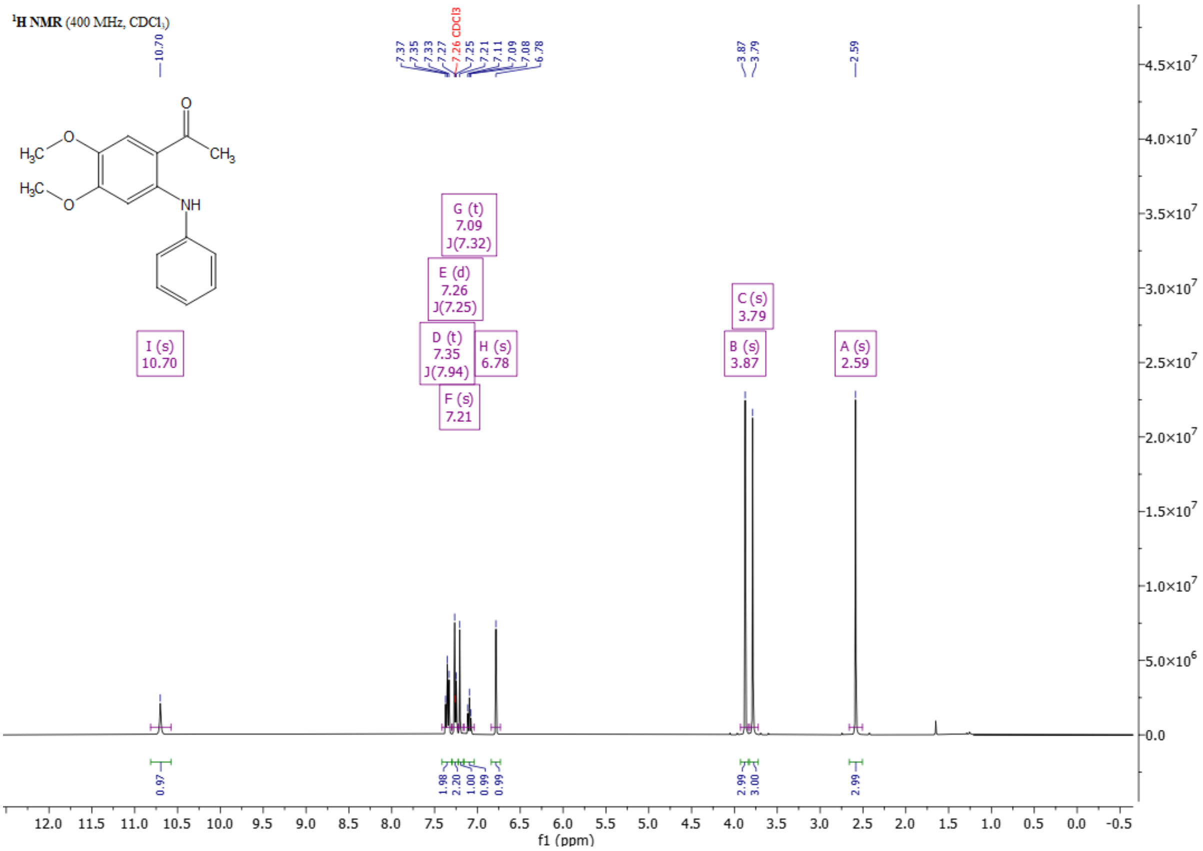

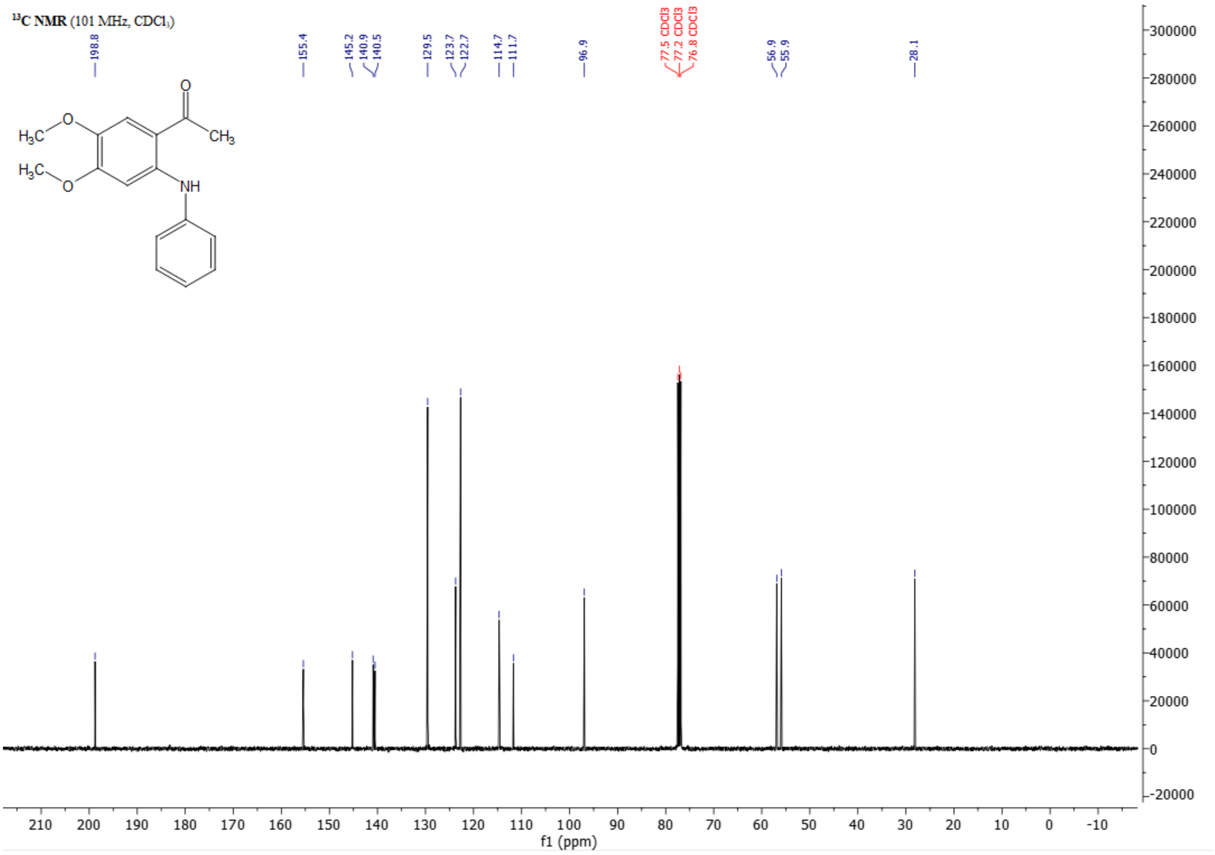

1-(2-(phenylamino)pyridin-3-yl)ethan-1-one (22)

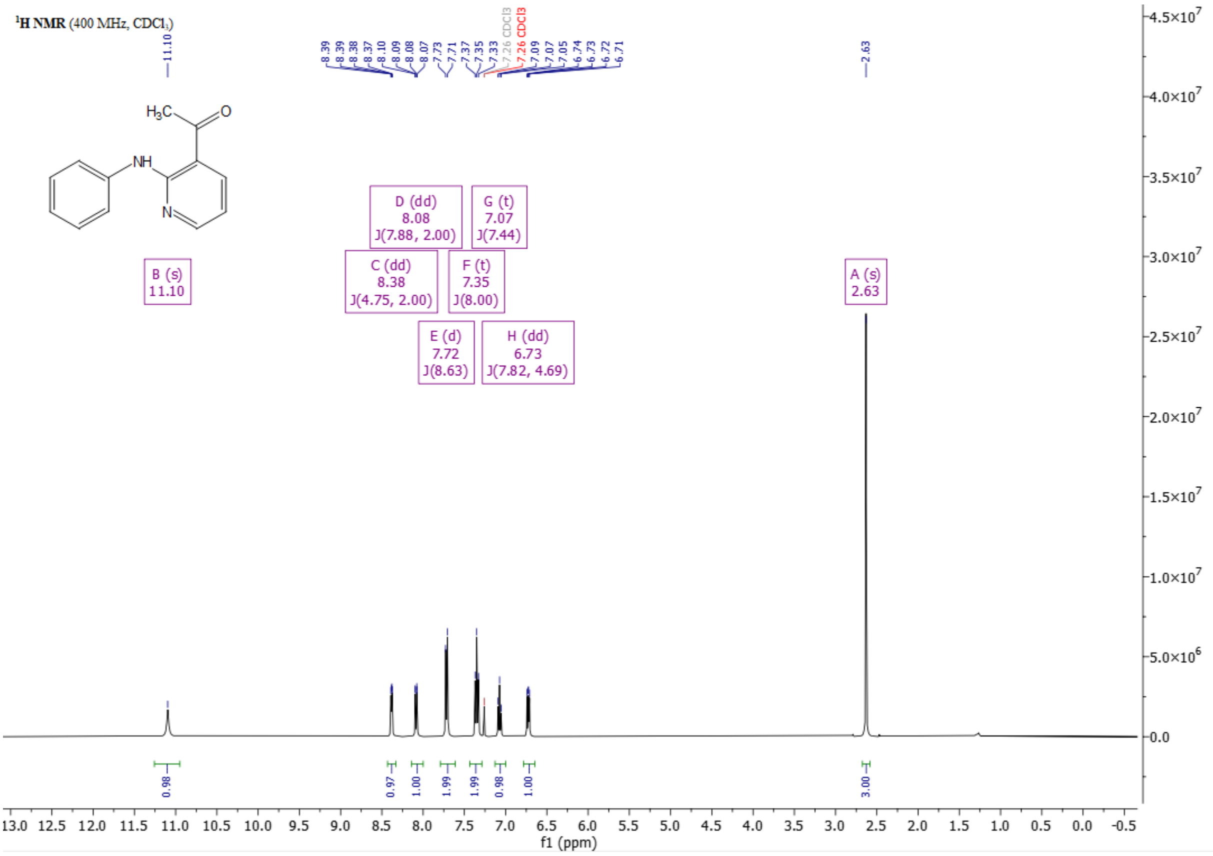

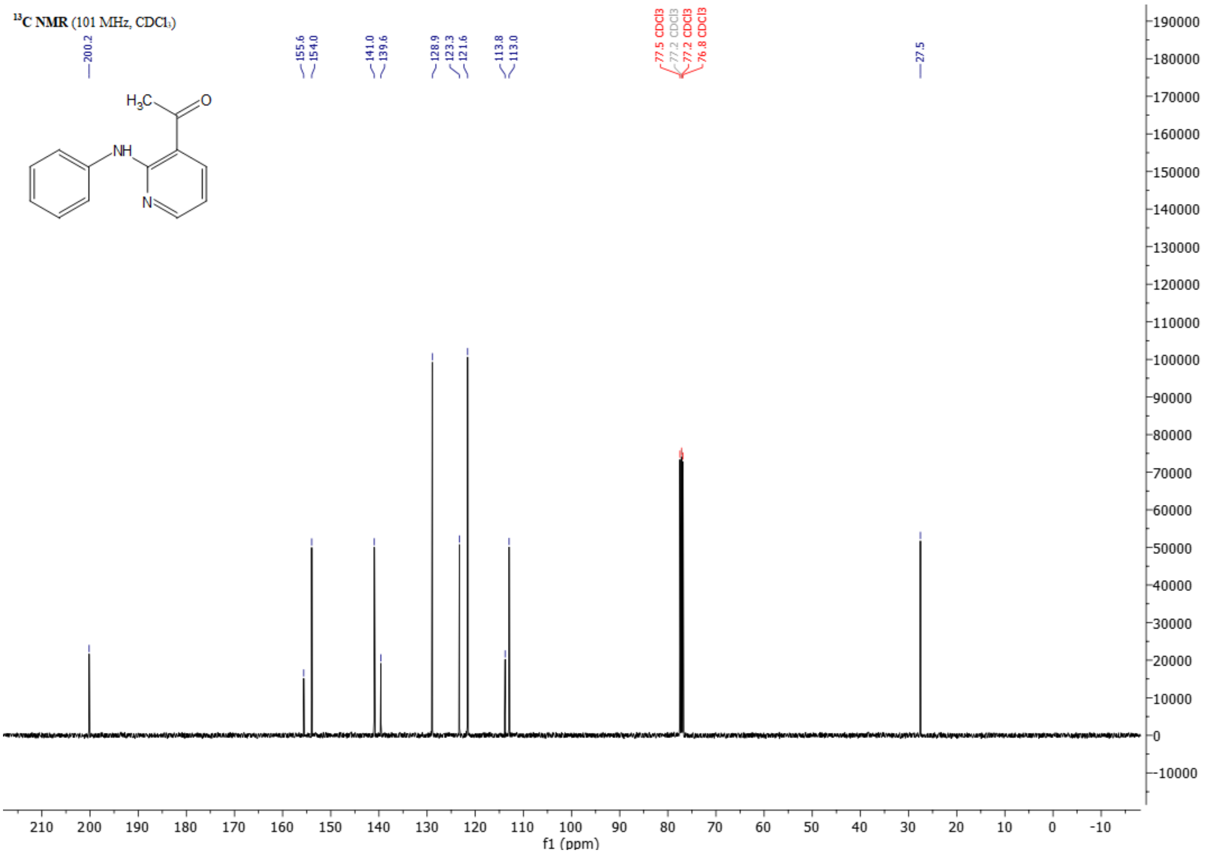

Phenyl(2-(phenylamino)phenyl)methanone (23)

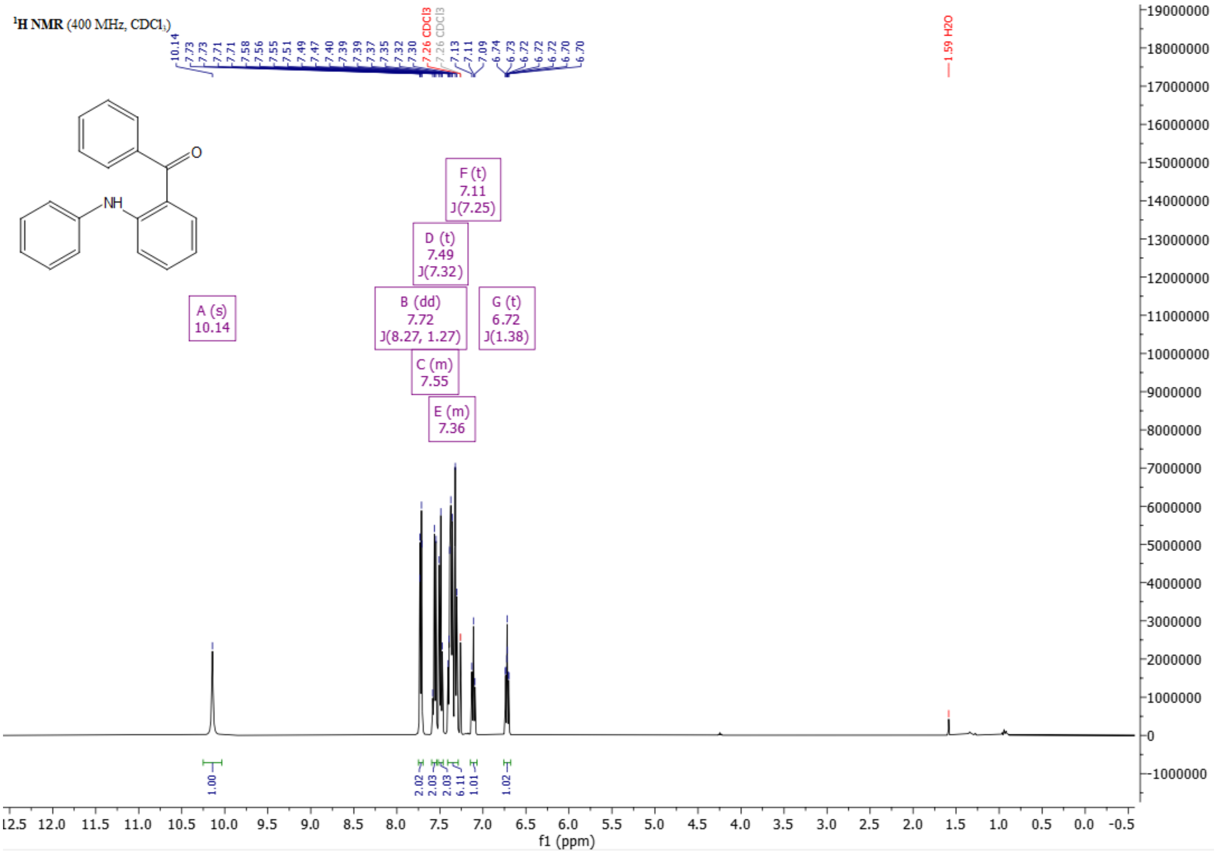

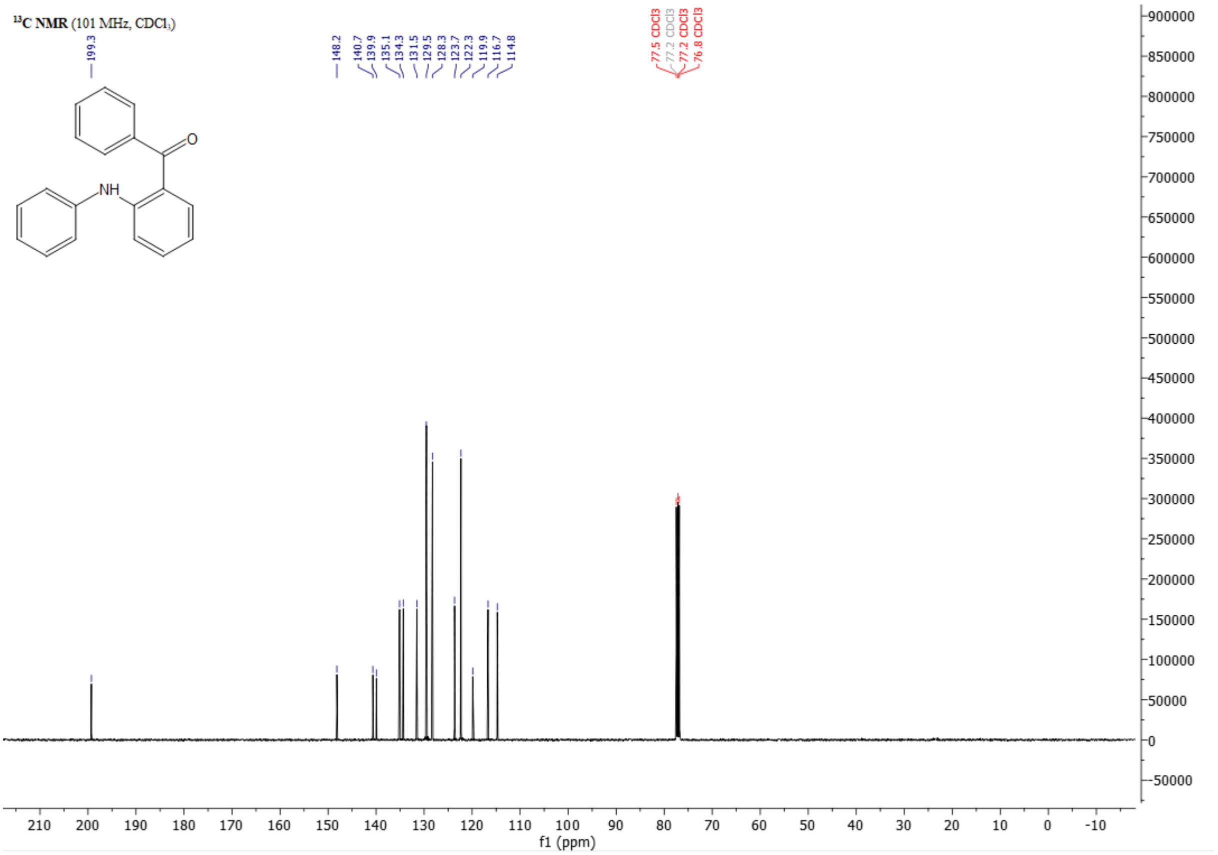

(5-chloro-2-(phenylamino)phenyl)(2-fluorophenyl)methanone (24)

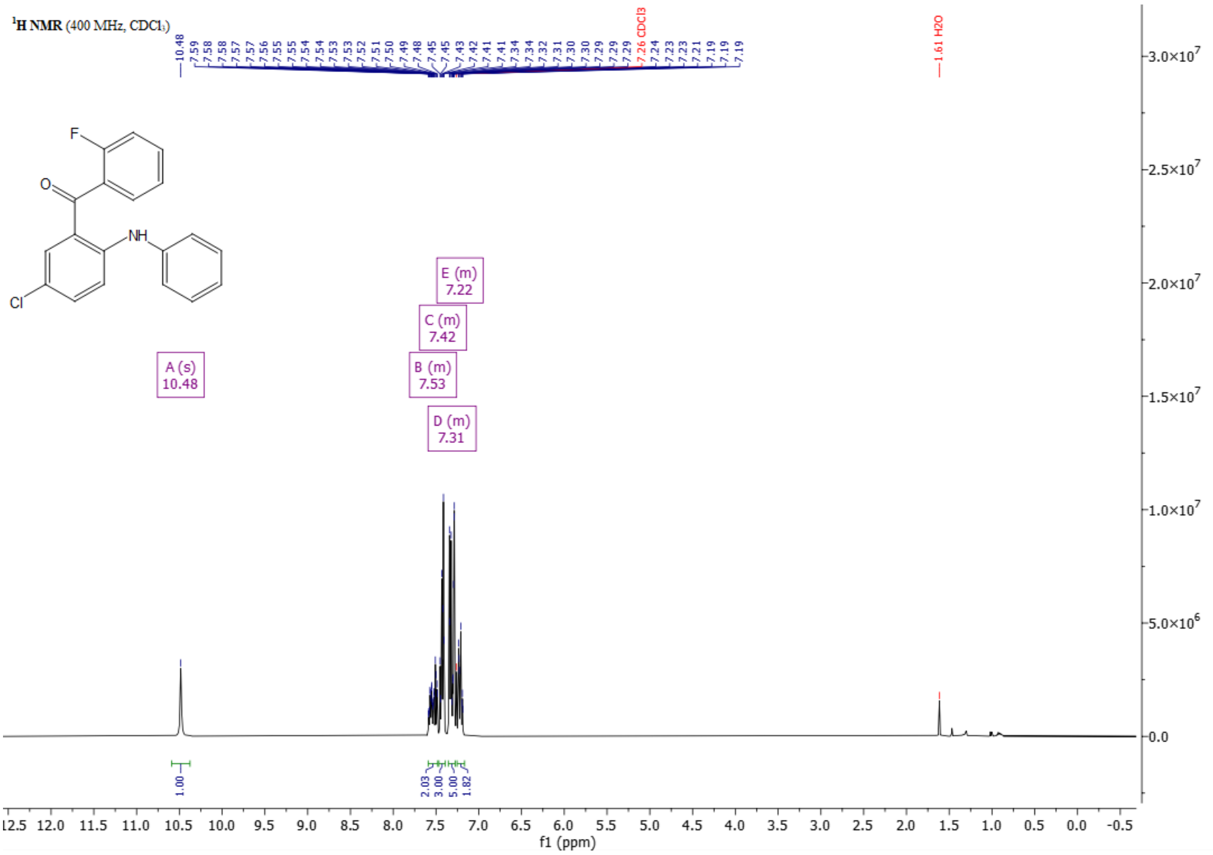

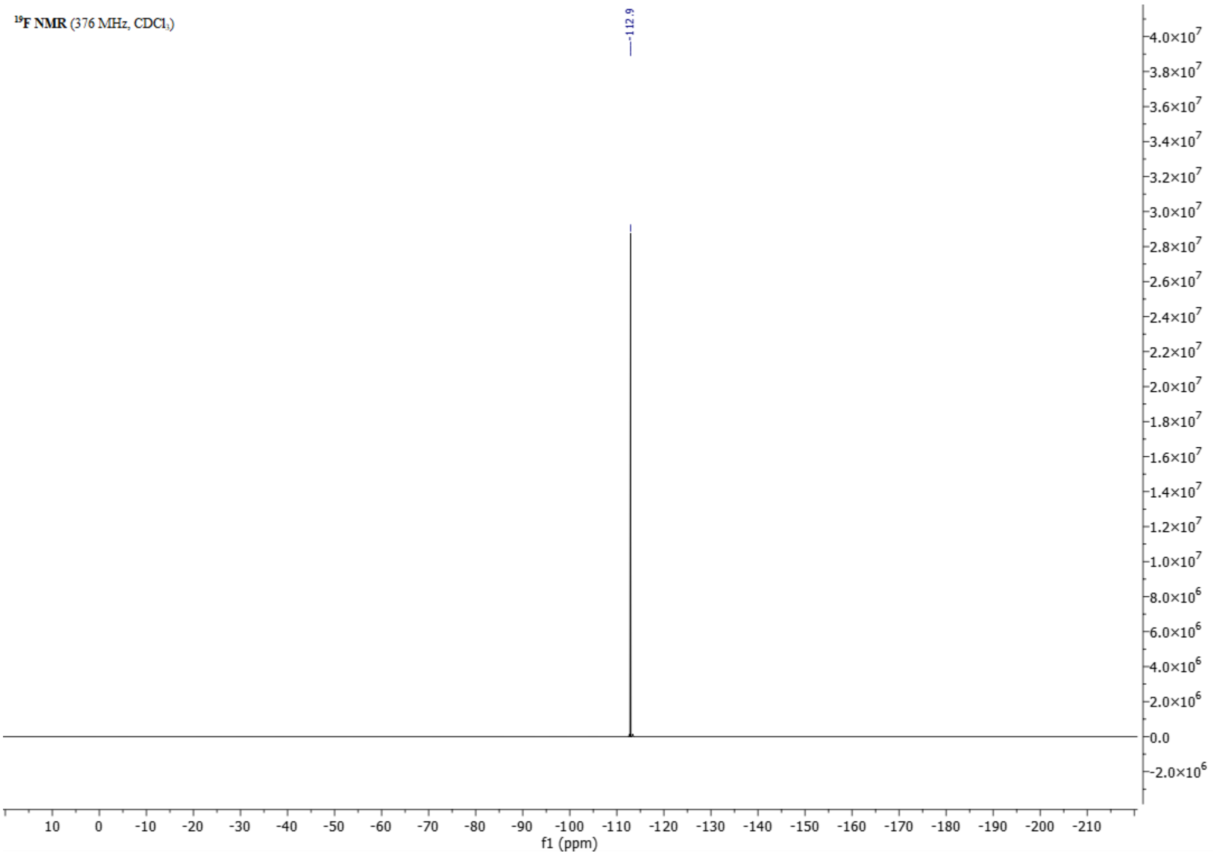

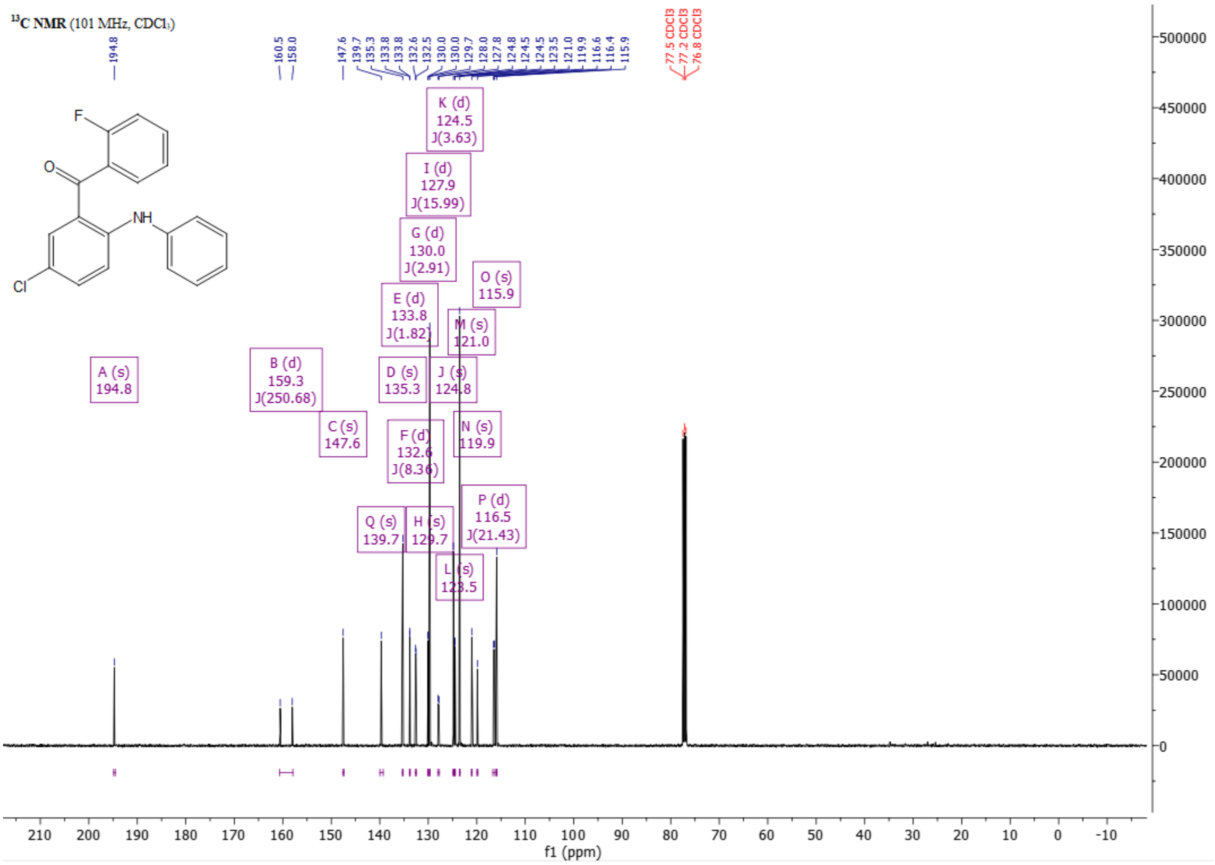

13C NMR (101 MHz, CDCl3)
A (s) 194.8
B (d) 159.3 J(250.68)
C (s) 147.6
D (s) 135.3
E (d) 133.8 J(1.82)
F (d) 132.6 J(8.36)
G (d) 130.0 J(2.91)
H (s) 129.7
I (d) 127.9 J(15.99)
J (s) 124.8
K (d) 124.5 J(3.63)
L (s) 123.5
M (s) 121.0
N (s) 119.9
O (s) 115.9
P (d) 116.5 J(21.43)
Q (s) 139.7
f1 (ppm)

(4-fluorophenyl)(2-(phenylamino)phenyl)methanone (25)

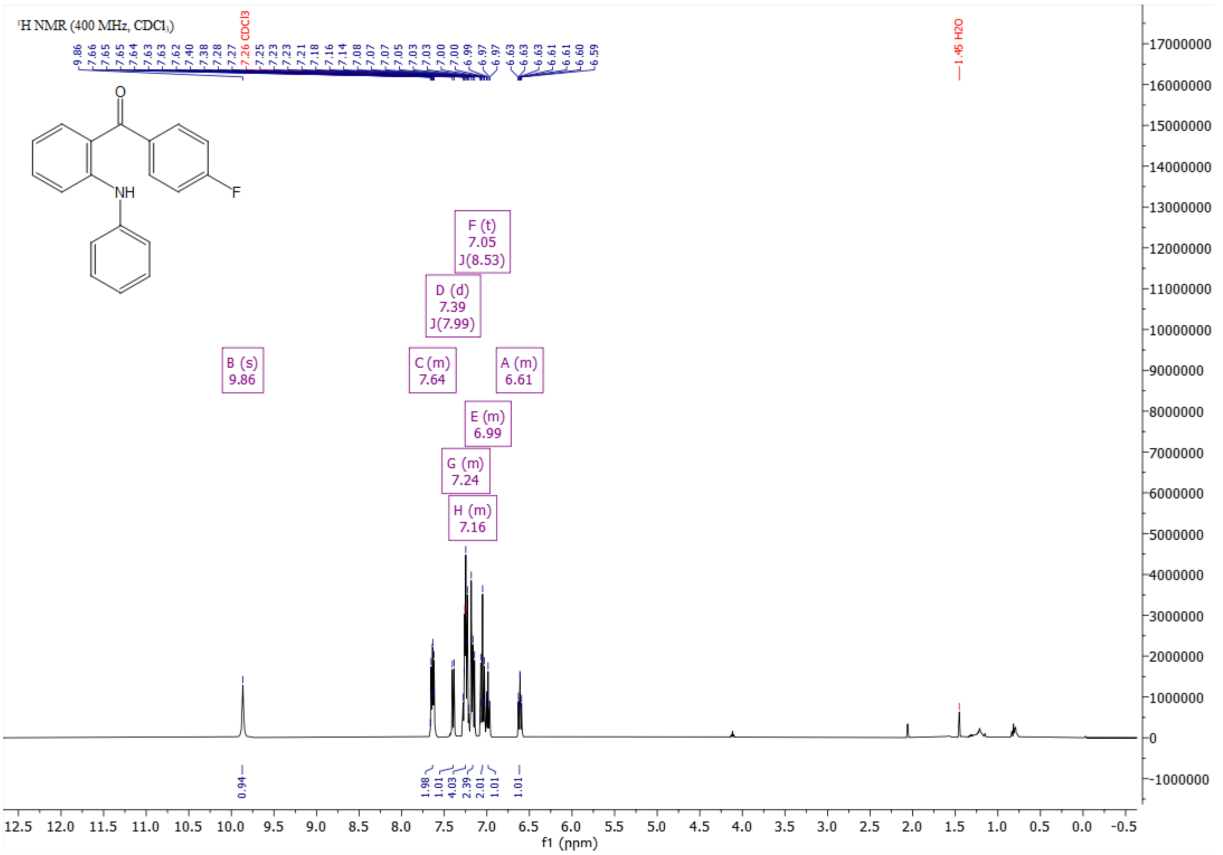

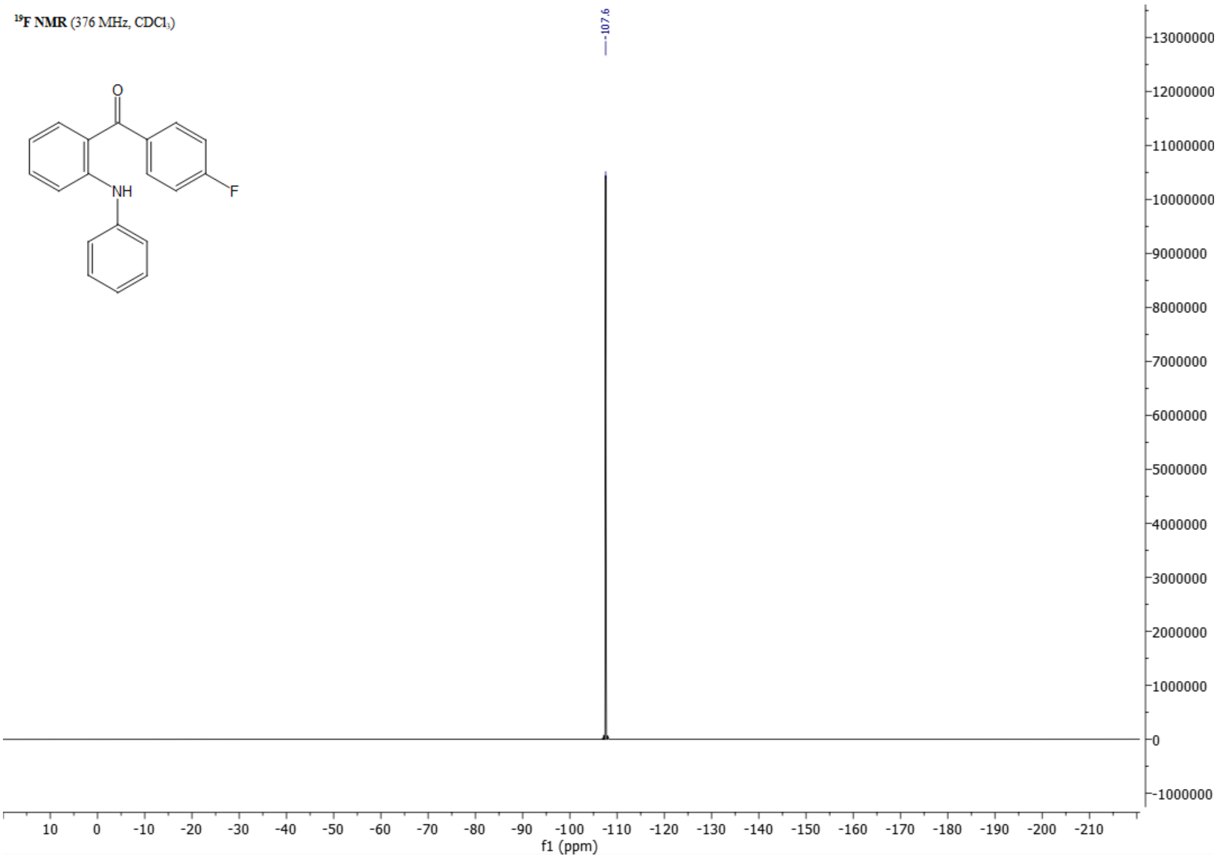

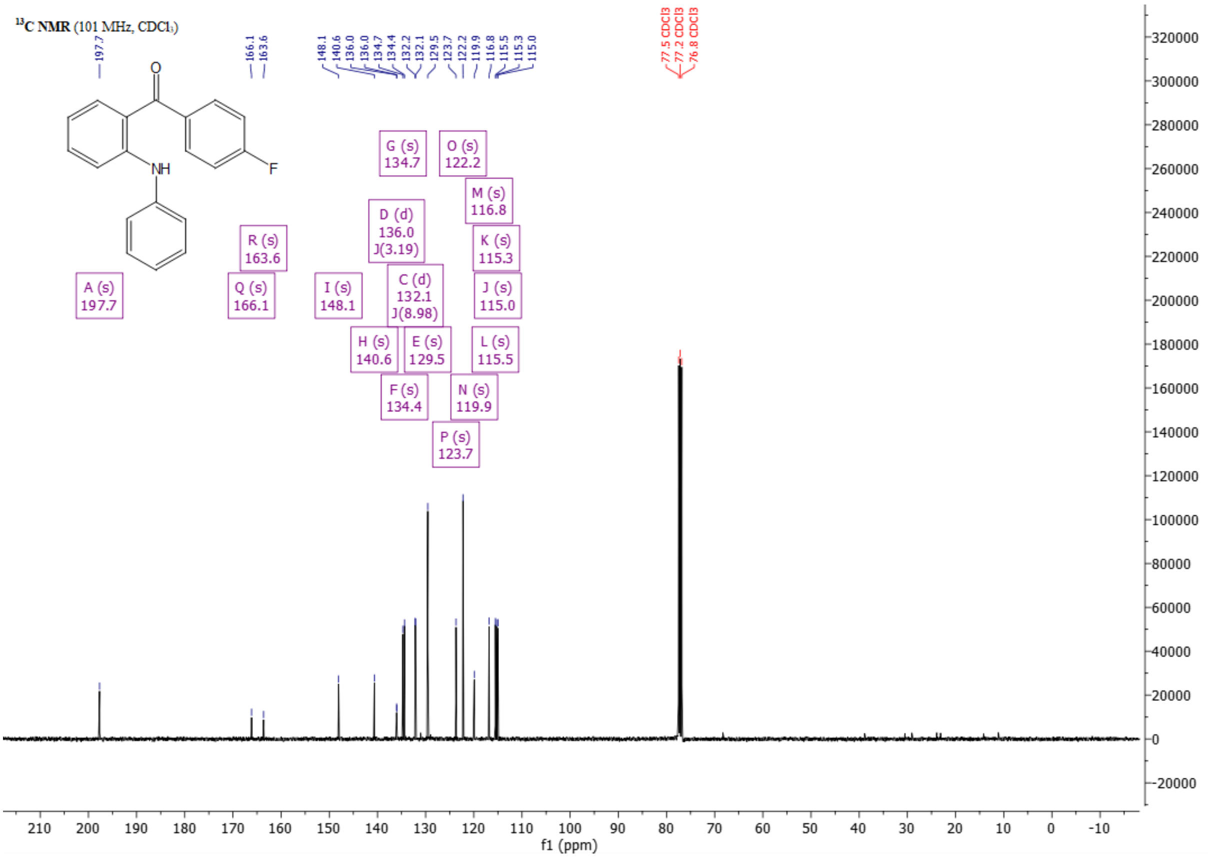

13C NMR (101 MHz, CDCl3)
197.7
166.1
163.6
148.1
140.6
136.0
136.0
134.7
134.4
132.2
132.1
129.5
123.7
122.2
119.9
116.8
115.5
115.3
115.0
77.5 CDCl3
77.2 CDCl3
76.8 CDCl3
O
NH
F
A (s) 197.7
Q (s) 166.1
R (s) 163.6
I (s) 148.1
H (s) 140.6
D (d) 136.0 J(3.19)
G (s) 134.7
F (s) 134.4
C (d) 132.1 J(8.98)
E (s) 129.5
P (s) 123.7
O (s) 122.2
N (s) 119.9
M (s) 116.8
L (s) 115.5
K (s) 115.3
J (s) 115.0
f1 (ppm)

(5-chloro-2-(phenylamino)phenyl)(phenyl)methanone (26)

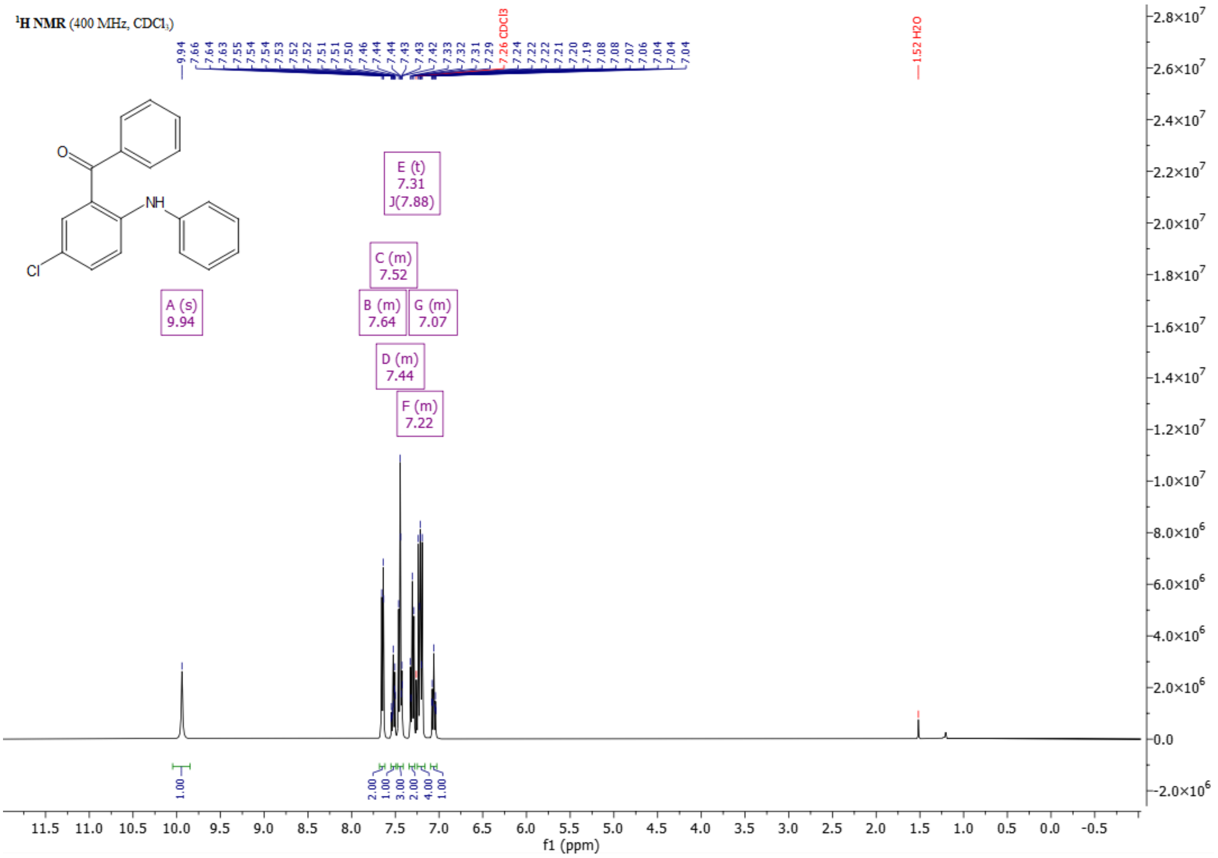

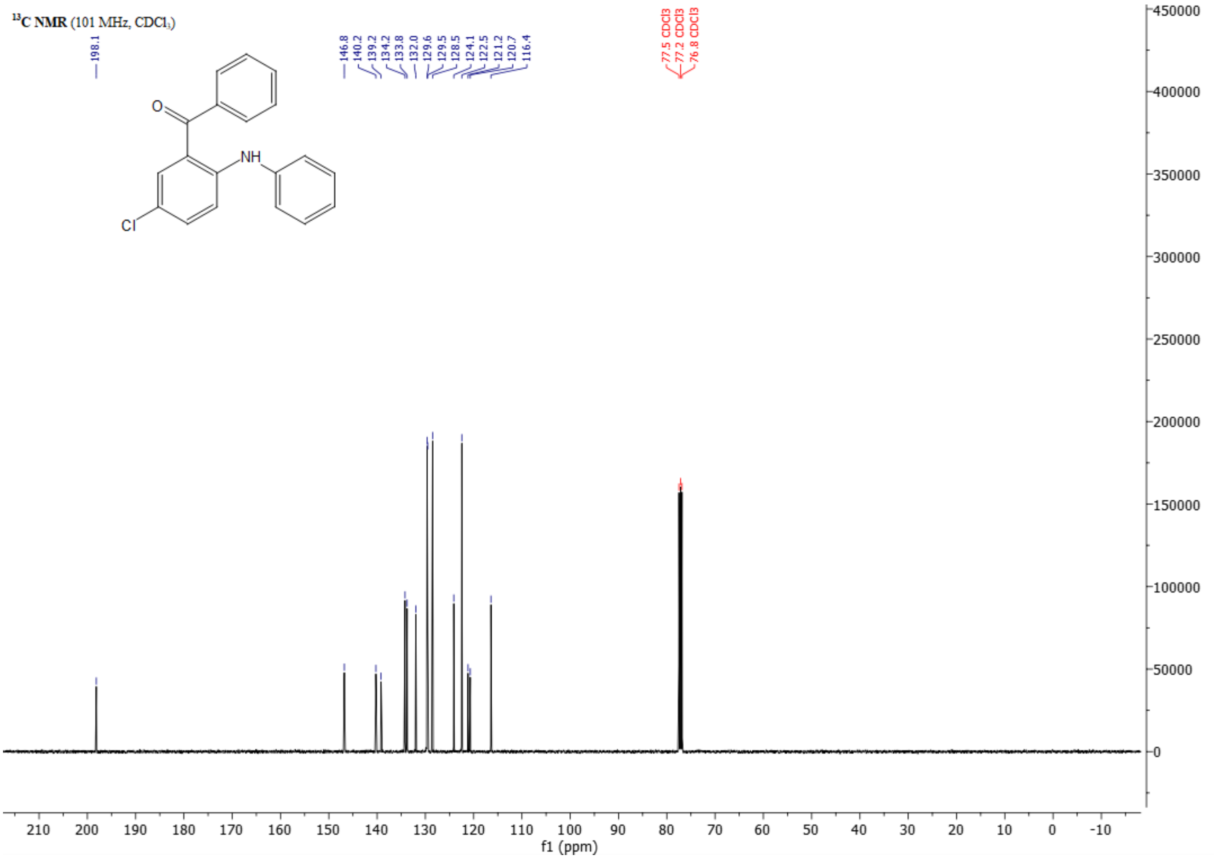

(4-chlorophenyl)(2-(phenylamino)phenyl)methanone (27)

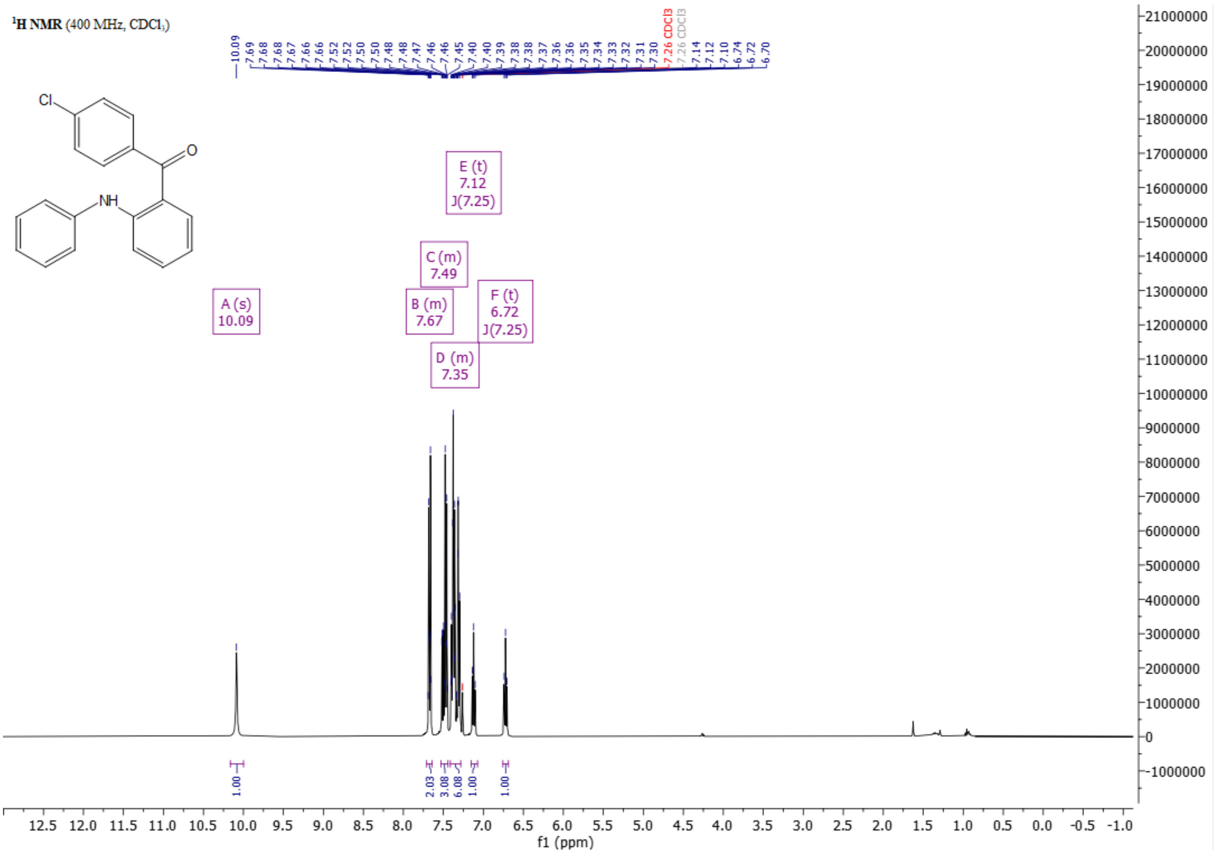

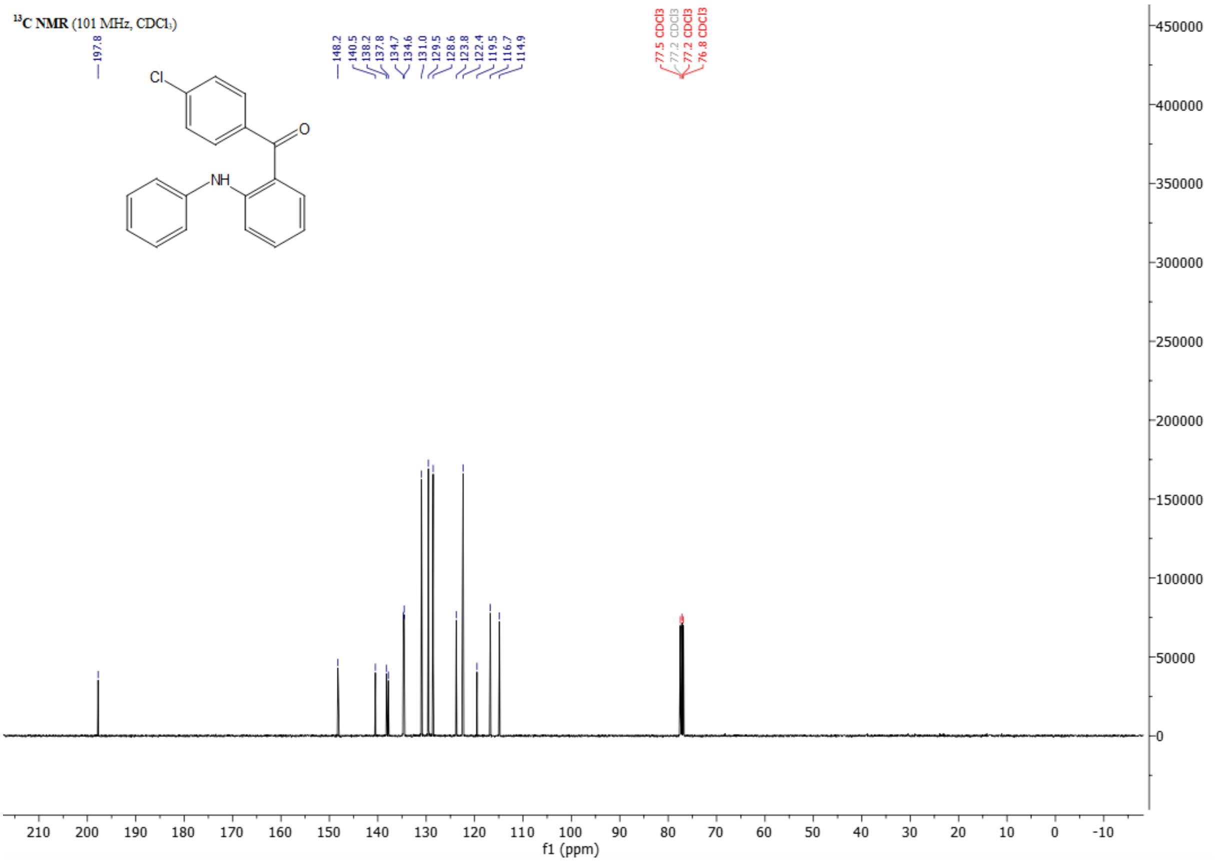

(2-(phenylamino)phenyl)(pyridine-2-yl)methanone (28)

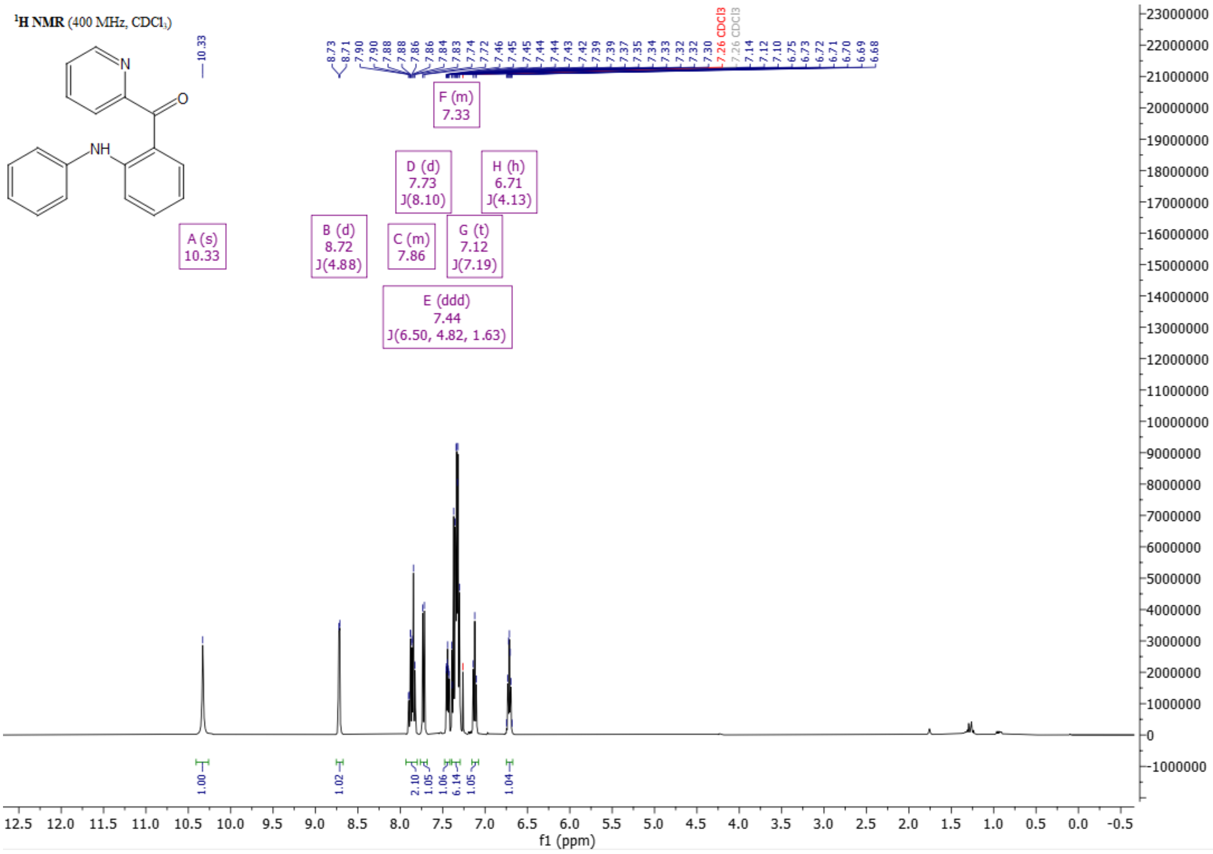

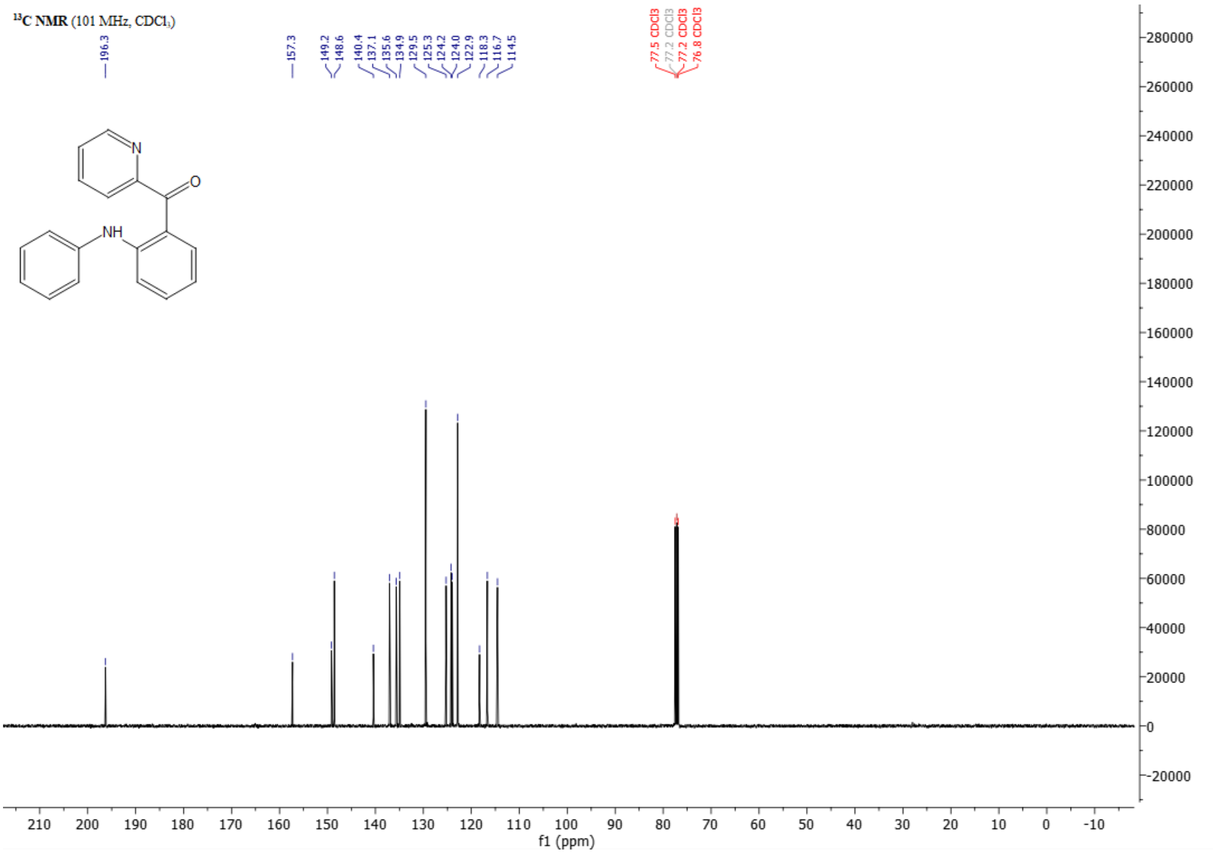

6. References

[87] J. Andraos, M. Sayed, “On the Use of "Green" Metrics in the Undergraduate Organic Chemistry Lecture and Lab To Assess the Mass Efficiency of Organic Reactions” *J. Chem. Educ.* **2007**, *84*, 6, 1004

[88] J. Huang, T. Mao, Q. Zhu, “Copper-Catalyzed Intramolecular Oxidative C(sp^3)–H Amidation of 2-Aminoacetophenones: Efficient Synthesis of Indoline-2,3-diones” *Eur. J. Org. Chem.* **2014**, *2014*, 2878–2882.

[89] B. C. Wray, J. P. Stambuli, “Synthesis of N-Arylindazoles and Benzimidazoles from a Common Intermediate” *Org. Lett.* **2010**, *12*, 4576–4579.

[90] D. Hellwinkel, P. Ittemann, “12-Organyldibenz[*b,g*]azocin-5,7-dione” *Ber. Dtsch. Chem. Ges.* **1986**, *119*, 3165-3197.

[91] F. Zhou, L. Zhang, J. Shi, “A terphenyl phosphine as a highly efficient ligand for palladium-catalysed amination of aryl halides with 1° anilines” *J. Catal.* **2021**, *402*, 238-243.

[92] I. Ghosh, N. Shlapakov, T. A. Karl, J. Düker, M. Nikitin, J. V. Burykina, V. P. Ananikov, B. König, “General cross-coupling reactions with adaptive dynamic homogeneous catalysis” *Nature* **2023**, *619*, 87–93.

[93] J. Li, E. Tan, N. Keller, Y. H. Chen, P. M. Zehetmaier, A. C. Jakowetz, T. Bein, P. Knochel, “Cobalt-Catalyzed Electrophilic Aminations with Anthranils: An Expedient Route to Condensed Quinolines” *J. Am. Chem. Soc.* **2019**, *141*, 98–103.
